# The rising entropy of English in the attention economy

**DOI:** 10.1038/s44271-024-00117-1

**Published:** 2024-08-01

**Authors:** Charlie Pilgrim, Weisi Guo, Thomas T. Hills

**Affiliations:** 1 Mathematics, University of Leeds, Leeds, UK; 2 The Mathematics of Real-World Systems CDT, The University of Warwick, Coventry, UK; 3 Experimental Psychology, University College London, London, UK; 4 The Alan Turing Institute, London, UK; 5 Human Machine Intelligence Group, Cranfield University, Bedford, UK; 6 Department of Psychology, The University of Warwick, Coventry, UK

**Keywords:** Language and linguistics, Human behaviour, Psychology, Cultural and media studies, Language and linguistics

## Abstract

We present evidence that the word entropy of American English has been rising steadily since around 1900. We also find differences in word entropy between media categories, with short-form media such as news and magazines having higher entropy than long-form media, and social media feeds having higher entropy still. To explain these results we develop an ecological model of the attention economy that combines ideas from Zipf’s law and information foraging. In this model, media consumers maximize information utility rate taking into account the costs of information search, while media producers adapt to technologies that reduce search costs, driving them to generate higher entropy content in increasingly shorter formats.

## Abstract (web summary)

American English has become more information dense over the last 100 years, likely driven by competition for human attention.

## Introduction

Word entropy is a measure of the amount of repetition (low entropy) or novelty (high entropy) in word distributions. Empirical word distributions typically follow Zipf’s law, which describes a power law between a word’s observed frequency and that word’s rank in the frequency distribution^1^. This empirical power law is remarkably stable with an exponent around 1^2–4^. The stability of Zipf’s law suggests some underlying mechanism, and Zipf himself hypothesised a *principle of least effort* between speakers and listeners. More recently this principle has been expanded to show that power laws in word distributions can emerge from a balance between maximising the benefits of receiving highly informative messages (preferred by listeners) and minimising the costs of generating high word entropy text (preferred by speakers)^4^.

In recent times this balance between the efforts of listeners and speakers has changed. Modern communication systems have transformed the way that we share and consume information, in particular by increasing the accessibility of information^5^. In the words of Herbert Simon, this creates a “poverty of attention”^6^, such that media producers must compete for the limited resource of human attention^7,8^. This dynamic has been called *the attention economy*, a combination of forces influencing the production and consumption of information, with consequences including a shortening collective attention span^9^. If information adapts to the balance between the preferences of media producers and consumers, then increased competition for attention tips the balance toward the preferences of the consumers. That is, information markets (the distribution of available content) should rise in information density. Specifically, we quantify increasing information density as higher entropy, reflecting more diverse and less predictable information.

We can envision this adaptive process in terms of *information foraging*^10,11^. Information foraging describes how people search for and consume information in different environments, including web browsing^12^ software debugging^13–15^, and the design of information and social environments^12,15,16^. The basic rationale of this approach is borrowed from ecological models of foraging, which have been shown to be appropriate to a wide range of search problems ranging from spatial foraging to cultural evolution^17^. Indeed, handling the exploration versus exploitation trade-off that is common to all of these environments has been proposed to be a defining selective force in the evolution of cognition^18,19^.

In what follows, we first investigate the evolution of information across a wide variety of media sources over the last two centuries, a time marked by increasing media competition. We show how this reveals a characteristic pattern of rising entropy that affects different categories of media in different ways (e.g., books versus news versus social media). We then create a model of the attention economy that expands on existing models of information foraging to incorporate competition for human attention between media producers. This model explains both the general increase in word entropy and the differences in word entropy across categories.

## Methods

### Text corpora

To investigate the recent history of information evolution we examine a variety of text corpora. The Corpus of Historical American English (COHA)^20,21^ has 116,614 texts spanning the 1810s to 2000s, balanced between categories of fiction (*n* = 11,010), non-fiction (*n* = 2635), news (*n* = 41,677) and magazines (*n* = 61,292). The Corpus of Contemporary American English (COCA)^22,23^ has over 150,000 texts from between 1990 to 2008 split between fiction, popular magazines, newspapers, academic journals and spoken word. For our analysis, we used a publicly available sample of COCA with 2362 texts split between categories of fiction (*n* = 275), academic journals (*n* = 266), news (*n* = 872) and magazines (*n* = 949). The British National Corpus (BNC)^24^ contains 8098 texts from between 1960 and 1993 including written categories of fiction (*n* = 904), academic prose (*n* = 994), newspapers (*n* = 972), non-academic prose and biography, other published materials and unpublished materials. Fiction and newspapers are common categories across the corpora. Magazines are a common category between COHA and COCA. We grouped as non-fiction the categories of COHA non-fiction, COCA academic journals and BNC academic prose.

The lexical measures (details below) are all sensitive to sample size. We, therefore, chose to truncate text samples to *N* = 2000 words, and discarded text samples with less words. This word count strikes a good balance of being a large enough sample size to detect variation between texts, while being small enough that we have a large number of surviving texts in the corpora.

The details of the text cleaning are described below. The cleaned datasets had the following surviving sample counts with *N* = 2000 words:

- COHA total *n* = 22,233. Fiction *n* = 8162, non-fiction *n* = 2045, news *n* = 720, magazines *n* = 11,306.
- COCA total *n* = 985. Fiction *n* = 167, non-fiction *n* = 166, news *n* = 39, magazines *n* = 133.
- BNC total *n* = 1319. Fiction *n* = 447, non-fiction *n* = 477, news *n* = 395.

The COHA dataset was analysed as a time series, so requires a large number of samples. The BNC and COCA, being corpora from much narrower time ranges, were analysed as distributions and as such require less samples.

#### Data cleaning

The text sample data was cleaned before analysis in a standard way^25^. COHA and COCA are similar formats and so followed the same procedure. For both:

- Stripped any headers not a part of the main text samples.
- Removed any XML text tags.
- Removed any sentences that contained “@” symbols. COHA and COCA randomly replace words with @ symbol in groups of ten for copyright reasons^26^.
- Removed apostrophes and extra whitespace.
- Used python’s natural language toolkit (nltk) package to convert text to tokens^27^.
- Removed any non-text tokens (containing punctuation, numbers or special symbols).
- Converted all tokens to lowercase.
- Filtered out any text samples with less than 2100 tokens (words).
- Selected the middle 2000 tokens of the text sample for processing. This avoids, as much as possible, anomalous text that sometimes appears at the start or end of text samples such as a contents section or copyright notices.

For the BNC data, python’s natural language toolkit package comes with a BNC corpus reader^27^, which was used to extract tokens. The only other treatment was to remove extra whitespace and apostrophes as with COCA and COHA.

#### Social media data

We also investigated social media, considering Twitter (now X) datasets from 2009 and 2020, and a Reddit dataset from 2024.

The Twitter 2009 dataset consisted of 1.6 million tweets downloaded from the Twitter API between April and June 2009^28^ and available online at https://www.kaggle.com/kazanova/sentiment140. To simulate a Twitter feed the tweets were chronologically collated to create *n* = 9180 text samples with *N* = 2000 words each.

The Twitter 2020 dataset contained 16 million unique tweets collected from the Twitter API between February 2020 and January 2021. This data was originally downloaded and used in a study on political polarisation^29^, with tweets selected from followers of well-known US news sources (full details of data collection is in the original paper). We simulated a Twitter feed by chronologically collating these tweets, generating *n* = 143,045 text samples with *N* = 2000 words each.

For Reddit, we aimed to capture text samples that were representative of the text a user would see when visiting the site. To achieve this we used Reddit’s API to download posts from the Reddit homepage feed at https://oauth.reddit.com/.json from 15th to 17th January 2024. We downloaded 14,892 unique English posts in JSON format in this way. We extracted the text from the posts and combined them to create *n* = 222 text samples with length *N* = 2000 words each. During processing, we found a small number of non-English posts in the feed, which were removed.

The social media data was cleaned in the same way as the COHA and COCA data. The processing steps act to remove any website and email addresses, hashtags and site-specific usernames.

We chose text samples of *N* = 2000 words to match the text sample sizes used when analysing other corpora, as the textual measures are sensitive to sample size. Social media statuses are by nature short and are usually much smaller than *N* = 2000 words, and lexical measures of short text samples have little meaning. Our analysis is on the level of the social media feed and we generated large text samples through the collation of posts. This kind of collation will naturally create text samples with high entropy as a result of the changing contexts of social media posts (as well as high type token ratio and low Zipf exponent). This isn’t a flawed analysis—the high information density of a social media feed is related to the collation of statuses and how people actually consume social media.

#### Measures of information evolution

Information evolution is measured using unigram word entropy. For robustness, we also analysed the type token ratio and Zipf exponent, which are alternative measures of word frequency distributions. See the Limitations section for a further discussion of the text measures used.

Empirical unigram word entropy, *H*_1_, is a function of the relative frequencies of each word, *f*_*i*_, summed over the set of *W* unique words in the text sample.

1 $${H}_{1}=-\sum\limits_{i=1}^{W}{f}_{i}log{f}_{i}\,.$$

Type token ratio (TTR) is the number of unique words (types) divided by the total number of words (tokens) in a text sample.

2 $$TTR=\frac{\#types}{\#tokens}\,.$$

Words in natural language are typically approximately distributed as a power law distribution between type frequency, *f*_*i*_, and type rank in that frequency distribution, *r*(*f*_*i*_)^30^. This power law is parameterised by the Zipf exponent, *α*, which describes the steepness of the distribution in log space. Maximum likelihood estimation was used to estimate the Zipf exponent^30^. This estimator has the benefit of being well known and widely used.

3 $${f}_{i}\propto r{({f}_{i})}^{-\alpha }\,.$$

Each of the measures were applied once to the same set of distinct text samples.

#### Data exclusions

We removed outliers that had values of lexical measures that were more than 5 standard deviations from the mean of the corpus (less than 0.1% of the data).

#### Time series breakpoint analysis

To explore changes in trends over time we carried out a breakpoint analysis. The Corpus of Historical American English (COHA) provides historical text samples across fiction, non-fiction, news and magazines categories. The type token ratio, word entropy and Zipf exponent were calculated for each text sample following the text processing steps outlined above.

For each media category and lexical measure, the results were binned into years and the median taken each year. The median was used to reduce the effect of outliers (similar results were found when using the mean). The medians were plotted on a scatterplot.

Visually, the scatterplots are suggestive of some change in the gradient of the lexical measure in time. In order to estimate the location of these breakpoints, we used Python’s piecewise-regression package^31^ with default settings. The regression fits and locations of breakpoints are shown in the scatterplots in Supplementary Fig. 8.

We ran a similar analysis with the categories combined. In order to combine the categories, we first took the means for each year and category and then took the mean across categories for each year. It is more natural to use means than medians when combining categories. The scatterplot and piecewise-regression fit for the combined word entropy is shown in Supplementary Fig. 9.

#### Time series trend analysis

For each category and lexical measure, trend analyses were carried out on the annual median values between the years 1900 and 2009 (the last year of data). Kwiatkowski–Phillips–Schmidt–Shi (KPSS) and Mann–Kendall (MK) tests were carried out for each measure and media category in COHA.

We carried out a total of 24 hypothesis tests for trends. To adjust for multiple tests, we applied a Holm–Bonferroni correction to adjust the *p*-values. The corrected *p*-values are reported.

The KPSS test assumes the null hypothesis of a stationary time series. *p*-values below 0.05 (adjusted following the Holm–Bonferroni correction) mean that we can reject this hypothesis at 5% significance and provide evidence of a trend. The test was applied using Python’s statsmodels package^32^. This is a one-sided test.

The KPSS test statistic follows a non-standard distribution, and in the statsmodels package *p*-values are calculated by comparing the test statistic against pre-calculated critical values up to *α* = 0.01 ^32,33^. In order to calculate *p*-values below *p* = 0.01 we used numerical methods through simulation of the test distribution.

The MK test is a non-parametric trend test^34^. The test assumes no serial correlation i.e., errors in one observation do not predict errors in other observations^34^. The text corpora are constructed from independent text samples so this is a reasonable assumption. The null hypothesis is that the data has no trend, and the *p*-value tells us the probability that the data was observed under the null hypothesis. At 5% significance, we reject the null hypothesis if *p* < 0.05 (adjusted following the Holm–Bonferroni correction). The test was carried out using Python’s pymannkendall package^34^. This is a two-sided test.

#### Time series visualisation

In the Results section we present the smoothed time series for COHA with the media categories combined as well as for each of the media categories separately. For each media category, data was only included where there were at least 5 observations within the smoothing average window. For the combined time series, for each year an average was taken across the annual means of the media categories (only including the media categories that had a mean value for that year).

The time series show confidence intervals, which were calculated as 1.96 times the standard error of the mean. For the combined categories, the standard error of the mean was computed based on the delta method,

4 $$S{E}_{\bar{X}}=\frac{\sqrt{\mathop{\sum }_{i = 1}^{m}S{E}_{i}^{2}}}{m}\,,$$

with *m* depending on how many media categories had values for the annual mean each year.

#### Differences between media categories

In the Results section we present distributions of the lexical measures within media categories in COCA, the BNC and COHA (restricted to 2000–2007 to avoid the effect of historical changes). To test for differences between the groups we carried out ANOVA tests across categories within each corpus separately for each of the lexical measures. At 5% significance, *p* < 0.05 provides evidence that the media categories are drawn from different underlying population distributions. The tests were carried out using python’s statsmodels package^32^. We also applied a Holm–Bonferroni correction to control for the family-wise type 1 error rate while carrying out multiple hypothesis tests.

For visualisation, the distributions of word entropy for each media category are shown as a kernel density estimate with the bandwidth determined by the Silverman rule^35^. The Silverman rule is a relatively simple, well-known and robust heuristic that performs well over mild assumptions (data is unimodal, approximately symmetric and not heavy-tailed)^36^. We also removed outliers that were more than 5 standard deviations from the mean in each case (less than 0.1% of the data).

#### US magazine circulation

In order to explore connections between word entropy and qualitative changes in media publishing, we explored a case study of the US magazine industry in Supplementary Note 1: Historical Analysis of US Magazine Publishing.

The data for magazine circulation numbers were those reported by the Audit Bureau of Circulation^37^. This data source does not track all US magazines, but does track well-known magazines. The data was plotted without further treatment in Supplementary Fig. 1.

### Ethics

We followed principles of fair use in relation to the large text corpora (BNC^24^, COHA^20^, COCA^22^). The corpora are constructed specifically for academic and non-commercial use, with small text samples presented in a way to minimise the effect on copyright holders. In relation to social media data, we followed the terms and conditions of the social media companies at the time of data collection. The study was not pre-registered.

### Reporting summary

Further information on research design is available in the Nature Portfolio Reporting Summary linked to this article.

## Results

### The rising entropy of American English

**Fig. 1:**
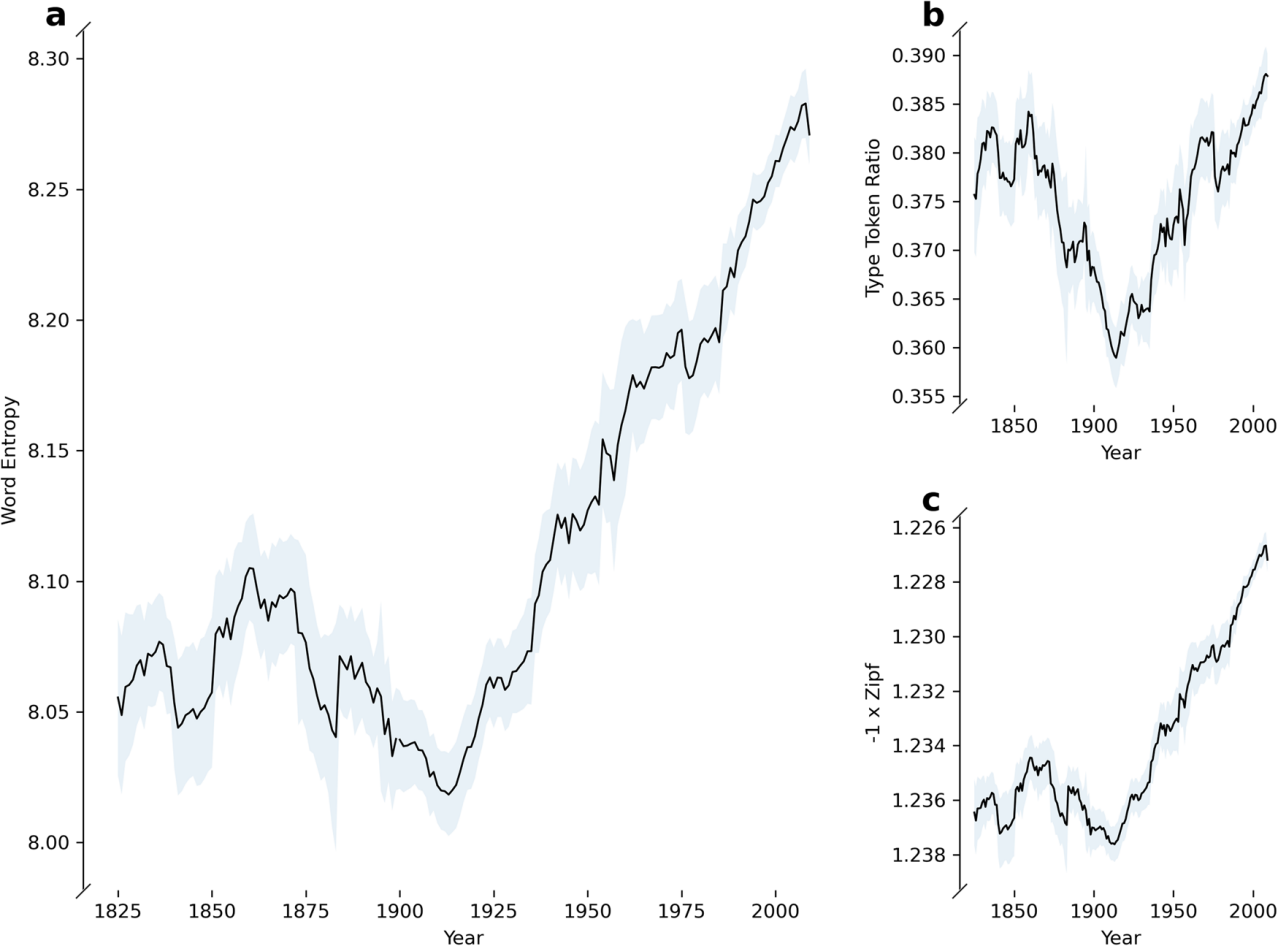
American English shows a trend of broadening word distributions since around 1900. Panels show timeseries of measures of (**a**) word entropy, (**b**) type token ratio, (**c**) Zipf exponent, in text samples from the Corpus of Historical American English (*n* = 22,233). Time series are smoothed with a moving average window of ±5 years, and averaged over media categories, with a 95% confidence interval in this average.

We analysed the Corpus of Historical American English (COHA), a balanced corpus with text samples from the 1810s to the 2000s categorised into news, magazines, fiction and non-fiction^20^. We found a clear trend of rising word entropy since approximately 1900, and more generally a broadening of word frequency distributions as measured by type token ratio and Zipf exponent (Fig. 1).

**Fig. 2:**
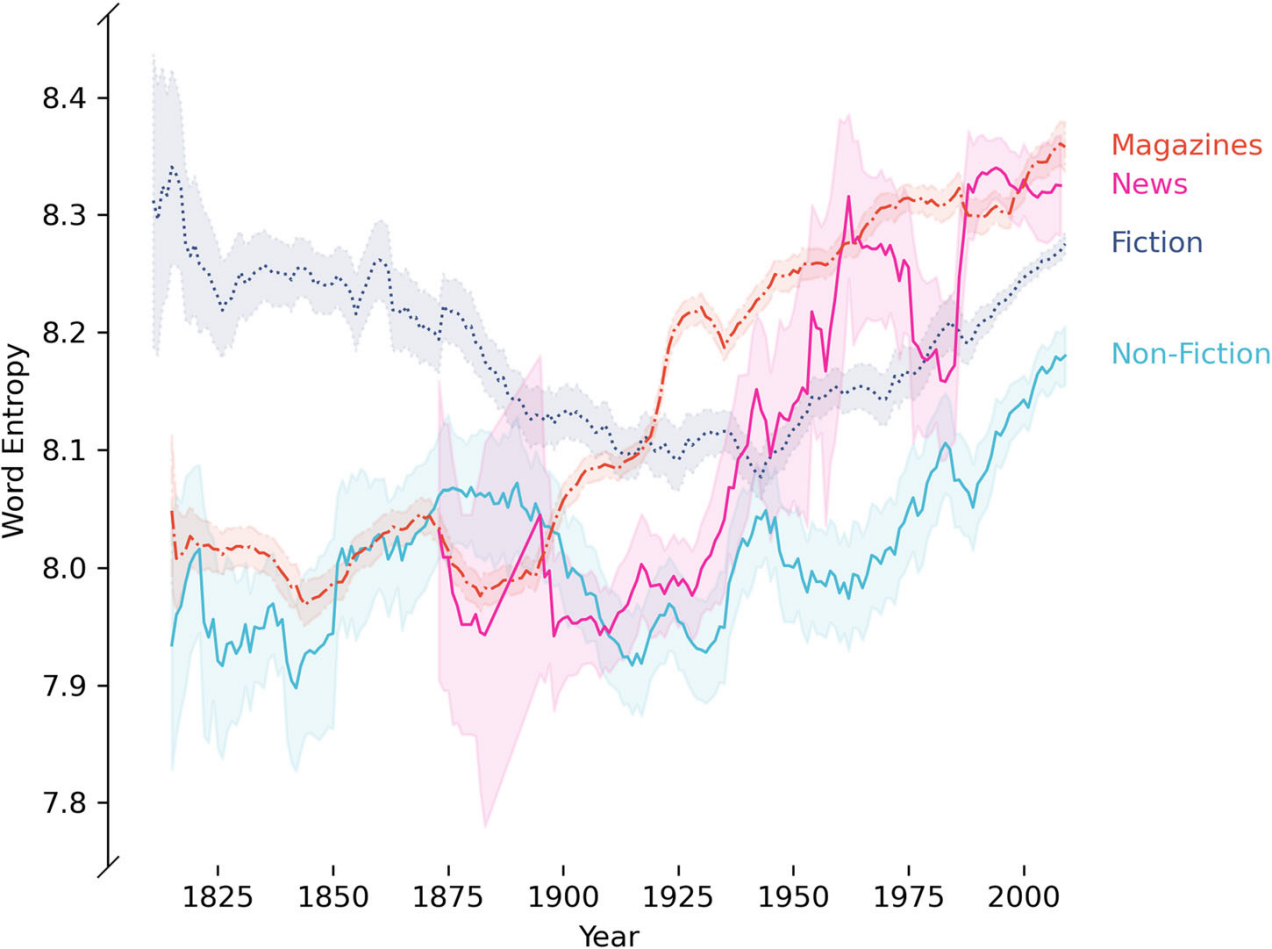
Trends of increasing word entropy across media categories since around 1900. Time series of word entropy in the Corpus of Historical American English across media categories of magazines (*n* = 11,306), news (*n* = 720), fiction (*n* = 8162) and non-fiction (*n* = 2045). For each media category, the time series was smoothed using an average over a window of ±5 years. The shaded regions are 95% confidence intervals of this average. All media categories show an upward trend in word entropy from 1900.

The trends in separate media categories follow the same pattern of rising word entropy (Fig. 2), again supported by similar patterns in type token ratio (Supplementary Fig. 2) and Zipf exponent (Supplementary Fig. 3). We analysed the time series of annual averages since 1900 for each media category (fiction, non-fiction, news, magazines) and lexical measure (word entropy, type token ratio, Zipf exponent) using Kwiatkowski–Phillips–Schmidt–Shin (KPSS) and Mann–Kendall (MK) tests on the annual median values (using the annual mean gives similar results). This gives a total of 4 × 3 × 2 = 24 trend tests. The results of these trend tests, reported in Table 1, represent strong evidence for the broadening of word distributions in all media categories between 1900 and 2010.

**Table 1:** Timeseries analysis across different categories and measures for text samples from COHA between 1900 and 2009

Measure	Category	KPSS	MK
Word Entropy	News	Statistic = 1.475, *p* < 0.001	Tau = 0.508, *p* < 0.001, 95% CI [0.374, 0.642], *N* = 98
Word Entropy	Magazine	Statistic = 1.741, *p* < 0.001	Tau = 0.712, *p* < 0.001, 95% CI [0.585, 0.840], *N* = 108
Word Entropy	Fiction	Statistic = 1.305, *p* < 0.001	Tau = 0.522, *p* = 0.001, 95% CI [0.395, 0.649], *N* = 109
Word Entropy	Non-Fiction	Statistic = 1.402, *p* = 0.003	Tau = 0.378, *p* = 0.005, 95% CI [0.251, 0.505], *N* = 109
Type Token Ratio	News	Statistic = 1.179, *p* < 0.001	Tau = 0.359, *p* < 0.001, 95% CI [0.225, 0.494], *N* = 98
Type Token Ratio	Magazine	Statistic = 1.066, *p* < 0.001	Tau = 0.409, *p* < 0.001, 95% CI [0.281, 0.536], *N* = 108
Type Token Ratio	Fiction	Statistic = 0.962, *p* = 0.002	Tau = 0.423, *p* = 0.002, 95% CI [0.296, 0.550], *N* = 109
Type Token Ratio	Non-Fiction	Statistic = 0.674, *p* = 0.007	Tau = 0.156, *p* = 0.010, 95% CI [0.029, 0.283], *N* = 109
Zipf Exponent	News	Statistic = 1.509, *p* < 0.001	Tau = −0.535, *p* < 0.001, 95% CI [−0.670, −0.401], *N* = 98
Zipf Exponent	Magazine	Statistic = 1.753, *p* < 0.001	Tau = −0.749, *p* < 0.001, 95% CI [−0.877, −0.622], *N* = 108
Zipf Exponent	Fiction	Statistic = 1.390, *p* = 0.002	Tau = −0.528, *p* = 0.003, 95% CI [−0.656, −0.401], *N* = 109
Zipf Exponent	Non-Fiction	Statistic = 1.297, *p* = 0.029	Tau = −0.402, *p* = 0.016, 95% CI [−0.529, −0.275], *N* = 109

Each row shows the result of a Kwiatkowski–Phillips–Schmidt–Shin (KPSS) test and a Mann–Kendall (MK) trend test. The *p*-values are corrected for multiple hypothesis testing with a Holm–Bonferroni correction. All of the hypothesis tests have *p*-values below 0.05, meaning that we can reject the null hypothesis of stationarity at 5% significance.

### Higher entropy in short-form media

**Fig. 3:**
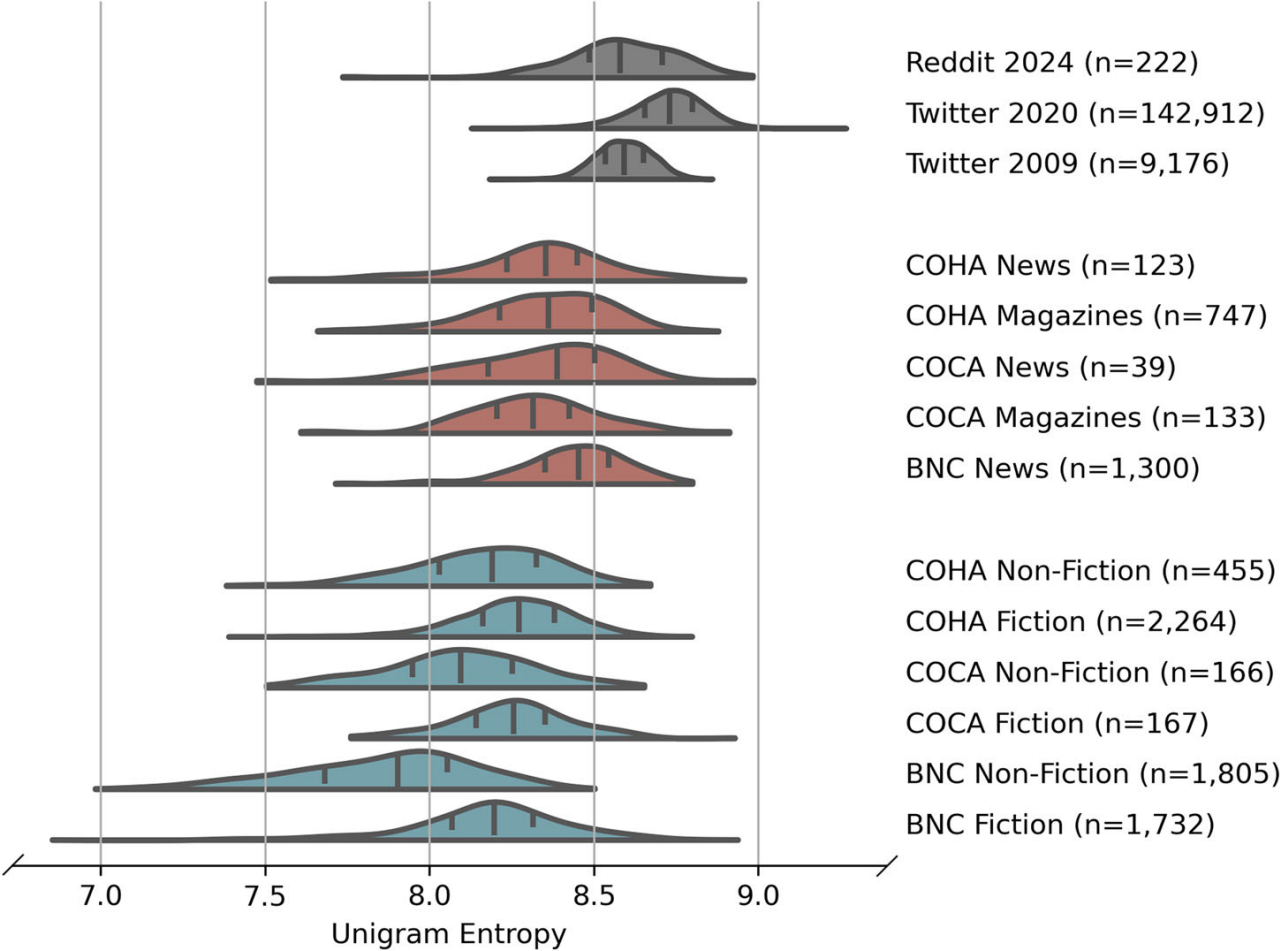
Short-form media has higher word entropy. Word entropy of very short-form (social) media, short-form (news and magazines) and long-form (fiction and non-fiction) media. For each media category, distributions are kernel density estimates cut to the data range, with quartile positions shown. The COHA data was restricted to 2000–2007 to minimise the effect of historical changes.

The historical trend (Fig. 2) suggests modern differences in entropy between media categories. However, we also know that short-form media have become especially prominent with the recent rise of online platforms for media distribution, such as social media, RSS feeds, and news platforms that present short headlines and snippets that link to long-form articles. To investigate these different media categories, we examined the Corpus of Contemporary American English (COCA) and the British National Corpus (BNC), as well as social media data from Twitter (now X) and Reddit. Figure 3 shows the distribution of word entropy across different media categories. Within COHA (limited to 2000–2007), BNC, and COCA there were significant differences in all lexical measures across media categories (see Table 2). Overall, the short-form media categories of news and magazines have higher entropy than long-form media, and social media feeds have the highest entropy of all. We see similar patterns in distributions of type token ratio (Supplementary Fig. 5) and Zipf exponent (Supplementary Fig. 6).

**Table 2:** Analysis of differences in word measures across media categories within each text corpus

Lexical Measure	Corpus	ANOVA Result
Word Entropy	COHA	F(3, 3586) = 83, *p* < 0.001, *η*^2^ = 0.065, 95% CI = [0.050, 0.086]
Word Entropy	COCA	F(3, 501) = 34, *p* < 0.001, *η*^2^ = 0.172, 95% CI = [0.121, 0.247]
Word Entropy	BNC	F(2, 1316) = 662, *p* < 0.001, *η*^2^ = 0.502, 95% CI = [0.469, 0.537]
Type Token Ratio	COHA	F(3, 3586) = 34, *p* < 0.001, *η*^2^ = 0.028, 95% CI = [0.019, 0.041]
Type Token Ratio	COCA	F(3, 501) = 17, *p* < 0.001, *η*^2^ = 0.094, 95% CI = [0.052, 0.157]
Type Token Ratio	BNC	F(2, 1316) = 416, *p* < 0.001, *η*^2^ = 0.388, 95% CI = [0.354, 0.426]
Zipf Exponent	COHA	F(3, 3586) = 89, *p* < 0.001, *η*^2^ = 0.070, 95% CI = [0.050, 0.096]
Zipf Exponent	COCA	F(3, 501) = 39, *p* < 0.001, *η*^2^ = 0.190, 95% CI = [0.138, 0.258]
Zipf Exponent	BNC	F(2, 1316) = 682, *p* < 0.001, *η*^2^ = 0.509, 95% CI = [0.472, 0.547]

One-way ANOVA tests are reported with the F-statistic (degrees of freedom), *p*-value, effect size (*η*^2^), and bootstrapped 95% confidence interval for the effect size. The reported *p*-values were adjusted for multiple testing with a Holm–Bonferroni correction. All tests find *p*-values below 0.001, indicating that we can reject the null hypothesis of no difference across categories at a 0.1% significance level.

Our analysis involved collating posts to simulate social media feeds. Combining posts will naturally lead to high entropy text, with fast switching of contexts and high novelty. This mirrors how people actually consume social media. Essentially, social media platforms generate high entropy information environments in the form of feeds of short messages from different users. This is not necessarily a linguistic change in how people generate English; it is a change in how people consume English text.

## Model

### Information foraging in the attention economy

The previous results are suggestive of a link between competition for attention and word entropy. To explain these results we generate a model of the attention economy based on information foraging. Foraging models relate the consumption of information items with some utility gain to the forager. To bridge utility rates to lexical measures, we borrow the idea of information signal entropy from Shannon^38^: The entropy of a source of information is a function of the probability of seeing each symbol. This function is equivalent to the expected reduction in uncertainty when receiving symbols, defined as a rate of information per symbol. While this is a static measure, it also reflects the potential utility gain per unit of time as foragers encounter and process information, with the assumption that an increase in entropy, *h*, is associated with an increase in utility rate, *r*. This aligns with Zipf’s principle of least effort^1,4^.

5 $$h\propto r\,.$$

Animal foragers modulate the selectivity of their diet in response to the environment, becoming more selective in times of abundance^39^. Why waste energy hunting difficult prey when there are plenty of easy calories around? Humans act in the same way when selecting information to consume^6,10^. We have all experienced situations where we do not have access to the internet, for example on a plane or train journey, and we become less selective in what we read or watch.

This characterisation of attention corresponds to the prey choice model, which describes which types of prey are worth pursuing and consuming^39^. This has been applied to information foraging before^10^. The prey model that we describe and extend here is analogous to that found in the prey choice model in the animal foraging literature^39^.

Assume an information forager searches a media environment and encounters information of types, *i*, at Poisson rates *λ*_*i*_. If consumed, information provides a benefit *u*_*i*_ in a handling time *t*_*i*_, during which time the forager is not searching. Alternatively, the forager can choose to ignore information of a certain type and keep searching. The forager’s choices to consume or ignore information determine the expected total time spent searching, *T*_*s*_, and handling, *T*_*h*_, information, as well as the total utility gain, *U*. Given these constraints, the forager aims to optimise the expected overall rate of utility of foraging given by

6 $${R}_{media}=\frac{U}{{T}_{s}+{T}_{h}}\,.$$

Here *media* describes the forager’s local environment, such as a media platform. Media platforms are analogous to foraging patches in optimal foraging theory. The forager’s choices of which information types to consume can be described as an information diet, *D*. The total expected utility is *U* = ∑_*D*_*λ*_*i*_*u*_*i*_*T*_*s*_. Similarly, the total expected handling time is *T*_*h*_ = ∑_*D*_*λ*_*i*_*t*_*i*_*T*_*s*_. Substituting in and cancelling *T*_*s*_, we can write the expected utility rate given a diet

7 $${R}_{media}=\frac{{\sum }_{D}{\lambda }_{i}{u}_{i}}{1+{\sum }_{D}{\lambda }_{i}{t}_{i}}\,.$$

Consuming an information item carries an expected opportunity cost of not spending that item’s handling time looking for other items, equal to *t*_*i*_*R*_*media*_, and an expected utility gain of *u*_*i*_. To maximise expected utility rate a forager should therefore consume the item if the item utility rate, ${r}_{i}=\frac{{u}_{i}}{{t}_{i}}$, is greater than the overall media platform utility rate, *R*_*media*_,

8 $${r}_{i}\ge {R}_{media}\,.$$

This diet threshold condition is a familiar result from foraging theory^10,39,40^. To find the optimal diet, item types can be ranked in order of *r*_*i*_ and added to the diet one by one until this inequality fails^40^. See the Supplementary Note 2: Prey Choice Model Derivation for a more thorough derivation.

We can now ask which information types a forager should include in their diet, *D*, to maximise their expected overall utility rate as a consequence of rising information prevalence, here *λ*_*i*_. For items with *r*_*i*_ < *R*_*media*_, increasing prevalence has no effect as these items are still not included in the diet. For items with *r*_*i*_ ≥ *R*_*media*_, increasing prevalence will mean more time spent handling these items and less time spent searching, so the overall media platform utility rate will increase,

9 $$\frac{\partial {R}_{media}}{\partial {\lambda }_{i}}\ge 0\quad \forall i\,.$$

Combining this with the information diet criterion (Inequality 8), we reach a general conclusion. *Increasing information prevalence increases the information utility rate required for inclusion in the diet: foragers become more selective when prey (or information) becomes more abundant*, analogous to the prey model in optimal foraging theory^39^.

**Fig. 4:**
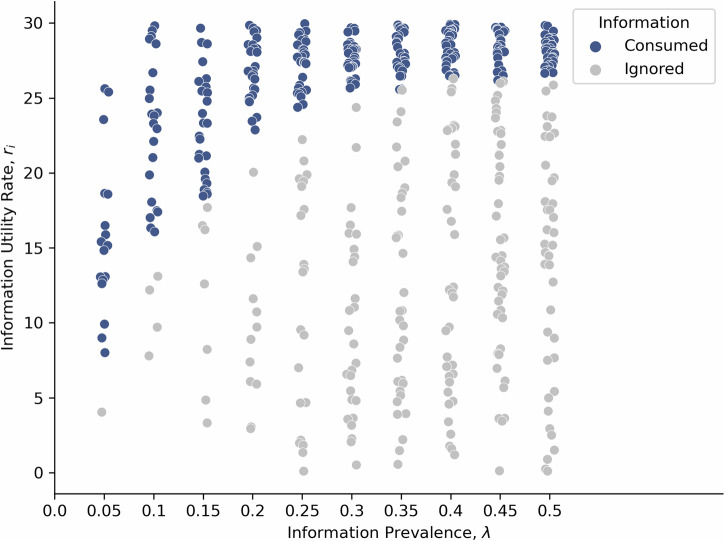
Simulation of information foraging in the attention economy. Information items are generated with random utility rates in quantities proportional to the information prevalence. Given the information environment, foragers only consume information items above a minimum information density (blue markers) in order to maximise their foraging rate. Information that is not consumed has less chance of survival (grey markers). Overall the surviving information types have higher utility rates at higher information prevalence.

We now extend traditional foraging theory to information co-evolution by asking how media producers respond to increasing selectivity among information foragers. By assuming there is some cost to media of producing more informative messages—a standard assumption underlying Zipf’s principle of least effort^1,41^—we conclude that an abundance of information creates an adaptive pressure that drives media producers to create information with a higher utility rate. A proxy for utility rate is information density, or word entropy. Figure 4 shows a simple simulation of this dynamic.

### Competition between media platforms drives differences between short- and long-form media

Information is distributed in media platforms (e.g., newspapers, magazines, books, Twitter, Reddit). The forager has to choose not only which information to consume within a media platform, but also which media platforms to visit. Analogous to the information choice model (Equation (8)): an optimal information forager will visit a media platform if the expected media utility rate is greater than the background utility rate from foraging in the overall environment (see Supplementary Note 3: Patch Choice Model and Non Constant Patches for the full model),

10 $${R}_{media}\ge {R}_{env}\,.$$

The utility rate of a media platform, *R*_*media*_, is a summation over Poisson processes (Equation (7)). To simplify this, let ${\bar{u}}_{m}$ be the average utility of information items consumed in the media platform, ${\bar{t}}_{m}$ the average time spent consuming information items, and *λ*_*m*_ the rate of encounter of any item in the diet. Equation (7) then becomes a variation of Holling’s disc equation^42^ (full derivation in Supplementary Note 4: The Merged Poisson Process for Patches).

11 $${R}_{media}=\frac{{\lambda }_{m}{\bar{u}}_{m}}{1+{\lambda }_{m}{\bar{t}}_{m}}\,.$$

**Fig. 5:**
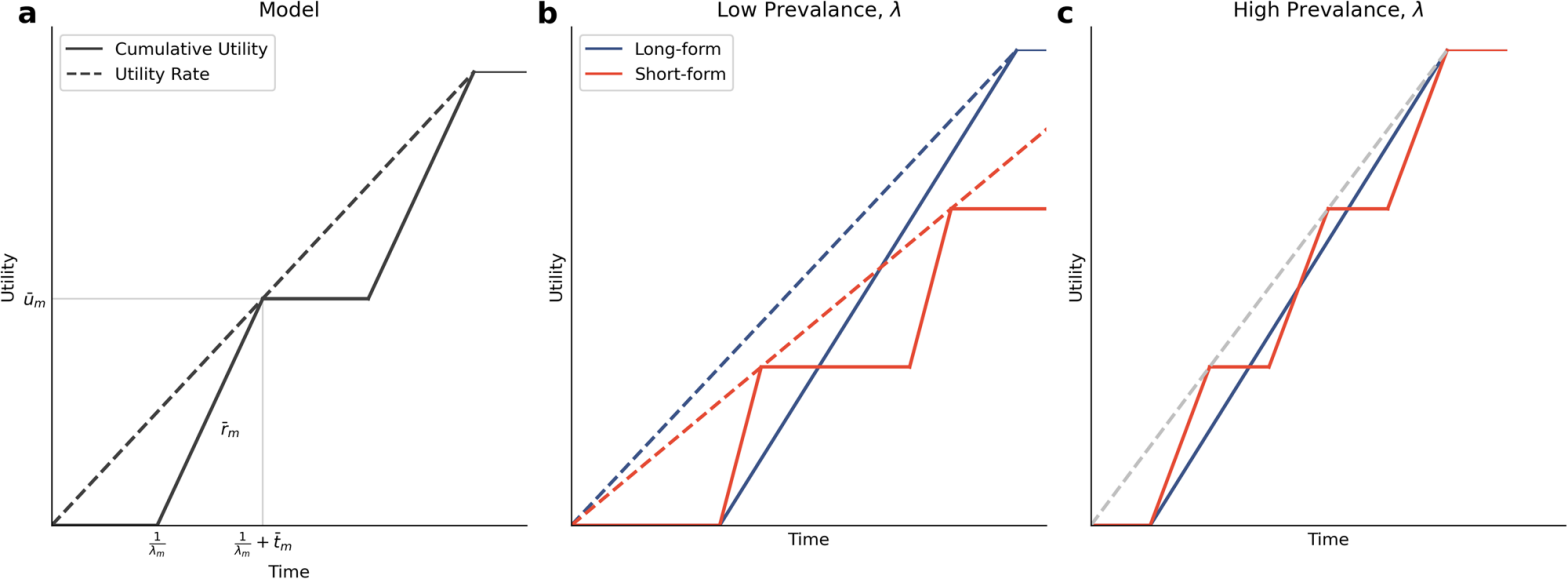
The media patch model. **a** The expected utility rate of a media patch (dashed line) is determined by the time spent searching for (horizontal solid line) and consuming (diagonal solid line) information items. **b** In a low prevalence environment long-form media has an advantage, although at low prevalence foragers are not very selective. **c** At high prevalence less time is spent searching between item acquisition. To reach the same overall patch utility rate (dotted grey line), short-form media needs a higher information utility rate (gradient of the solid diagonal red line) than long-form media (gradient of the solid diagonal blue line).

This equation is visualised in Fig. 5a.

The criteria for inclusion in an information forager’s diet is then

12 $$\frac{1}{{\lambda }_{m}{\bar{u}}_{m}}+\frac{1}{{\bar{r}}_{m}}\le \frac{1}{{R}_{env}}\,.$$

The inclusion of a media platform in the information diet is therefore determined by three properties: the average utility (i.e., size) of a item, ${\bar{u}}_{m}$; the average item utility rate, ${\bar{r}}_{m}$; and the prevalence of items within the media platform, *λ*_*m*_.

Short-form media platforms such as news and magazines involve more time spent switching (and searching for) articles than long-form media platforms such as books. In order to reach the same overall media platform utility rate, *R*_*media*_, short form media types need to have higher information utility rates (Fig. 5c). This creates a differential selective pressure on short- and long-form media producers. Given some *R*_*env*_, the short-form media platform needs higher average information utility rates, ${\bar{r}}_{m}$, to be accepted in the forager’s diet than the long-form media. The long-form media experiences a relaxed selective pressure on information utility rates because there is less time spent switching in these media platforms. This can describe the differences in the observed information utility rates in short- and long-form media as well as the trend towards increased information rates with increasing media prevalence.

### Social media

**Fig. 6:**
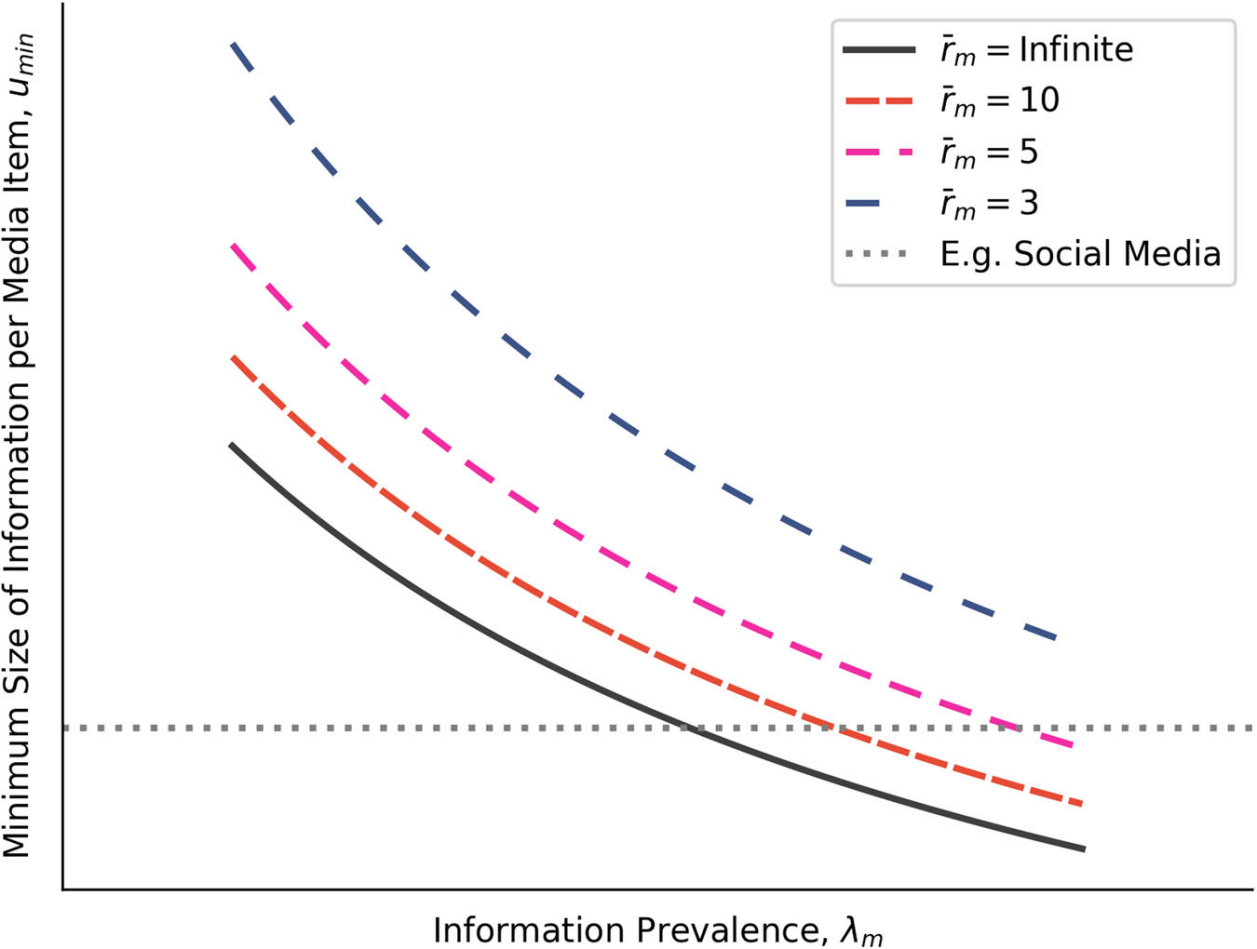
Short-form media becomes more viable when information prevalence is high. Minimum average information size, *u*_*min*_, for media platform diet inclusion for varying levels of information prevalence, *λ*_*m*_. Increasing average information utility rates, ${\bar{r}}_{m}$, can increase this limit only to a point. Very short-form media platforms like social media can only capture attention in a world with high information prevalence.

Inequality (12) includes a weaker condition for diet inclusion, $\frac{1}{{\lambda }_{m}{\bar{u}}_{m}}\le \frac{1}{{R}_{env}}$. This indicates that information prevalence directly limits the minimal average size of information for diet inclusion. As information prevalence increases, foragers will tolerate media platforms with smaller and smaller information item sizes (Fig. 6). More intuitively, Twitter only works in a world with instant messages—few people would go to a library to read a single Tweet.

Finally, our model quantifies the selective forces acting to make media platforms more accessible. If a media platform reduces the expected search time between information encounters, $\frac{1}{{\lambda }_{m}}$, then they reduce the left hand side of Inequality (12) and become more competitive. This asymmetrically affects utility for short-form media, $\frac{1}{{\lambda }_{m}{\bar{u}}_{m}}$; for long-form media this term is already small. This could be an explanation for innovations towards minimising time spent searching in short-form media platforms such as infinite scroll and autoplay videos.

## Discussion

We provide evidence that the word entropy of American English has increased over the 20th century. Furthermore, this change is marked by differences across different media categories, with the highest entropy levels found in the shortest media forms. Using a model of the attention economy based on information foraging, we show how a simple model of information selection can drive the observed changes. The attention economy model explains two results: a rise in entropy as information becomes more abundant and a rise in preferences for information dense short-form media.

Our model connects changes in language to social behaviour. Previous work along these lines has shown language evolution to follow a number of principles governed by human psychology. These principles have, for example, included features of biological and cultural evolution^43,44^, learning^44–46^, word formation and distribution^41,47,48^, and the decay of morphological complexity^46,49^. We add to this list and claim that word entropy rises in response to information abundance.

Our findings offer an interesting additional perspective on pressures involved in linguistic evolution. For example, the Linguistic Niche Hypothesis^46^ predicts a loss of complex morphological forms in English due to the influence of second language learners. Indeed, there is ample evidence that English is undergoing morphological simplification^49–51^. If we can summarise the effect of the Linguistic Niche Hypothesis as a pressure towards learnability, then our study claims an additional pressure towards expressivity. One might expect these two dynamics to be in opposition, as simpler symbolic rules would a priori be less expressive per symbol and therefore have lower entropy^52^. However, a more nuanced analysis can take into account the fact that human language includes redundancy^53^, in which case a pressure towards learnability through simplification by reduction of this redundancy would simultaneously increase entropy. While we can speculate about the interaction of these pressures (and other factors), we encourage further work in this area.

An unexpected consequence of our model is that very short-form media becomes viable when people are able to switch to new pieces of information quickly, with a relationship between minimum average information size and information prevalence (Fig. 6). This is framed in our model as increasing the platform information density by reducing information search costs and simultaneously increasing entropy through rapid switching of contexts between short information items. Social media further amplifies the relevance of this information by using algorithms that serve highly personalised feeds^54–57^, potentially interacting with feelings of social connection and shared reality^58^. This in turn has an effect on what media is produced, with creators and influencers tailoring their content to get picked up by the algorithms^54^.

Considering people as information foragers, our model describes observed empirical changes in word entropy of English over time and both within and between media categories in response to a reduction in search costs. Empirical findings support the idea that people’s attention is attracted to high entropy and high complexity information^59,60^. Our analysis of historical data shows the entropy of information markets respond predictably to increased competition. The attention economy model offers a simple explanation: humans are, within limits, information rate maximisers responding to rising information abundance and media producers adapt their content to compete for more limited attention.

### Limitations

The model presented here is a simplification of the real-world dynamics, which include complex interactions between human behaviour, economics, technology, and culture. We highlight some of these limitations here.

When using big data to analyse culture, a key question to ask is whether we are detecting variation in the underlying population of interest (in our case media expression of language) or variation in the data source (in our case text corpora). While this is an inherent problem when studying changes in language, we took steps to ameloriate the issue. This included the choice of COHA, which is a balanced corpus, with representative text samples across media categories. In contrast, we investigated but did not choose to use Google Ngrams due to question marks surrounding variation in the corpus composition over time^61^. Given the use of COHA, there was a challenge in balancing the benefits of large text samples with the number of text samples available. We found that a target size of *N* = 2000 words gives a good balance between sample length and quantity of samples. We also added robustness checks when comparing text samples across media categories by using multiple corpora. More generally, we note that big data corpus analysis is not a replacement for traditional cultural sociology techniques, but a complement^62^ and we encourage future work along more traditional qualitative lines^63^.

Our findings are primarily concerned with differences in word entropy, which is a measure on word distributions. To give a fuller account of these changes, we also analysed the type token ratio and Zipf exponent. It should be noted that these are unidimensional measures of word frequency distributions and do not capture all of the information of those distributions. As such, these measures can diverge significantly in artificially generated text samples^64^. At the other extreme, if word distributions are tightly constrained (e.g., by being perfectly Zipfian) then the measures are all statistically sufficient in that they capture the full information of the distribution (and correlate perfectly with each other). In empirical samples of natural languages these measures correlate well (although not perfectly), and have been grouped together as a measure of “lexical diversity”^2^. Overall we feel that including analyses of the word entropy, type token ratio and Zipf exponent give a rounded picture of changes in word distributions.

The text measures used are all sensitive to sample size. We investigated a process of extrapolating the measures to allow comparisons across varying sample sizes. For example, unigram word entropy measures converge at about *N* = 50,000 tokens and have relatively stable convergence curves as a function of number of tokens^65^. And Zipf estimators have systematic positive biases as functions of the exponent and sample size^66^. However, this kind of extrapolation would bring in extra assumptions that may not apply universally across time or text categories. To control for sample size sensitivity we therefore chose to truncate text samples to *N* = 2000 words, which is appropriate as we are interested in comparisons rather than absolute estimations.

The measurement of entropy from a finite sequence is a difficult problem, with many approaches of varying degrees of complexity. For example, Lempel-Ziv compression ratios converge to entropy rates with infinite sequences produced from an ergodic source^67^. However, language is not ergodic and we do not have infinite sequences. We decided to use the simplest and most well known form of entropy estimation with the maximum likelihood or plug-in estimator to find empirical unigram entropy. Our choice is supported by work that shows that this measure correlates well with more advanced estimators^65^. We extended the analysis to bigram entropy using the maximum likelihood estimator, with broadly similar timeseries trends (Supplementary Fig. 4) and differences across media categories (Supplementary Fig. 7). We considered analysing the conditional entropy, but were concerned that this involves the combination of estimates of both unigram and bigram probabilities, which have varying potential degrees of bias as a function of sample size and the underlying distribution. Estimates of conditional entropy therefore include a mix of variation in (a) the underlying distribution as well as (b) unigram probability biases and (c) bigram probability biases. We are not able to control for these biases and as such we feel that this is not a suitable form of analysis. We considered using a Kneser–Ney language model^68^ to overcome this issue but we decided that any accuracy benefits were outweighed by the obfuscation created by this more complicated model. We support future work that investigates other measures of entropy.

In the Supplementary Note 1: Historical Analysis of US Magazine Publishing, we more closely investigated the US magazine industry, finding that changes in word entropy tend to follow changes in the magazine industry towards increasing competition for attention. As a part of this analysis, we compared increasing word entropy to magazine circulation as measured by the Audit Bureau of Circulation, which was created in 1914 specifically to verify magazine circulation data for the purposes of advertisements^69^. There are some limitations to this data source as it does not track all magazine sales, only the most popular magazines^37^, i.e., the data is not a precise measure of all magazine circulation. However, the overall trend of increasing magazine circulation is well supported by other historical analyses^69^.

The attention economy is a well established concept that recognises that information producers must compete for attention^6,70^. This apparently simple mechanism obscures the vast machinery of modern media to capture attention as quantified by clicks and likes^8^. In order to decide what content to serve, user behaviour data is fed into optimisation algorithms that range from A/B testing^71^ to machine learning models^55^. To add to the complexity, salient topics rise and fall as the news cycle progresses. And there is evidence that this cycle itself is speeding up with shortening collective attention spans linked to attention economy pressures^9^. In addition, an abundance of information can lead to *information overload* where individuals are unable to efficiently use the information available to them^72^. Overall the attention economy is a complex and multi-faceted system. The full intricacies of this system are beyond the scope of this study, and we urge caution when interpreting and generalising the results that we present here. We hope that our study inspires further research to investigate the interactions between media and human behaviour.

Our study is limited to analysing text. Media is not limited in the same way and competition for human attention is played out across a multimedia landscape. There are well established differences in the way that people process information across modalities^73–75^, which suggest partially independent attention and processing mechanisms for audio and visual information^76–78^. Notably, multimedia information (e.g., text and images) can in some circumstances be processed more effectively than when presented in a single modality^73,76,79^. The idea of multimedia competition for attention is particularly relevant to modern media with rapidly growing audiovisual platforms such as TikTok^54^. While focusing solely on text is limited, it does provide a useful framework for historical comparison, as well as the availability of well established data, tools and methodologies for analysis. Beyond text we also see potential trends of increasing entropy in video with historical declines in shot length (time between the camera switching) [ref. ^80^, Chapter 11] in film^81^ and television^82^.

The attention economy also seems to be changing *how* people engage with information. A review of studies between 2004 and 2021 found shortening attention spans, in terms of time spent before switching media [ref. ^80^, Chapter 4]. This may be due to behavioural adaptations to efficiently process information in a media-rich world, or it may represent fundamental changes to cognitive systems. There are suggestions that screen media use in childhood can influence development towards lower attention spans and faster switching between media^83^, as well as rates of attention deficit hyperactivity disorder^83,84^. However, the nature of any associations, and their causality, is debated^85^. We hope that our model can contribute to this important debate.

We explore the dynamics of the attention economy through the use of information foraging models that are analogues of the diet choice model from food foraging. The wide applicability of food foraging models across varied taxa^39^ points to their generality. This generality can be understood in the context of the recurring and shared evolutionary challenge of the search for food. In applying these models to information foraging in humans^10^, the claim is that this generality extends even beyond food and to the realm of information, which is a similar search problem^10,11^, involving similar brain mechanisms^17,18^, and with similar observed empirical behaviour^10,86,87^. While this argument is convincing, we recognise that human behaviour is in general more complex than animal behaviour, with more complicated goals and strategies. In addition, individual heterogeneity in e.g., values and motivations, can interact in complex ways with how people produce^88^ and consume^89^ information. Humans also learn and are influenced by culture, which is particularly relevant in the realm of information search. In light of this, while information foraging models can be useful, we recognise the limitation of using such simple models to describe human behaviour. Future studies could explore the influence of human biases, cultural norms and learning, as well as heterogeneity in human behaviour associated with e.g., political beliefs, education, gender, and socio-economic background. A clear step would be expanding these research questions to languages beyond English.

Beyond information density^59,60^, there is evidence that human attention is also attracted to information that is belief-consistent^5,90,91^, predictive^5^, social^5,92^, negative^5,92,93^ and emotive^94–96^. Our model does not account for these other dimensions, and instead we make the assumption that aggregate behaviour can be usefully understood by considering the dimension of information density in isolation. A similar assumption is made in food foraging models, which usually focus on net calorie intake^39^ while omitting other important factors such as macronutrient content^97^ and predation risk^98^. However, just as food foraging models have their limitations in capturing the multi-faceted nature of foraging, so does our model in capturing the intricacies of information search. Future work could explore how various factors, including word entropy, attract human attention. This could extend to considering how different factors might interact. For example, the extremely attention grabbing utterance “FIRE!” is at once concise, predictive, negative and emotive.

There is much further work needed to understand how people choose what information to consume, and how that affects their behaviour. Modern technology creates opportunities for new experiments. While eye-tracking experiments^59,60,99^ are well established, we can also characterise and measure attention in terms of cognitive mechanisms^58,94,100^. Beyond attention, we can ask how beliefs are influenced through factors such as emotive^101^ or narrative^102^ descriptions. An approach that is aligned with attention economy questions is to measure behavioural engagement while carrying out tasks, either in researcher-designed websites^103^ or in naturalistic settings [ref. ^80^, Chapter 4]. Beyond experiments, the use of up-to-date real-world data from prominent social media sites represents a significant opportunity for insights into what drives engagement. Such datasets are undoubtedly rich and offer many avenues for analysis including mixed methods approaches. There is sometimes a challenge in terms of access to such datasets, as such data can offer a competitive advantage to private social networks to improve engagement through their own private research efforts.

### Conclusion

We ask how the entropy of American English has changed over time, and further investigate differences in word entropy across media categories. We analyse an ecological model of information foraging in the attention economy that aims to explain the observed trends as a market response to increased competition for human attention.

### Supplementary information

Peer Review File

Supplementary Material

Reporting Summary

## Data Availability

All data generated following analysis of text samples is available at https://github.com/chasmani/PUBLIC-rising-entropy-of-english. The text corpora data is not included in the public repository for copyright and size reasons. They are available: • COHA^21^ and COCA^23^ (https://www.corpusdata.org/). • BNC^24^ (http://www.natcorp.ox.ac.uk/). • Twitter 2009 dataset^28^ (https://www.kaggle.com/kazanova/sentiment140). • Twitter 2020 dataset. This was collected from Twitter’s API between February 2020 and January 2021. We cannot provide the data for copyright reasons. • Reddit dataset. This was collected from Reddit’s API between 5th to 17th January 2024. We cannot provide the data for copyright reasons (https://www.reddit.com/.json).

## Code availability

All code used to generate figures and analysis is available at the GitHub repository https://github.com/chasmani/PUBLIC-rising-entropy-of-english^104^. We used python (version 3.9.0) and the following packages and versions (beautifulsoup4==4.12.2, nltk==3.7, pandas==1.5.3, scipy==1.10.1, seaborn==0.12.2). We used code from project Gutenberg https://github.com/c-w/gutenberg/(version 0.8.2). All code used to collect data is available at the GitHub repository https://github.com/chasmani/PUBLIC-rising-entropy-of-english^104^. For API access, we used Python (version 3.9.0) and the requests library (version 2.28.0).
