# Supplementary material for: The rising entropy of English in the attention economy
Source: Commun Psychol. 2024 Aug 1;2:70. doi: 10.1038/s44271-024-00117-1 (PMC11332035; doi:10.1038/s44271-024-00117-1)
Supplement: Supplementary file 2 — Supplementary Material [file 44271_2024_117_MOESM2_ESM.pdf]

The Rising Entropy of English in the Attention Economy - Supplementary Information

Charlie Pilgrim[1,2,3,4,*] Weisi Guo[4,5] Thomas T. Hills[4,6]

[1]Mathematics, University of Leeds, Leeds, UK
[2]The Mathematics of Real-World Systems CDT, The University of Warwick, Coventry, UK
[3]Experimental Psychology, University College London, London, UK
[4]The Alan Turing Institute, London, UK
[5]Human Machine Intelligence Group, Cranfield University, Bedford, UK
[6]Department of Psychology, The University of Warwick, Coventry, UK
[*]Corresponding author: Charlie Pilgrim, c.p.pilgrim@leeds.ac.uk

Supplementary Note 1: Historical Analysis of US Magazine Publishing

As a case study we investigated the history of magazine publishing in America. Figure 1 shows the historical trend in COHA magazine word entropy alongside magazine circulation figures and important events. Magazine publishers are in a two-sided market where they sell magazines to consumers and attention to advertisers [1], with the majority of revenue from selling attention [2]. This wasn't always the case in the US — prior to the 1890s most magazine revenue was from sales, with advertising considered undesirable [2]. Towards the late 19th century a combination of rapidly decreasing printing costs, growth in the literate population, discounts from the US postal service and the ability to target adverts to a niche readership led to a new business model to emerge [2]. This new model involved selling magazines lower than the price of production, which increased circulation so that those costs could be recouped by advertising revenue [2]. Before 1893, most magazines sold for 25 cents — until a price war led to the magazines McClure's, Munsey's and Cosmopolitan dropping their prices to 10 cents and subsequently enjoying rises in circulation and advertising revenue [2]. The 10 cent magazines contributed to a tripling in total magazine readership from 1890 in 1905 [2], and there was a huge jump in word entropy in the same period (Supplementary Figure 1).

The Audit Bureau of Circulation was created by advertisers in 1914 [2] to more accurately measure magazine readership numbers. This quantification of attention further increased pressure on magazine publishers to improve their circulation numbers in order to sell advertising. Other changes included moving advertisements from the back of the magazine to alongside the main content — a move that forced copywriters to improve the appeal of the content through adding color and improving graphics [2].

Word entropy continues to rise throughout the 20th century alongside magazine circulation, with a Pearson's correlation coefficient r= 0.91 ($p < 0.001$), although both rise over time so that confounding factors are not ruled out (Supplementary Figure 1). We explored further with a Generalized Linear Model with an AR(1) component to account for autocorrelation in the data, finding that magazine circulation was a significant predictor of word entropy in this model (coefficient = 0.0004, $p < 0.001$). However, we did not find a significant correlation when the time series was detrended by analysing associations in the differences in magazine circulation and word entropy over 5 year periods, (Pearson's correlation coefficient $r = -0.094$; $p = 0.72$).

After the 1890s, the biggest drop in word entropy was during the great depression when magazine circulation also fell. There is a suggestion in the data that things change around the year 2000, as magazine circulation drops but word entropy continues to rise. The rise of digital media around this time is perhaps the biggest change in publishing since the printing press so we would not expect the same trends to necessarily continue — and digital media represents a new competitive pressure.

Draft

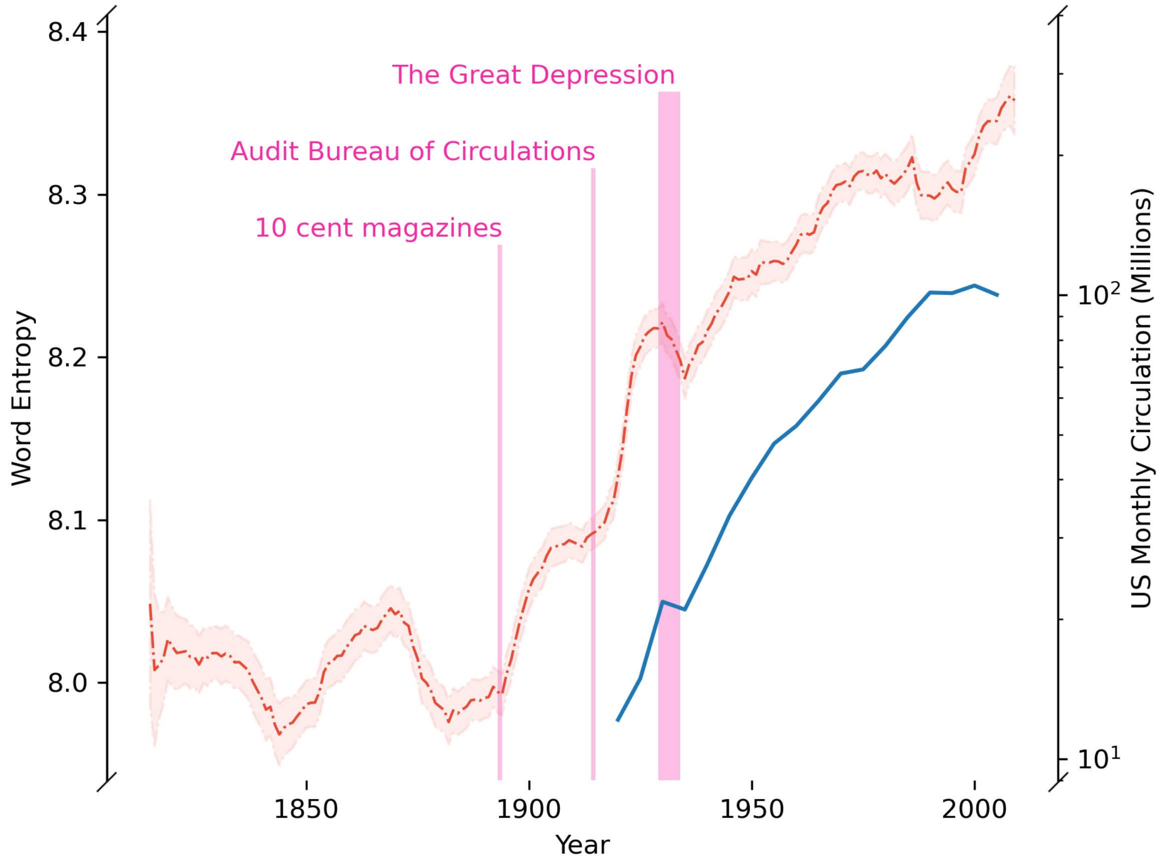

Supplementary Figure 1: Word entropy in American magazines increased with circulation. Historical analysis of word entropy in magazines (red dotted, timeseries calculated as in previous figure) with key events (pink) and US Monthly Magazine circulation as reported by the Audit Bureau of Circulations (purple).

Supplementary Note 2: Prey Choice Model Derivation

In the main paper we justify the prey choice algorithm using an argument that considers the opportunity cost of spending time handling a prey versus searching in the environment. Here we derive the same result more rigorously. This is a completely analogous derivation as found in optimal foraging theory [3]. As in the main paper, we have information types, i, that are encountered with rates λ_i while searching. Each information item, if consumed, provides a benefit u_i in a handling time t_i, during which the forager is not searching for other items.

In the main text, a media patch expected utility rate is given by,

$$R_{media} = \frac{\sum_D \lambda_i u_i}{1 + \sum_D \lambda_i t_i} \, . \tag{1}$$

This assumes that information types are either in the diet, D, in which case they are always consumed upon encounter, or alternatively the items are not in the diet and never consumed. We can generalise this so that forager's have some probability of consuming an information type upon encounter, p_i,

$$R_{media} = \frac{\sum \lambda_i u_i p_i}{1 + \sum \lambda_i t_i p_i} \, . \tag{2}$$

The forager can choose the probability of paying attention to each information type, and a forager's strategy can be defined as a vector $\mathbf{p} = [p_1, p_2, ..., p_n]$. These choices are independent. To find the strategy that gives the maximum utility rate we can consider each of these choices, p_j, independently. To find the best strategy we separate p_j from the summations and differentiate

$$\frac{\partial R_{media}}{\partial p_j} = \frac{\lambda_j u_j (1 + p_j \lambda_j t_j + \sum_{i \neq j} p_i \lambda_i t_i) - \lambda_j t_j (p_j \lambda_j u_j + \sum_{i \neq j} p_i \lambda_i u_i)}{(1 + p_j \lambda_j t_j + \sum_{i \neq j} p_i \lambda_i t_i)^2} \,. \tag{3}$$

Cancelling like terms

$$\frac{\partial R_{media}}{\partial p_j} = \frac{\lambda_j u_j (1 + \sum_{i \neq j} p_i \lambda_i t_i) - \lambda_j t_j (\sum_{i \neq j} p_i \lambda_i u_i)}{(1 + p_j \lambda_j t_j + \sum_{i \neq j} p_i \lambda_i t_i)^2} \,. \tag{4}$$

The sign of this does not depend on p_j. So if $\frac{\partial R}{\partial p_j} > 0$, R_{media} will be maximised with $p_j = 1$, and otherwise with $p_j = 0$. The condition for $p_j = 1$ is

$$\frac{u_j}{t_j} > \frac{\sum_{i \neq j} p_i \lambda_i u_i}{1 + \sum_{i \neq j} p_i \lambda_i t_i)} \,. \tag{5}$$

The right hand side is the total expected rate of utility for all items except for item j, $R_{\neg j}$. The item should be included in the diet if the utility rate of the item, $r_i = \frac{u_j}{t_j}$, is greater than the overall rate of foraging without the item.

$$r_j \geq R_{\neg j} \,. \tag{6}$$

This is equivalent to the diet inclusion criteria given in the main paper. To find the optimal diet, one can add items in order of their utility rate until the inequality fails.

Supplementary Note 3: Patch Choice Model and Non Constant Patches

The patch choice model considered in the main paper is analogous to the information choice model. Patches of each type are randomly encountered in the environment and encountered as a Poisson processes with rates λ_{media}. We also assume that patches have a constant expected rate of utility, R_{media}, and some finite time, T_{media} until the rate drops to zero, which gives each patch a total utility, U_{media}. Foragers can choose to either consume or ignore a patch upon encountering it. This model is identical to the information choice model so that we can follow that derivation and jump to the conclusion that a patch will be included in the diet if the patch utility rate is greater than or equal to the overall rate of foraging in the environment, $R_{media} \geq R_{env}$.

Information patches in the real world have non-constant utility rates. Commonly patch marginal utility will decrease with time [3, 4]. This can happen as finite prey are consumed [5, 3]. For example, within a patch an optimal forager will consume the most profitable items first if they can, which then makes those items more scarce and reduces the overall utility rate in the patch as time goes on [5]. Examples are collecting raspberries from a bush, or checking your email. Information items themselves may degrade while being consumed, for example news articles often follow an inverted pyramid structure where the most important information is presented first, with extra paragraphs adding marginally diminishing extra information [6]. Magazines, fiction and non-fiction have their own styles and utility curves. Overall we can say that utility rates in patches, and information, are not constant.

An optimal forager now has to choose both which patches to consume and how long to spend in those patches. This problem was solved by Charnov's marginal value theorem [4], which we derive here in the context of information items. We follow the model and derivation given by Stephens and Krebs [3]. We characterise each patch type, k, with an expected utility return rate as a function of time spent within the patch, $g_k(t_k)$. We assume that patches are encountered randomly with rate λ_k as Poisson processes. The forager's decision is now how long to spend in each patch type, with a strategy described as $\mathbf{t} = [t_1, t_2, ..., t_k]$ ($t_i = 0$ meaning the patch is ignored) . We can write the expected patch utility rate as

$$R_{media} = \frac{\sum_k \lambda_k g_k(t_k)}{1 + \sum_k \lambda_k t_k} \,. \tag{7}$$

Similarly to the prey choice derivation, we differentiate with respect to the time spent in a patch type, t_j,

$$\frac{\partial R_{media}}{\partial t_j} = \frac{\lambda_j g_j'(t_j)(1+\sum_k \lambda_k t_k) - \lambda_j(\sum_k \lambda_k g_k(t_k))}{(1+\sum_k \lambda_k t_k)^2}\,, \tag{8}$$

where $g_j'(t_j) = \frac{\partial g_j(t_j))}{\partial t_j}$. Setting this equal to zero, we find the maximum R_{env} when

$$g_j'(t_j) = R_{env} \qquad \forall j\,. \tag{9}$$

This is Charnov's marginal value theorem [4] and states that an optimal forager will leave a patch when the marginal utility rate of the patch equals the overall rate of utility from foraging in the environment. And foragers will not spend any time in a patch if the marginal rate never reaches the environmental rate i.e. $g_j'(t_j) < R_{env} \quad \forall t_j$. This makes sense intuitively — time spent in a patch with rate g_j carries an opportunity cost of time not spent foraging in the wider environment with utility rate R_{env}.

We can find which patches will be visited using the "patches as prey" algorithm [3]. This is a similar algorithm to the diet choice model but with patches ranked in order of their maximum profitability, $\frac{g_k(t_k^*)}{t_k^*}$. patch types are added to the diet one at a time, with the marginal value theorem applied to all included patches after adding each new patch to recalculate the environmental utility rate. This is done with all patch types, or until Inequality 9 fails.

How would this model of patches effect the conclusions of the main paper? As in the main paper, we assume that media producers have an incentive to create information patches that attract and hold attention. People are still driven towards patches with high patch utility rates. If patch degradation occurs through consuming the most attractive items first then then there would still be a selective pressure toward high utility rate information items, as this would make the patch more attractive before degradation and keep foragers in the patch for longer as it degrades. And this pressure would still apply more strongly to short-form media than long-form media (due to more time switching between short-form media). The conclusions in the main paper would still follow, although the full model would be more complicated. We are confident that the conclusions would hold under any reasonable model of patch degradation.

Supplementary Note 4: The Merged Poisson Process for Patches

Here we justify using average values to describe the expected patch utility rates, instead of summations over information types. We have not seen this derivation before in the foraging literature, but it is relatively straightforward. The result is used without derivation in [7].

In the main text we write down an equation for the expected patch rate in terms of the characteristics of the information within the patch diet, D,

$$R_{media} = \frac{\sum_{i\in D} \lambda_i u_i}{1+\sum_{i\in D} \lambda_i t_i}\,. \tag{10}$$

In this model, information types are encountered as independent Poisson processes with rates, λ_i, during time spent searching, with total searching time T_s. Items have utilities u_i and handling times t_i. With some simple algebraic manipulation we can write down

$$R_{media} = \frac{(\sum_D \lambda_i)\frac{\sum_D \lambda_i u_i T_s}{\sum_D \lambda_i T_s}}{1+(\sum_D \lambda_i)\frac{\sum_D \lambda_i t_i T_s}{\sum_D \lambda_i T_s}}\,. \tag{11}$$

The rate of a combined Poisson process is equal to the sum of the rate of the independent Poisson processes, $\lambda_p = \sum_D \lambda_i$ [8].

We define the average utility of items encountered in the patch as the total utility gained divided by the total number of items handled,

$$\bar{u}_p = \frac{\sum_D \lambda_i u_i T_s}{\sum_D \lambda_i T_s}\,. \tag{12}$$

Similarly the average time spent handling items encountered is the total time spent handling divided by the number of items handled,

$$\bar{t}_p = \frac{\sum_D \lambda_i t_i T_s}{\sum_D \lambda_i T_s}\,. \tag{13}$$

Substituting these relations into equation 11,

$$R_{media} = \frac{\lambda_p \bar{u}_p}{1 + \lambda_p \bar{t}_p} . \tag{14}$$

We can therefore replace the patch rate equation (equation 10) with averages taken over the merged Poisson process. This is a variation of Holling's disc equation [9], considering average values.

Supplementary Note 5: Extended Data

COHA Timeseries for Type Token Ratio, Zipf exponent and Bigram Entropy

In the main article we present smoothed timeseries for the word entropy in the Corpus of Historical American English (COHA). Here we present equivalent timeseries for the type token ratio (Supplementary Figure 2), the Zipf exponent (Supplementary Figure 3), and the bigram entropy as calculated with the plug-in estimator (Supplementary Figure 4). The timeseries were generated as described in the Materials and Methods section of the article.

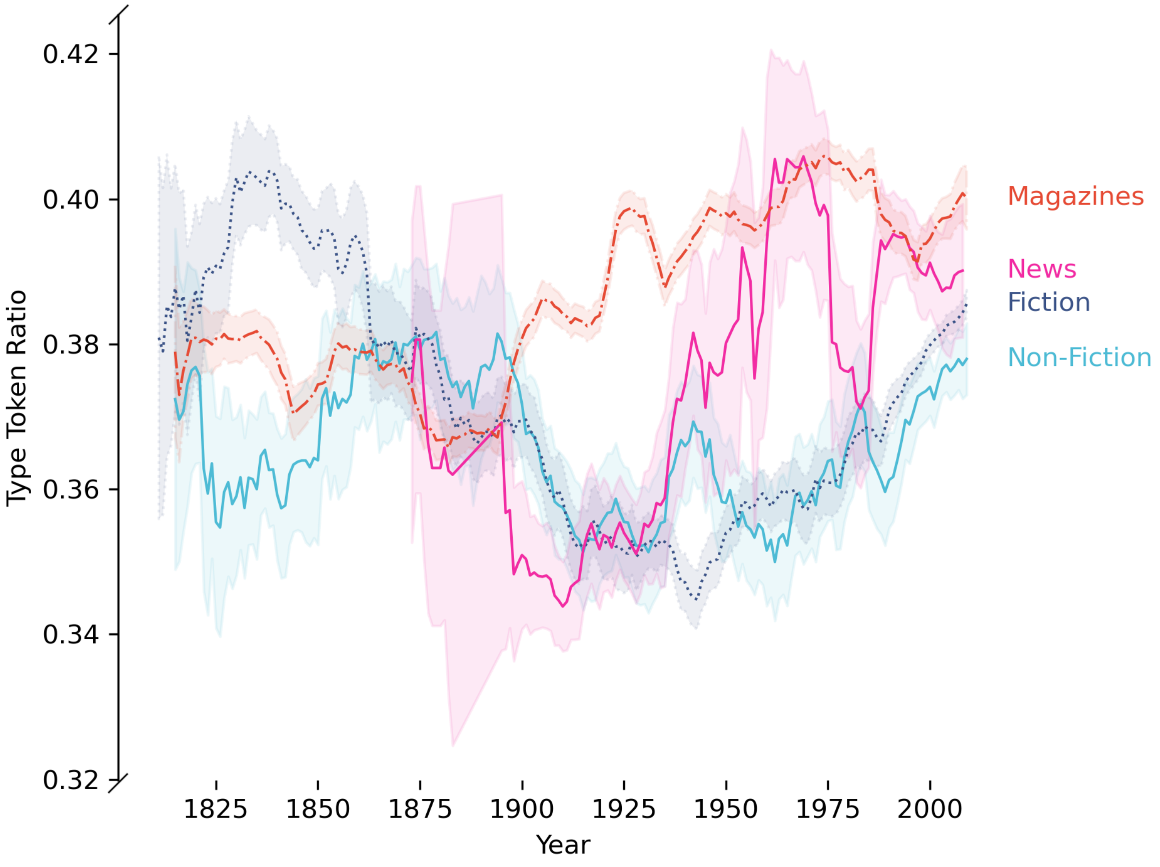

Supplementary Figure 2: Trends of increasing type token ratio across media categories since around 1900. Time series of type token ratio of text samples from the Corpus of Historical American English across media categories of magazines (n=11,306), news (n=720), fiction (n=8,162) and non-fiction (n=2,045). Type token ratio was calculated for text samples from COHA truncated with N = 2000 words. For each media category and year, a moving average of all valid samples with ±5 years was calculated. The shaded region shows a 95% confidence interval for this average.

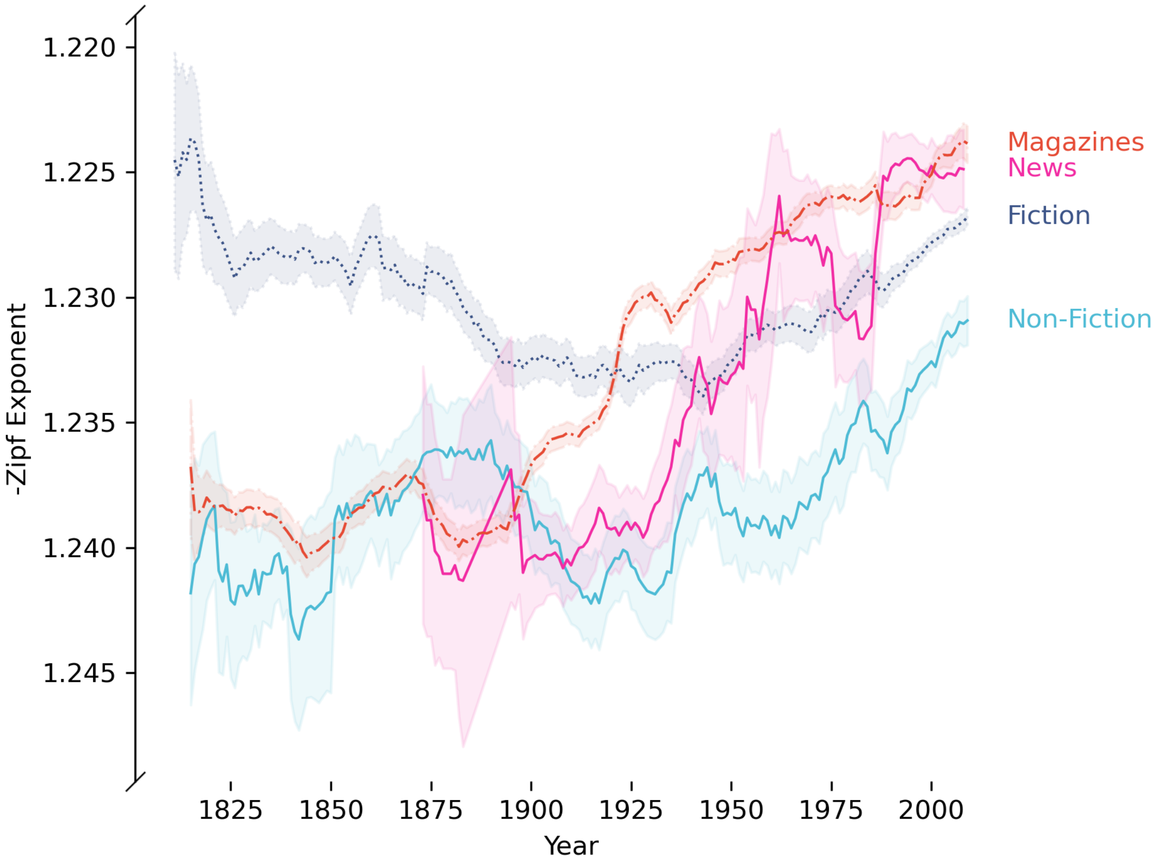

Supplementary Figure 3: Trends of decreasing Zipf exponent across media categories since around 1900. Time series of the Zipf exponent of text samples from the Corpus of Historical American English across media categories of magazines (n=11,306), news (n=720), fiction (n=8,162) and non-fiction (n=2,045). Zipf exponent was estimated for text samples from COHA truncated with $N = 2000$ words. For each media category and year, a moving average of all valid samples with ±5 years was calculated. The shaded region shows a 95% confidence interval for this average.

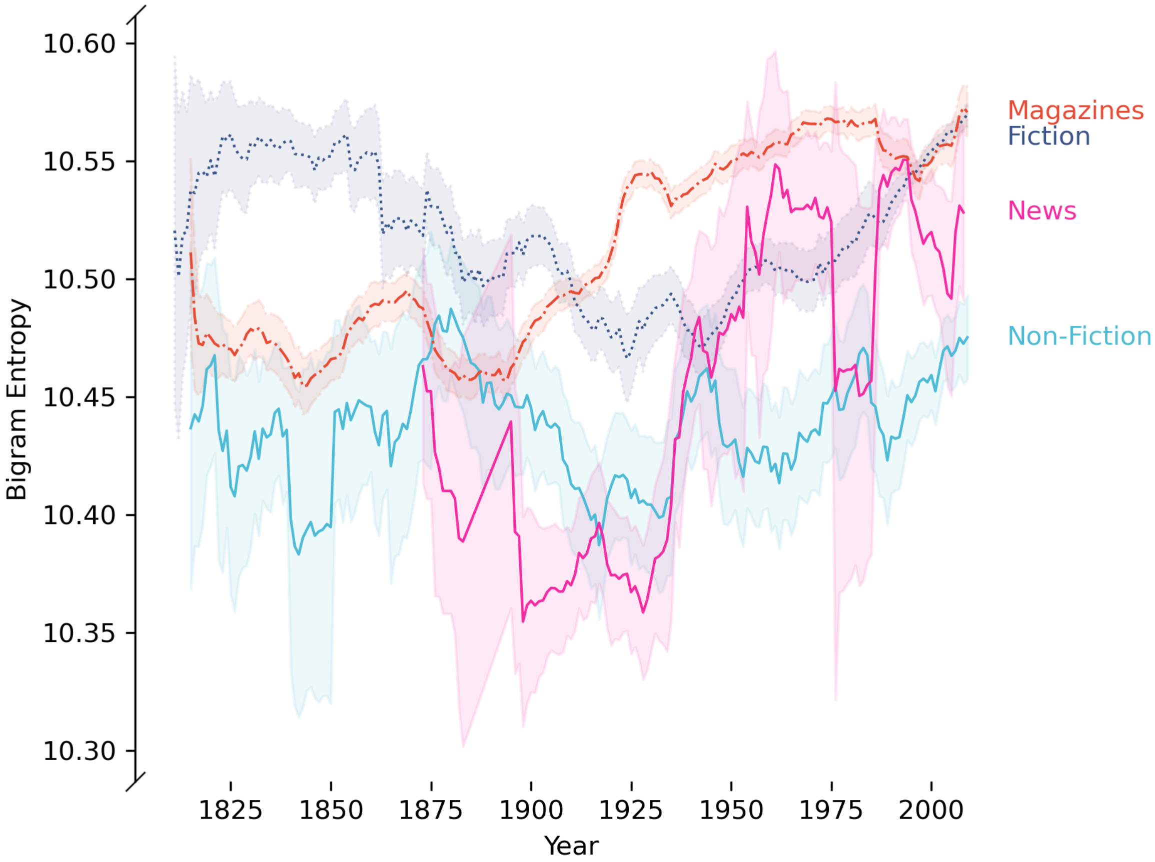

Supplementary Figure 4: Trends of increasing bigram entropy across media categories since around 1900. Time series of the bigram entropy of text samples from the Corpus of Historical American English across media categories of magazines (n=11,306), news (n=720), fiction (n=8,162) and non-fiction (n=2,045). Bigram entropy was estimated for text samples from COHA truncated with $N = 2000$ words. For each media category and year, a moving average of all valid samples with ±5 years was calculated. The shaded region shows a 95% confidence interval for this average.

Corpora Distributions for Word Entropy, Type Token Ratio and Zipf exponent

In the main article we present distributions of the word entropy across different corpora and media categories. Here we present the same for the type token ratio (Supplementary Figure 5, the Zipf exponent (Supplementary Figure 6) and the bigram entropy (Supplementary Figure 7). The same text samples are used as in the main article, and the distributions are generated as described in the Materials and Methods section.

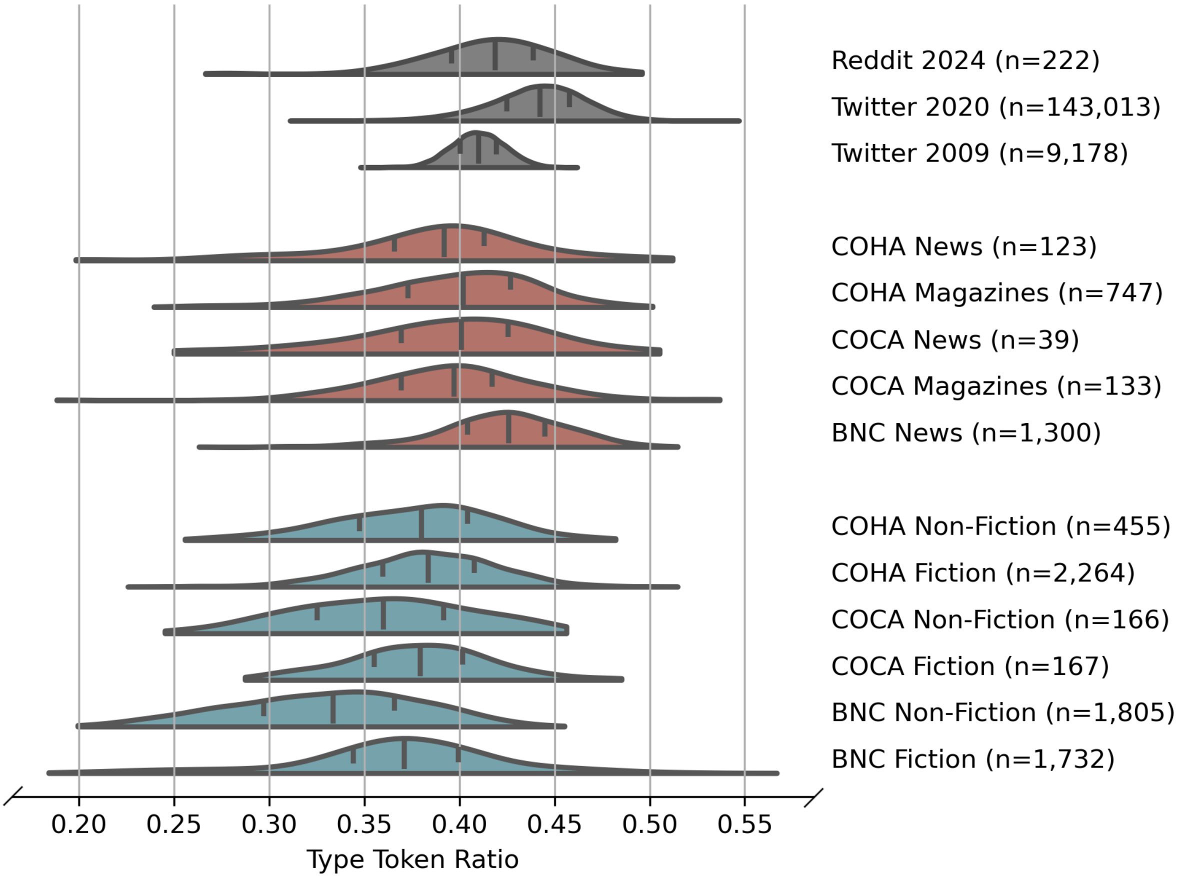

Supplementary Figure 5: Short-form media has higher type token ratio. Distribution snapshots of type token ratio across different text corpora for text samples with $N = 2000$ words. Social media text samples were collated from status updates. For each media category, distributions are kernel density estimates cut to the data range, with quartile positions shown. The COHA data was restricted to 2000-2007 to minimise the effect of historical changes.

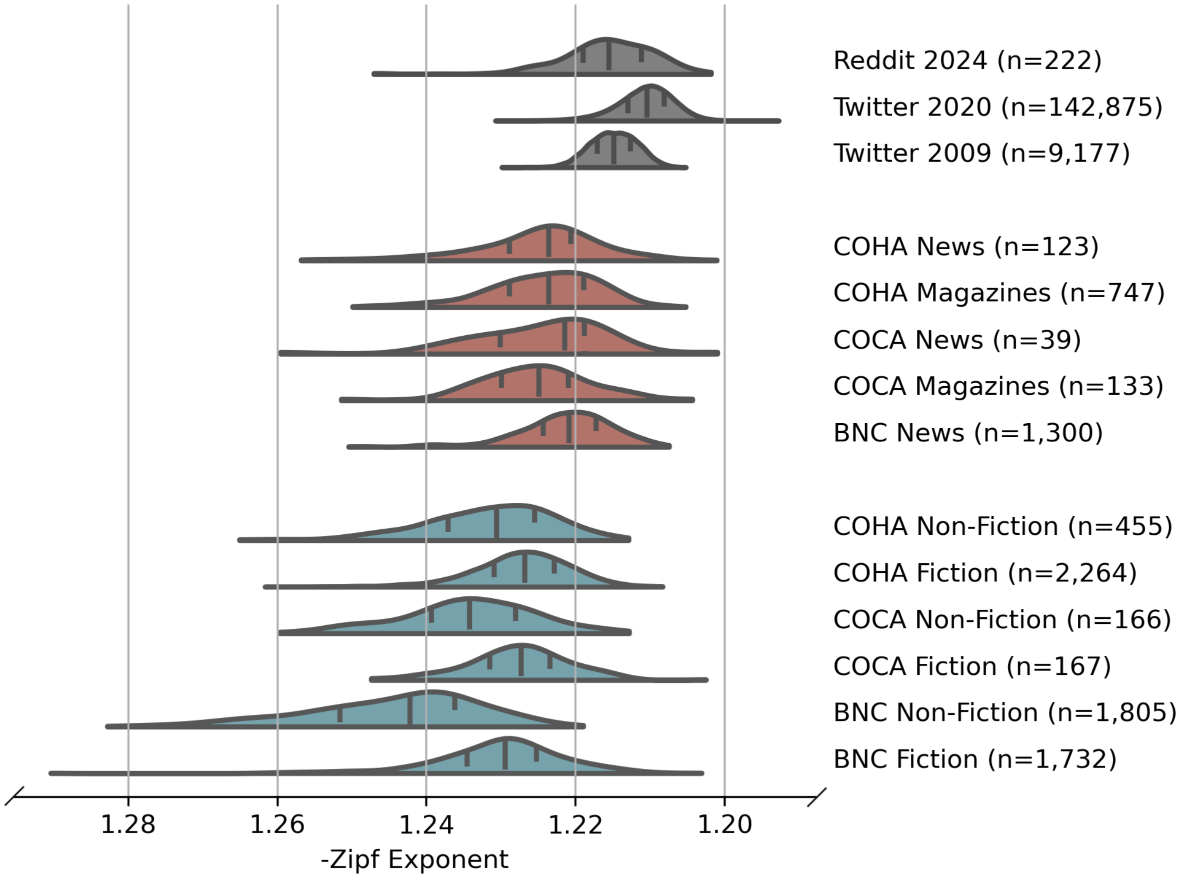

Supplementary Figure 6: Short-form media has lower Zipf exponent. Distribution snapshots of Zipf exponent across different text corpora for text samples with $N = 2000$ words. Social media text samples were collated from status updates. For each media category, distributions are kernel density estimates cut to the data range, with quartile positions shown. The COHA data was restricted to 2000-2007 to minimise the effect of historical changes.

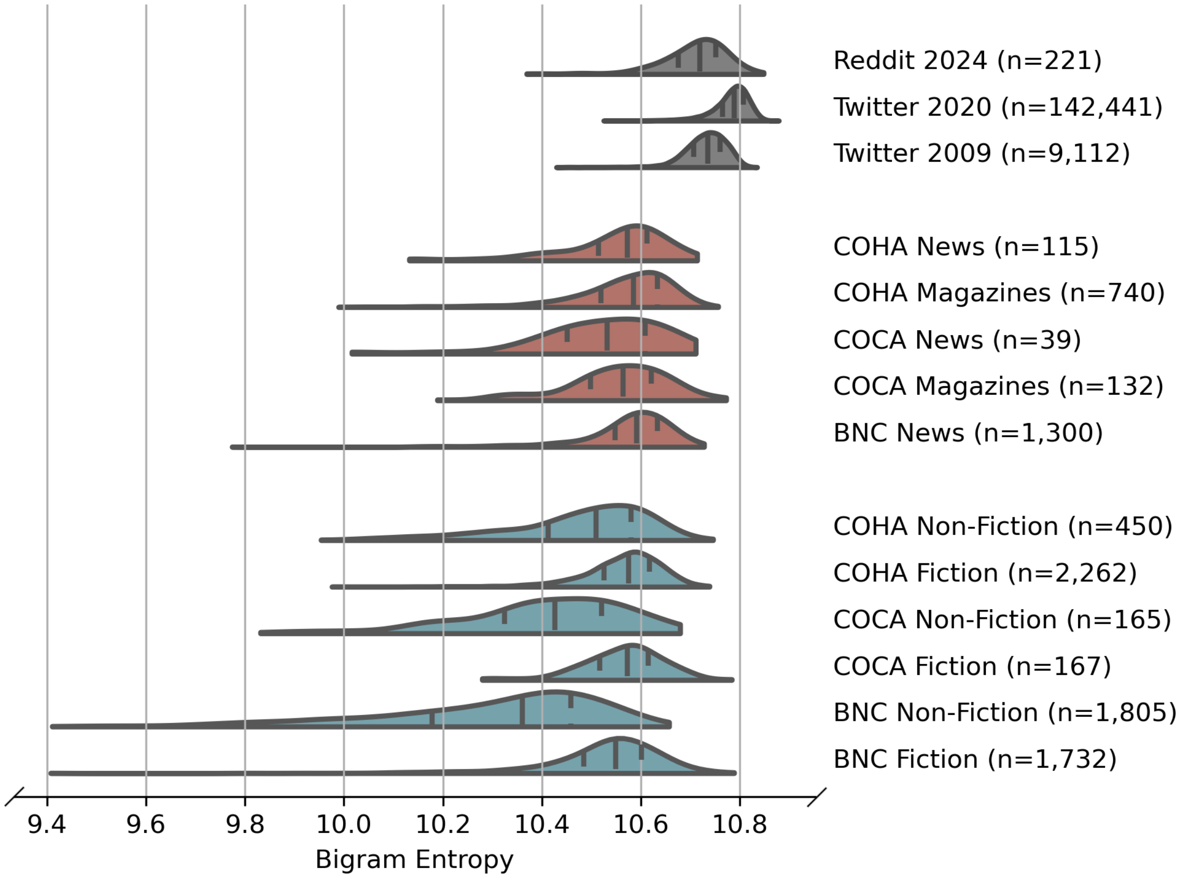

Supplementary Figure 7: Short-form media has higher bigram entropy. Distribution snapshots of bigram entropy across different text corpora for text samples with $N = 2000$ words. Social media text samples were collated from status updates. For each media category, distributions are kernel density estimates cut to the data range, with quartile positions shown. The COHA data was restricted to 2000-2007 to minimise the effect of historical changes.

Supplementary Note 6: Timeseries Breakpoint Analysis

As discussed in Methods, we carried out a piecewise-regression analysis on the median annual values for each of the lexical measures and media categories (Supplementary Figure 8). With the type token ration for the News media category, the breakpoint was found close to the edge of the data. If we restrict the position to avoid being close to the edge then the breakpoint is estimated in a similar location as to the Word Entropy and Zipf exponent. The short-form media shows signs of a rise in lexical diversity before long-form media, consistent with the model in the main paper.

We ran the same analysis with the media categories collated to give an average mean each year (Supplementary Figure 9). Notably, the confidence interval for the breakpoint includes the year 1900.

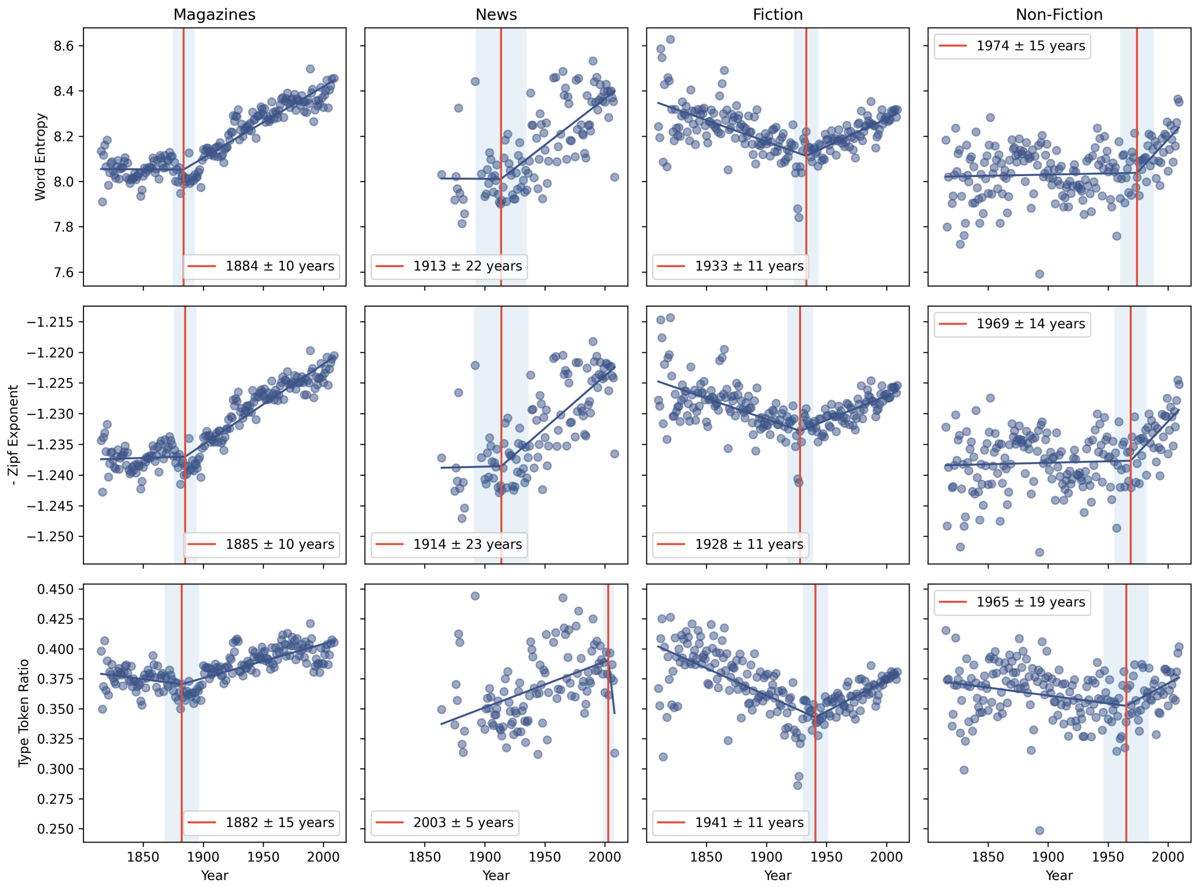

Supplementary Figure 8: Median annual values for each category and lexical measure. The points were fit with a piecewise-regression, with red lines showing the estimated breakpoints. The shaded region shows a 95% confidence interval for those breakpoints.

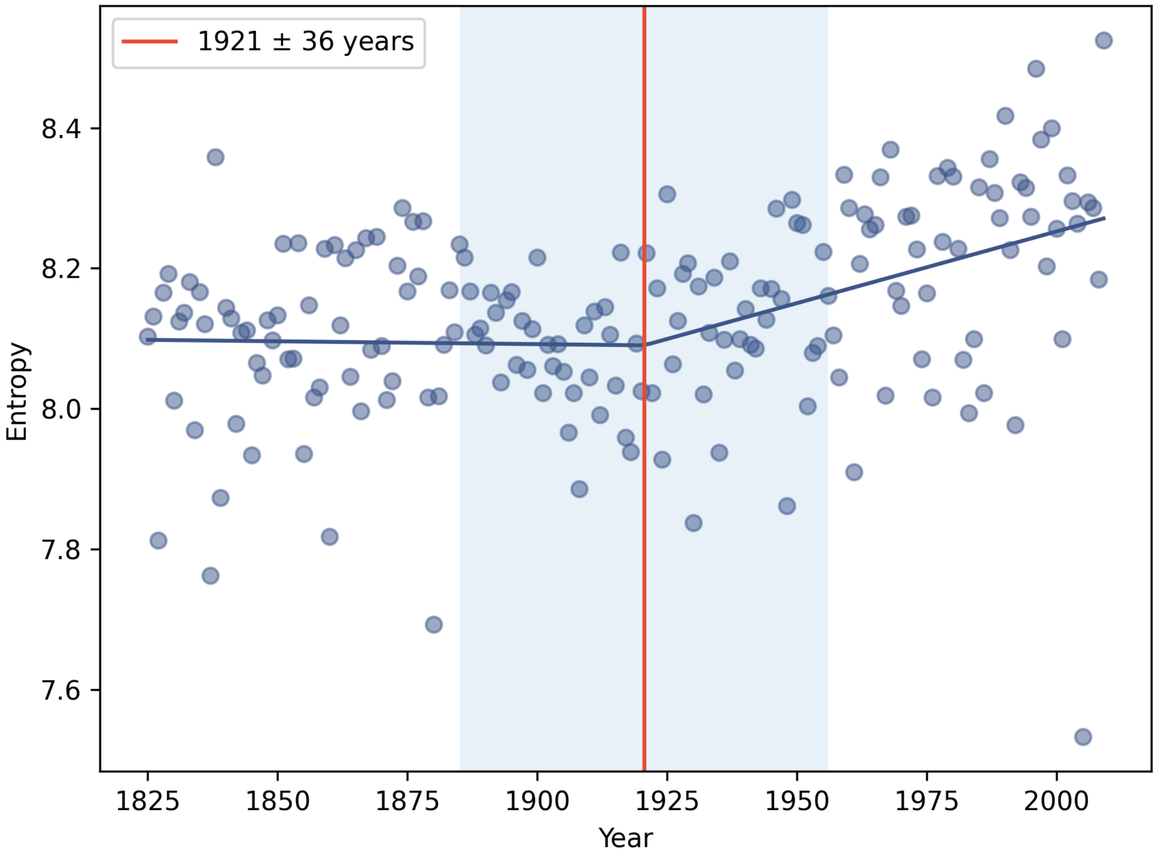

Supplementary Figure 9: Mean annual values for the media categories combined for word entropy. Annual means were first found within each media category, and then averaged over the media categories. The points were fit with a piecewise-regression, with red lines showing the estimated breakpoint. The shaded region shows a 95% confidence interval for that breakpoint.

Supplementary References

[1] David S. Evans. The Economics of Attention Markets. April 2020.

[2] David E Sumner. *The magazine century: American magazines since 1900*, volume 9. Peter Lang, 2010.

[3] David W Stephens and John R Krebs. *Foraging theory*, volume 1. Princeton University Press, 1986.

[4] Eric L. Charnov. Optimal foraging, the marginal value theorem. *Theor. Popul. Biol.*, 9(2):129–136, Apr 1976.

[5] Robert L Bettinger and Mark N Grote. Marginal value theorem, patch choice, and human foraging response in varying environments. *Journal of Anthropological Archaeology*, 42:79–87, 2016.

[6] Horst Pöttker. News and its communicative quality: the inverted pyramid—when and why did it appear? *Journalism Studies*, 4(4):501–511, 2003.

[7] Peter LT Pirolli. *Information foraging theory: Adaptive interaction with information.* Oxford University Press, 2009.

[8] Robert G Gallager. *Discrete stochastic processes*, volume 321. Springer Science & Business Media, 2012.

[9] C. S. Holling. Some characteristics of simple types of predation and parasitism. *The Canadian Entomologist*, 91(7):385–398, July 1959.
