# Supplementary material for: The rising entropy of English in the attention economy
Source: Commun Psychol. 2024 Aug 1;2:70. doi: 10.1038/s44271-024-00117-1 (PMC11332035; doi:10.1038/s44271-024-00117-1)
Supplement: Supplementary file 3 — Reporting Summary [file 44271_2024_117_MOESM3_ESM.pdf]

nature portfolio

Corresponding author(s): Charlie Pilgrim

Last updated by author(s): May 21, 2024

Reporting Summary

Nature Portfolio wishes to improve the reproducibility of the work that we publish. This form provides structure for consistency and transparency in reporting. For further information on Nature Portfolio policies, see our Editorial Policies and the Editorial Policy Checklist.

Statistics

For all statistical analyses, confirm that the following items are present in the figure legend, table legend, main text, or Methods section.

n/a	Confirmed	
☐	☒	The exact sample size (*n*) for each experimental group/condition, given as a discrete number and unit of measurement
☐	☒	A statement on whether measurements were taken from distinct samples or whether the same sample was measured repeatedly
☐	☒	The statistical test(s) used AND whether they are one- or two-sided<br>*Only common tests should be described solely by name; describe more complex techniques in the Methods section.*
☐	☒	A description of all covariates tested
☐	☒	A description of any assumptions or corrections, such as tests of normality and adjustment for multiple comparisons
☐	☒	A full description of the statistical parameters including central tendency (e.g. means) or other basic estimates (e.g. regression coefficient) AND variation (e.g. standard deviation) or associated estimates of uncertainty (e.g. confidence intervals)
☐	☒	For null hypothesis testing, the test statistic (e.g. *F*, *t*, *r*) with confidence intervals, effect sizes, degrees of freedom and *P* value noted<br>*Give P values as exact values whenever suitable.*
☐	☒	For Bayesian analysis, information on the choice of priors and Markov chain Monte Carlo settings
☐	☒	For hierarchical and complex designs, identification of the appropriate level for tests and full reporting of outcomes
☐	☒	Estimates of effect sizes (e.g. Cohen's *d*, Pearson's *r*), indicating how they were calculated

Our web collection on statistics for biologists contains articles on many of the points above.

Software and code

Policy information about availability of computer code

Data collection	All code used to collect data is available at https://github.com/chasmani/PUBLIC-rising-entropy-of-english.<br>For API access, we used python (version 3.9.0) and the requests library (version 2.28.0).
Data analysis	All code used to generate figures and analysis is available at https://github.com/chasmani/PUBLIC-rising-entropy-of-english.<br>We used python (version 3.9.0) and the following packages and versions (beautifulsoup4==4.12.2, nltk==3.7, pandas==1.5.3, scipy==1.10.1, seaborn==0.12.2)<br>We used code from project Gutenberg (https://github.com/c-w/gutenberg/) (version 0.8.2).

For manuscripts utilizing custom algorithms or software that are central to the research but not yet described in published literature, software must be made available to editors and reviewers. We strongly encourage code deposition in a community repository (e.g. GitHub). See the Nature Portfolio guidelines for submitting code & software for further information.

Data

Policy information about availability of data

All manuscripts must include a data availability statement. This statement should provide the following information, where applicable:

- Accession codes, unique identifiers, or web links for publicly available datasets
- A description of any restrictions on data availability
- For clinical datasets or third party data, please ensure that the statement adheres to our policy

All data generated following analysis of text samples is available at https://github.com/chasmani/PUBLIC-rising-entropy-of-english.
The text corpora data is not included in the public repository for copyright and size reasons. They are available:
• COHA and COCA. https://www.corpusdata.org/
• BNC. http://www.natcorp.ox.ac.uk/
• Twitter 2009 dataset. https://www.kaggle.com/kazanova/sentiment140
• Twitter 2020 dataset. This was collected from Twitter's API between February 2020 and January 2021. We cannot provide the data for copyright reasons.
• Reddit dataset. This was collected from Reddit's API between 5th to 17th January 2024. We cannot provide the data for copyright reasons. https://www.reddit.com/.json.

Research involving human participants, their data, or biological material

Policy information about studies with human participants or human data. See also policy information about sex, gender (identity/presentation), and sexual orientation and race, ethnicity and racism.

Reporting on sex and gender	We did not carry out and sex or gender based analysis.
Reporting on race, ethnicity, or other socially relevant groupings	We did not use any groupings like this.
Population characteristics	N/a
Recruitment	N/a
Ethics oversight	N/a

Note that full information on the approval of the study protocol must also be provided in the manuscript.

Field-specific reporting

Please select the one below that is the best fit for your research. If you are not sure, read the appropriate sections before making your selection.

☐ Life sciences ☒ Behavioural & social sciences ☐ Ecological, evolutionary & environmental sciences

For a reference copy of the document with all sections, see nature.com/documents/nr-reporting-summary-flat.pdf

Behavioural & social sciences study design

All studies must disclose on these points even when the disclosure is negative.

Study description	Quantitative analysis of historical data.
Research sample	We used datasets:<br>- COHA and COCA. https://www.corpusdata.org/<br>• BNC. http://www.natcorp.ox.ac.uk/<br>• Twitter 2009 dataset. https://www.kaggle.com/kazanova/sentiment140<br>• Twitter 2020 dataset. This was collected from Twitter's API between February 2020 and January 2021. We cannot provide the data for copyright reasons.<br>• Reddit dataset.This was collected from Reddit's API between 5th to 17th January 2024. We cannot provide the data for copyright reasons. https://www.reddit.com/.json.
Sampling strategy	We used all data available to us.
Data collection	N/a. Data downloaded from existing sources.
Timing	Twitter 2020 dataset. This was collected from Twitter's API between February 2020 and January 2021. We cannot provide the data for copyright reasons.<br>Reddit dataset.This was collected from Reddit's API between 5th to 17th January 2024. We cannot provide the data for copyright

	reasons. https://www.reddit.com/.json.
Data exclusions	We removed outliers that had values of lexical measures that were more than 5 standard deviations from the mean of the corpus (less than 0.1\% of the data).
Non-participation	N/a. No participants.
Randomization	N/a. No participants.

Reporting for specific materials, systems and methods

We require information from authors about some types of materials, experimental systems and methods used in many studies. Here, indicate whether each material, system or method listed is relevant to your study. If you are not sure if a list item applies to your research, read the appropriate section before selecting a response.

Materials & experimental systems

n/a	Involved in the study
☒	☐ Antibodies
☒	☐ Eukaryotic cell lines
☒	☐ Palaeontology and archaeology
☒	☐ Animals and other organisms
☒	☐ Clinical data
☒	☐ Dual use research of concern
☒	☐ Plants

Methods

n/a	Involved in the study
☒	☐ ChIP-seq
☒	☐ Flow cytometry
☒	☐ MRI-based neuroimaging

Plants

Seed stocks	n/a
Novel plant genotypes	n/a
Authentication	n/a
