# Supplementary material for: The rising entropy of English in the attention economy
Source: Commun Psychol. 2024 Aug 1;2:70. doi: 10.1038/s44271-024-00117-1 (PMC11332035; doi:10.1038/s44271-024-00117-1)
Supplement: Supplementary file 1 — Peer Review File [file 44271_2024_117_MOESM1_ESM.pdf]

nature portfolio

Peer Review File

The rising entropy of English in the Attention Economy

Open Access This file is licensed under a Creative Commons Attribution 4.0 International License, which permits use, sharing, adaptation, distribution and reproduction in any medium or format, as long as you give appropriate credit to the original author(s) and the source, provide a link to the Creative Commons license, and indicate if changes were made. In the cases where the authors are anonymous, such as is the case for the reports of anonymous peer reviewers, author attribution should be to 'Anonymous Referee' followed by a clear attribution to the source work. The images or other third party material in this file are included in the article's Creative Commons license, unless indicated otherwise in a credit line to the material. If material is not included in the article's Creative Commons license and your intended use is not permitted by statutory regulation or exceeds the permitted use, you will need to obtain permission directly from the copyright holder. To view a copy of this license, visit http://creativecommons.org/licenses/by/4.0/.

Web links to the author's journal account have been redacted from the decision letters as indicated to maintain confidentiality

16th Dec 22

Dear Mr Pilgrim,

Thank you for submitting your manuscript titled "The Rising Entropy of English in the Attention Economy" and thank you for your patience in awaiting an initial decision. I have now had the opportunity to read your work. Regretfully, we cannot publish this submission in Communications Psychology, at least not in its present form.

We would, in principle, be interested in sending your work out to peer review. However, because of concerns about the origin of the data currently presented in the manuscript, we cannot proceed to peer review.

Your data analysis of language use in the attention economy relies on two datasets, which you accessed via https://www.kaggle.com/kazanova/sentiment140 (Twitter dataset) and https://github.com/minimaxir/interactive-facebook-reactions (Facebook dataset).

The Twitter data on kaggle were obtained by the original curator via Twitter's API, which is appropriate. However, the Facebook data were "scraped", according to the documentation provided by the original curator.

Scraping Facebook data is not permitted (https://en-gb.facebook.com/legal/terms), which means that we cannot consider manuscripts that (re)use such data, regardless of whether the authors obtained it themselves via scraping or retrieved from a public repository.

To use the Facebook data in a manuscript you would need to make an application to request access from Facebook.

Please note that use of Facebook data is not a pre-condition for peer-review. If you removed the Facebook data and complemented your Twitter data by a different dataset from another suitable platform, we would consider your work for peer review.

I would be grateful if you let us know how you choose to proceed and thank you for your interest in publishing with us.

Best regards,

Marike Schiffer

7th Jun 23

Dear Charlie,

Thank you for your patience during the peer-review process. Your manuscript titled "The Rising Entropy of English in the Attention Economy" has now been seen by 2 reviewers, whose comments are appended below. I am sorry in the delay in getting back to you with a decision, which came about as a result of reviewer unavailability.

You will see that the referees are enthusiastic about your work, but also highlight a number of issued that need to be addressed. In light of these comments, we cannot accept the manuscript for publication in its present form, but would be interested in considering a revised version that fully addresses these serious concerns.

Please bear in mind that we will be reluctant to approach the reviewers again in the absence of substantial revisions that address the referees' comments, and follow editorial guidance.

It is a priority for the journal that its publications present strong appropriate evidence for the main claims in the paper. We therefore ask that, in response to Reviewer #1's concern about the age of the dataset, you conduct a replication in a more recent Twitter dataset. Moreover, Reviewer #2 highlights some concerns regarding the entropy measure. Here we ask that you follow their advice for a comparative analysis with bigram entropy.

The referees make a large number of constructive suggestions regarding the formatting of the paper and the presentation of existing literature. We strongly encourage you to comprehensively address these request. As you do so, please bear in mind that the journal has clear formatting and reporting guidelines, which you will find in the template and checklist below. Please also address Referee #2's concerns regarding the correction for multiple comparisons and as you do so, ensure that all statistics reporting and interpretation follows our guidelines.
https://www.nature.com/commspsychol/submit/submission-guidelines#statistical-guidelines

Finally, when you address the referees' suggestions for a more comprehensive discussion of the results, please bear in mind that we require a section in the Discussion titled "limitations" and that we do ask authors to avoid novelty claims and limit speculation.

If the revision process takes significantly longer than five months, we will be happy to reconsider your paper at a later date, provided it still presents a significant contribution to the literature at that stage.

We would appreciate it if you could keep us informed about an estimated timescale for resubmission, to facilitate our planning.

We are committed to providing a fair and constructive peer-review process. Please do not hesitate to contact us if you wish to discuss the revision in more detail.

Please use the following link to submit your revised manuscript, point-by-point response to the Reviewers' comments with a list of your changes to the manuscript text (which should be in a separate document to any cover letter) and any completed checklist:

[link redacted]

** This url links to your confidential home page and associated information about manuscripts you may have submitted or be reviewing for us. If you wish to forward this email to co-authors, please delete the link to your homepage first **

Please do not hesitate to contact me if you have any questions or would like to discuss the required revisions further. Thank you for the opportunity to review your work.

Best regards,

Marike

Marike Schiffer, PhD

Chief Editor

Communications Psychology

EDITORIAL POLICIES AND FORMATTING

We ask that you ensure your manuscript complies with our editorial policies. Please ensure that the following formatting requirements are met, and any checklist relevant to your research is completed and uploaded as a Related Manuscript file type with the revised article.

Editorial Policy: Policy requirements (Download the link to your computer as a PDF.)

Furthermore, please align your manuscript with our format requirements, which are summarized on the following checklist:

Communications Psychology formatting checklist

and also in our style and formatting guide Communications Psychology formatting guide .

* TRANSPARENT PEER REVIEW: Communications Psychology uses a transparent peer review system. This means that we publish the editorial decision letters including Reviewers' comments to the authors and the author rebuttal letters online as a supplementary peer review file. However, on author request, confidential information and data can be removed from the published reviewer reports and rebuttal letters prior to publication. If your manuscript has been previously reviewed at another journal, those Reviewers' comments would not form part of the published peer review file.

* CODE AVAILABILITY: All Communications Psychology manuscripts must include a section titled "Code Availability" at the end of the methods section. In the event of publication, we require that the custom analysis code supporting your conclusions is made available in a publicly accessible repository; please choose a repository that provides a DOI for the code; the link to the repository and the DOI must be included in the Code Availability statement. Publication as Supplementary Information will not suffice. We ask you to prepare and upload code at this stage, to avoid delays later on in the process.

* DATA AVAILABILITY:

All Communications Psychology research manuscripts must include a section titled "Data Availability" at the end of the Methods section or main text (if no Methods). More information on this policy, is available at http://www.nature.com/authors/policies/data/data-availability-statements-data-citations.pdf.

At a minimum the Data availability statement must explain how the data can be obtained and whether there are any restrictions on data sharing. Communications Psychology strongly endorses open sharing of da. If you do make your data openly available, please include in the statement:

- Unique identifiers (such as DOIs and hyperlinks for datasets in public repositories)
- Accession codes where appropriate
- If applicable, a statement regarding data available with restrictions
- If a dataset has a Digital Object Identifier (DOI) as its unique identifier, we strongly encourage including this in the Reference list and citing the dataset in the Data Availability Statement.

We recommend submitting the data to discipline-specific, community-recognized repositories, where possible and a list of recommended repositories is provided at http://www.nature.com/sdata/policies/repositories.

If a community resource is unavailable, data can be submitted to generalist repositories such as figshare or Dryad Digital Repository. Please provide a unique identifier for the data (for example a DOI or a permanent URL) in the data availability statement, if possible. If the repository does not provide identifiers, we encourage authors to supply the search terms that will return the data. For data that have been obtained from publicly available sources, please provide a URL and the specific data product name in the data availability statement. Data with a DOI should be further cited in the methods reference section.

Please refer to our data policies at http://www.nature.com/authors/policies/availability.html.

REVIEWER EXPERTISE:

Reviewer #1: media, communication, psycholinguistics

Reviewer #2: psycholinguistics, language evolution, computational methods

Reviewer #1 (Remarks to the Author):

I have read the paper "The Rising Entropy of English in the Attention Economy" with great interest and I find it to be a well-written and thought-provoking contribution to the field of sociolinguistics. The study provides compelling evidence that word entropy in American English has been steadily increasing since around 1900, contrary to existing theories, and that this trend is driven by factors such as technological change and information foraging.

In the following paragraphs, I will provide a detailed analysis and comments on the three bis sections of the draft, along with a brief comment on the supplementary information provided by the authors and some last thoughts. My suggestions for improvement are relatively minor and should be considered as an opportunity to enhance an already sound study.

Materials and Methods (p. 2)

I would like to commend the authors on their thorough approach to data collection, cleaning, processing, and analysis. The rigorous treatment of data sets is evident in the methodologies outlined, which encompass a broad range of corpus data and social media inputs. Each step of the process is given due consideration and this commitment to precision and detail fosters confidence in the overall credibility of the research.

The choice of text corpora is diverse and well-reasoned, covering a wide temporal and geographical range and including different genres and categories. The measures taken to clean the text sample data were appropriate and methodical. Particularly, the application of python's natural language toolkit (nltk) package to convert text to tokens, and selection of the last 2000 tokens for processing demonstrates a thoughtful approach to text analysis. Also noteworthy is the authors' diligent application of data cleaning measures for the COHA, COCA, and BNC datasets. The consistent application of procedures across all data sources underpins the credibility of subsequent analyses and is a testament to the authors' commitment to scientific rigour.

The selection and treatment of social media data from Twitter and Reddit were initially deemed robust. The innovative collation strategy employed to simulate a social media feed appeared promising, providing a potential means to capture the richness of these data sources. The authors also demonstrated an awareness of the unique considerations associated with social media text, such as the cleaning process that accounted for URLs, hashtags, and usernames.

However, it has come to my attention that the collected tweets are from 2009, while the Reddit posts were collected in March 2023. This raises concerns for me as we could potentially be dealing with outdated (Twitter) or inaccurate data (Reddit). For instance, considering Figure 3, Reddit's top position in terms of Word Entropy, compared to Twitter, could arguably be attributed to the timing of data collection. Notably, the most recent data analyzed in this draft are the Reddit posts. However, the data collection end period for Twitter, COHA, COCA, and BNC corpora would be at least from approximately 13-14 years ago. It is essential to consider that during this time, Twitter was only three years old (established in March 2006), and we are juxtaposing it with the data from a social platform that, having been established in June 2005, was already almost 18 years old in 2023. Both the technical options and the ethos of the platforms, users and content, may have significantly evolved during this period. This contrast in age and context between the Twitter data and the Reddit data should be taken into account, as it introduces a certain qualitative arbitrariness in the study's core. We will revisit this issue later in our final thoughts. For now, I would like to offer one more suggestion, namely, to include the date of the Reddit data collection in the first of the two Social Media Data subsections (p. 3), paralleling what has already been done with the Twitter data. I only found the Reddit's collection information in the Data Availability subsection (i.e., on p. 12).

Moving forward, the authors' choice of using unigram word entropy as a measure of information evolution is a solid choice. Additionally, their application of the maximum likelihood estimator, as well as their consideration of both the type token ratio and Zipf exponent as measures of lexical diversity, alludes to a comprehensive and meticulous approach towards understanding the nuances of the text.

In terms of timeseries analysis, the decision to apply both the KPSS and MK tests to explore trends in lexical measures over time is a sound approach, providing a well-rounded understanding of the data’s underlying structures. I appreciate the careful choice of the KPSS test, which is particularly suited to detect stationarity in a time series, and the MK test, which is a reliable non-parametric trend test.

The employment of timeseries smoothing, using a moving average method, provides a clearer visual representation of the data and enhances the interpretability of the results. The authors' decision to only include years with at least 10 data points within the window and their meticulous calculation of the confidence interval, demonstrates a thoughtful consideration of statistical robustness.

The examination of differences between media categories using ANOVA tests also demonstrates a strong methodological approach. However, I would suggest that the authors provide a deeper explanation or justification for the choice of the Scott rule in the density estimate.

Finally, the use of magazine circulation data from the Audit Bureau of Circulation lends a degree of real-world grounding to the study. Nonetheless, the authors might want to consider discussing the limitations of this specific dataset and how these might impact their findings.

Overall, in this section the authors demonstrate, setting aside the aforementioned limitations or concerns for a moment, an impressive commitment to methodological rigor, which contributes to the robustness of their ulterior findings.

RESULTS (p. 5)

In my evaluation, the Results section of the paper is appropriately divided into relevant subsections, effectively addressing the key topics of interest. Each subsection serves a unique purpose and contributes to the overall understanding of the subject matter. Here, I discuss these subsections in greater detail, considering their rationale, correctness, and implications.

In the "Higher Entropy in Short-form Media" subsection, the authors argue that the historical trend implies modern differences in entropy between media categories. While they mention the prominence of short-form media in the digital landscape, the ideas could benefit from further elaboration. A more nuanced discussion around the role of attention spans in media consumption, and the impact of algorithms or video feeds, could provide a better-rounded perspective. By addressing some of these aspects, at least as recognized limitations, in the discussion section, the study can offer a more comprehensive analysis of the dynamics of social media consumption. This inclusion would acknowledge the potential impact of attention spans on learning new optimized terms or expressions, as well as the influence of algorithms in shaping content. Additionally, recognizing the growing significance of audiovisual materials on platforms like TikTok, with considerably fewer texts and a larger user base than Reddit, would provide valuable insights. By incorporating these limitations in a manner at the discretion of the authors, the discussion section would contribute to a deeper understanding of the study's findings in the context of contemporary trends in social media usage.

The "Information Foraging in the Attention Economy" subsection exhibits a laudable effort to draw a connection between the competition for attention and word entropy. The authors utilize mathematics to model the attention economy based on information foraging, effectively adapting concepts such as information signal entropy, forager utility, and least effort principle to serve their purpose. While they present a perfectly valid case in linking these concepts, it is crucial to acknowledge that the model might oversimplify human behavior and social complexities. Future studies could delve deeper by incorporating additional factors influencing information

consumption patterns, such as psychological biases, cultural norms, or socio-economic background.

In the "Competition Between Media Platforms Drives Differences Between Short- and Long-form Media" subsection, the authors evoke foraging theory in the context of information consumption and media platforms to explain observed differences in information utility rates. I commend the authors for their efforts in discussing competition, information foraging, and attention economy concepts. While these notions are not entirely new, their combination and translation into clear and informative mathematical models in the paper are innovative, bridging the gap between theoretical concepts and quantitative analysis. However, the simplification of human and social reality should be kept in mind when interpreting and generalizing the results.

In summary, in this section the authors have done a laudable job of presenting a complex topic through a cohesive and coherent framework. Their work paves the way for future studies to address the limitations and expand upon the ideas introduced, ultimately enabling a more comprehensive and nuanced understanding of the connections between entropy, media types, information foraging, and the attention economy.

Discussion (p. 10)

The "Discussion" section of the paper provides fascinating insights and draws important contrasts with existing theories. However, the authors could improve upon certain aspects, particularly in granularity and by briefly referring to relevant interdisciplinary research.

While the attention economy model is introduced with strong rationale, connections to empirical results could be further elaborated. Cross-references to research fields such as Communication Research, which have investigated social media from quantitative and qualitative perspectives, would be beneficial to strengthen their argument and avoid potential oversimplifications.

The discussion on the Linguistic Niche Hypothesis and the evidence of morphological simplification in English is intriguing. Although the authors claim that the pressure towards information density overcomes reduced word entropy from linguistic simplification, a deeper exploration of cause-effect relationships or literature comparisons would provide a more solid foundation for their hypothesis.

As the authors delve into the psychological consequences of language evolution, they touch upon various principles governing language development from biological, cultural, learning, and other perspectives. Nonetheless, the connection to the research question, particularly to word entropy in the context of information abundance, could be more clearly articulated.

Figure 6 in the discussion adds a valuable visual illustration of the relation between minimum average information size and information prevalence. Expanding on the implications of this relationship and its relevance to the current state of digital media and communication would make the discussion more informative and engaging.

The comparison of human information foragers and empirical findings on the attraction to high entropy and complexity is particularly appealing. Notwithstanding, a broader analysis of the competition within the attention economy and media content adaptation for capturing limited attention may prove fruitful.

Lastly, the authors highlight that human choices are influenced by factors beyond entropy, such as social cues and risks. Nevertheless, the relevance of these factors to the present analysis is only briefly touched upon, without sufficiently illustrating their potential impact on language evolution and cultural history. Delving deeper into the interplay between these factors and the theories put forth in the paper would provide a more comprehensive understanding of the subject matter.

In conclusion, the "Discussion" section showcases promising insights and thought-provoking contrasts with existing theories. However, enhancing granularity, connecting empirical results, and exploring interdisciplinary research could further strengthen the section and render a more profound contribution to the field.

Supplementary Information (Additional File)

The provision of supplementary materials alongside the manuscript is appreciated. The supplementary information has proven to be valuable, particularly in relation to sub-sections 3, 4, and 5. The inclusion of detailed information in these sections has enhanced the clarity and depth of the study.

However, it is worth noting that the presence of two subsections, specifically 7 and 8, containing only headers but no content, was surprising, or at least I haven't been able to find an explanation

for this fact. If this oversight is on my part, I apologize in advance to the authors. It is unclear if any indications or justifications were provided by the authors to account for the inclusion of these empty subsections. If indeed both subsection titles are orphaned, i.e. if is ultimately determined that the inclusion of subsections 7 and 8 is not necessary, it would be advisable to remove them.

Final thoughts

In summary, I think the authors' meticulous approach to data preparation, underscored by strategic use of computational tools, meets high standards. Moreover, their transparency in delineating study procedures and protocols bolsters credibility and fosters potential replication by others in the field. Furthermore, they have done an excellent job presenting complex ideas clearly while still leaving plenty open-ended questions ripe for future research. Their work paves the way for future studies to address the limitations and expand upon the ideas introduced, ultimately enabling a more comprehensive and nuanced understanding of the connections between entropy, media types, information foraging, and the attention economy.

Regarding information foraging, it is important to consider a specific aspect that deserves attention. This concerns the use of animal models as a means of predicting human behavior in various domains. Although useful to gain insight into human decision making in specific situations it is crucial to approach these models with care and acknowledge their potential restrictions.Humans exhibit an intricate nature with advanced cognitive skills that exceed those seen in many animals. Our actions and behaviors arise out of multiple factors much more complicated than the drive to satisfy our hunger (Ju et al., 2022). Even though animal foraging routes provide an effective starting point, expanding on this model by considering a more extensive array of components and possibilities becomes necessary when endeavoring to anticipate human conduct across various areas. Therefore, it is also important when writing, not to forget that a model is precisely that: a simplification of reality (Pourfattah et al., 2021). Undoubtedly, I concur with the view that the information foraging and attention competition models illuminate a significant aspect of human behaviour. However, in the discussion section, it might be beneficial to more explicitly address some inherent limitations or reservations linked with this approach.

Likewise, in wrapping up this review, it's necessary to recognise that the attention economy model, while insightful, may be somewhat reductive in its suggestion that human attention is primarily governed by the maximisation of novel information intake. Such a model may overlook the richness and variability of human motivation and behaviour. For instance, emotional engagement often dictates where our attention is directed (Saunders, 2020); a moving film or riveting book tends to seize our focus more readily than an informative brochure, illustrating the power of emotional

narratives in various sectors, from entertainment to brand communication and news consumption (Yang and Hobbs, 2020; Wearn and Shepherd, 2020; Meuer et al., 2023).

Similarly, the influence of social connections on attention allocation is significant, as evidenced by the popularity of socially-oriented media over generic advertisements or updates (Baek and Parkinson, 2022). Information relevant to our social circle frequently captures our attention more than impersonal content. Moreover, elements such as emotional engagement, social connections, personal values, and attitudes each play a pivotal role in shaping decision-making processes and attention allocation, exhibiting complex dynamics rather than a strictly linear relationship, warranting further consideration (Lin et al., 2017; Ponizovskiy, 2020). Another intriguing research direction would involve exploring the impact of gender, education, income, cultural background, and political beliefs on information-foraging behaviors and responses to different media modalities.

In another final note on this review, it is worth mentioning that subsequent research would potentially be enriched by the incorporation of up-to-date data sourced from current, prominent social media platforms. Engaging with such data through the lens of cross-disciplinary approaches and mixed methods could further elucidate the nuanced dynamics of language usage on social media (Sun et al., 2021). Additionally, an interesting avenue for future research could be to discern whether these observed trends are exclusive to English, or if they transcend linguistic boundaries and are observable across a spectrum of other languages.

In conclusion, notwithstanding the discussions presented throughout this review and these final reflections, at its core, the study " The Rising Entropy of English in the Attention Economy " makes a substantial contribution to our understanding of language evolution within the context of the attention economy. While it provides valuable insights into growing entropy and its implications, further clarification regarding the gap between the Twitter and Reddit data, along with addressing the points highlighted in this review, would enhance its impact. Additionally, explicit consideration of potential limitations and broader societal implications would expand the reach of this crucial research area. The synthesis of big data and qualitative approaches mutually reinforce each other, combining conventional methods for contextual understanding with automated analysis of extensive text data, fostering an iterative process that both answers existing questions and generates new ones (Bail, 2014). This collaborative approach holds the potential to advance science, benefit society, and, importantly, positively impact individuals' lives.

Cited Literature

Baek, E. C., & Parkinson, C. (2022). Shared understanding and social connection: Integrating approaches from social psychology, social network analysis, and neuroscience. Social and Personality Psychology Compass, 16(11), e12710. https://doi.org/10.1111/spc3.12710

Bail, C. A. (2014). The cultural environment: measuring culture with big data. Theory and Society, 43(3–4), 465–482. https://doi.org/10.1007/s11186-014-9216-5

Ju, X., Martín Martín, O., & Chocarro, R. (2022). Determinants of mobile social media use, customer heterogeneity, and international microsegmentation. International Journal of Consumer Studies, 46(4), 1127–1148. https://doi.org/10.1111/ijcs.12748

Lin, K.-Y., & Lu, H.-P. (2011). Why people use social networking sites: An empirical study integrating network externalities and motivation theory. Computers in Human Behavior, 27(3), 1152–1161. https://doi.org/10.1016/j.chb.2010.12.009

Meuer, M., Oeberst, A., & Imhoff, R. (2022). How do conspiratorial explanations differ from non-conspiratorial explanations? A content analysis of real-world online articles. European Journal of Social Psychology, 53(2), 288–306. https://doi.org/10.1002/ejsp.2903

Ponizovskiy, V., Ardag, M., Grigoryan, L., Boyd, R., Dobewall, H., & Holtz, P. (2020). Development and validation of the Personal Values Dictionary: A theory–driven tool for investigating references to basic human values in text. European Journal of Personality, 34(5), 885–902. https://doi.org/10.1002/per.2294

Pourfattah, F., Wang, L.-P., Deng, W., Ma, Y.-F., Hu, L., & Yang, B. (2021). Challenges in simulating and modeling the airborne virus transmission: A state-of-the-art review. Physics of Fluids, 33(10), 101302. https://doi.org/10.1063/5.0061469

Saunders, A. (2020). The eventisation of anniversary activity: Investing in 1989. German Life and Letters, 73(3), 464–489. https://doi.org/10.1111/glal.12277

Sun, Y., Wang, G., & Feng, H. (2021). Linguistic Studies on Social Media: A Bibliometric Analysis. SAGE Open, 11(3), 21582440211047572. https://doi.org/10.1177/21582440211047572)

Wearn, A., & Shepherd, L. (2020). The impact of emotion-based mass media campaigns on stigma toward cervical screening non participation. Journal of Applied Social Psychology, 50(5), 289–298. https://doi.org/10.1111/jasp.12659

Yang, Y., & Hobbs, J. E. (2020). The power of stories: Narratives and information framing effects in science communication. American Journal of Agricultural Economics, 102(4), 1271–1296. https://doi.org/10.1002/ajae.12078

Ivan Lacasa-Mas

Reviewer #3 (Remarks to the Author):

Review for the paper 'The Rising Entropy of English in the Attention Economy'

This is an interesting and well-written paper with substantive content. Although there are a handful of passages which I found a bit unclear – see below – it is overall easy to follow. I find the data-driven approach and the results convincing overall. I found the part on information foraging more challenging to follow – there is an accumulation of layers which requires quite some time to assimilate.

I would recommend this paper to be published after some revisions.

There are several points which I think should be addressed to strengthen the paper:

First, in the whole paper, it is assumed that entropy estimates lexical diversity, and can be considered along other estimations such as the type-token ratio and Zipf exponent. You alternate in particular between the two concepts in the captions of the figures and elsewhere in the manuscript. However, I don't think the relationship is that straightforward - one can see for instance Thoiron 1986, "Diversity Index and Entropy as Measures of Lexical Richness", Computers & The Humanities 20: 197–202. There should be a thorough discussion about this since entropy is at the heart of your approach. This being said, I understand of course the point of having several complementary measurements of the information trends at stake. You could perhaps go further

when analyzing the similarities and differences between them. E.g., a and c seem much closer than they are to b. Also, around 1960, there seems to be a drop before a strong increase for all your three measures. Any idea what it could be?

Further with respect to entropy, you never discuss conditional entropy, although it would seem a better estimator of information than the non-conditional entropy you use, and some papers also investigating information in languages have relied on it. I would suggest you at least explain why non-conditional entropy is good enough, but getting results with let's say bigram entropy for the sake of comparison would make your case stronger. An issue with conditional entropy appears with very short-from media, because of the many transitions between the messages you assemble into 2000-word texts. But maybe this is still ok, and in any case, you can still work with the other corpora.

When you perform 24 trend tests for the COHA texts, their four categories and the three different measures adopted, you do not perform any adjustment for multiple testing. There should be some control of the increased rate of false positives here, for instance with Holm-Bonferroni correction if you want to control for the family-wise error rate. The same applies, although there are less tests, to the various ANOVA you rely on to investigate the differences between media categories. My intuition is that correcting the p-values will lead to some of the 24 tests not returning significant results anymore, but that doesn't seem to be a very big issue.

I am not sure your whole approach to 'information foraging in the attention economy' should be part of the 'Results' section of your paper. The point here is that you don't really use any of your initial data in this approach, but mostly provide an interpretative framework (which includes simulated data) for the (recent) trends you observe. As I am not familiar with information foraging (beyond the general idea of a modulation of selectivity in response to the environment), I found this part more challenging than the first one – at least at the level of the finer details. I also wonder whether it would be possible to go further and conceive of experiments with participants faced with different texts/sources of information. This could perhaps be interesting to point to, even if very briefly.

Regarding the discussion on the Linguistic Niche Hypothesis and the influence of second language learners, I think a more explicit historical/time perspective is missing: what is/are the relevant timescale(s) for morphological simplification and increase in the number of L2 speakers? The British Empire was at its height in the 19th and early 20th century. How did that translate in terms of number of L2 speakers? Was morphological simplification already at work then, or is it a more recent phenomenon?

What you write in the first part of the Supplementary Information could perhaps find its place in the main text to expand and better articulate the first part of the discussion. The content does not sound very 'supplementary information' to me, but I guess there is a constraint of space here.

Would you have any simple quantitative strategy to assess morphological complexity in your various texts that departs from lexical diversity and TTR – especially with the COHA corpus? The following paper would I think be a good starting point: Çağrı Çöltekin & Taraka Rama (2022). What do complexity measures measure? Correlating and validating corpus-based measures of morphological complexity. It would much strengthen your argumentation to have quantitative data for both entropy/lexical diversity and morphology and look for possible correlations (either positive or negative).

Other comments:

Line 41: How close are entropy and information density? What does specifically mean exactly here?

Lines 81-83: are we sure there are no elements at the end of the texts which can also cause problems? This is something to account for, for instance, in Project Gutenberg text files, but I guess not with your corpora.

Lines 116-118: I agree that the analysis is not flawed. I guess the question is mostly about how one can effectively measure the information density of a social media.

Lines 121-122: If we know the (shape of the) relationship between lexical measures and sample size, could we account for it? I guess it doesn't matter since all of your texts are of the same size, but maybe you could specify the shape of the relationship if it is known and stable. Also, is 2,000 words a small size given the mentioned sensitivity to size?

Lines 124-126: given the importance of the concept of entropy in your study, maybe you should write more about the plug-in estimator versus more advanced estimators.

Lines 123-126 and 1st formula: (Empirical unigram word) Entropy is based on the distribution of words in a given text. Yet, at least for the most recent time periods, one could consider the frequencies of words as assessed in much larger text corpora, and rely on these frequencies to compute the entropy of the target texts. Does that make sense? Given that experienced readers have been exposed to a very large number of texts, maybe at a cognitive level the word frequencies that matter are those derived from these many texts, and not those specific to a 2000-word text)? Would we get similar results with this approach? That seems interesting to investigate.

Lines 148-150: I am not entirely convinced the influence of outliers gets smaller with this approach, as the 'first level' of averaging is still sensitive to them, and then you only take the average of 4 categories, not 20 or 100.

Lines 189-192: Maybe explain what's the point of looking at this, especially since it only appears in the Supplementary Information

Lines 231-233: Shannon's idea here seems to refer to conditional entropy, no? ('given the preceding symbols'). You may want to be clearer here.

Line 233: entropy can be thought of as a rate of information: I think this deserves more explanations, as you assimilate a ‘static’ measure to a non-static / time-related one.

Lines 236-240: Maybe you could further explicitly report on possible additional factors guiding the selection of information in humans besides information density. You mention some factors at the very end of your discussion, but maybe this should come earlier and be expanded. Could you actually account for this in your model – which would have further novelty to your approach?

Line 338: maybe explain a bit more ‘cooling by expansion’

Lines 343-344: if people’s attention is attracted to high complexity information, why not be attracted to morphological complexity?...

Each time you mention elements in the Supplementary Information, maybe it would help to provide more specific references to the sections and figures?

In the Supplementary Information, lines 60-62: indeed Pearson’s r correlation does not seem appropriate here due to temporal autocorrelation. Not entirely sure, but maybe you could consider a generalized least squares model with a correlation structure like AR1?

Section 2 of the Supplementary Information could perhaps find its place in the paper too (unless you have a strong word limit), since it provides interesting information about how one can observe clear signals of economic transformations in the evolution of entropy.

Minor comments:

All p in ‘p-value(s)’ should appear in italic.

Line 109: Removed any urls -> Removed any url

Line 110: usernames i.e. any words containing usernames, i.e., any word containing

Line 127: by the total words -> by the total number of words

Line 133: widely used and well known -> well known and widely used

Line 134: comparision -> comparison

Line 141: These were plotted -> a bit unclear what ‘these’ refer to

Line 147: we first took means -> we first took the means (?)

Line 172: as well as the categories -> as well as for the categories

Lines 189-191: This sentence is not very clear (some data attributed to other data?)

Line 192: The data was plotted without further treatment -> which figure are you referring to here?

Caption of Figure 1: Shaded region shows 95% confidence interval of the average Shaded regions show 95% confidence intervals for the different averages (not sure, but since you have three panels and thus three confidence intervals.

Line 205: And -> and

Line 206: of trends at -> of a trend at (same as just before?)

Line 211: I am not sure it is the trend itself which suggests that.

Line 212: has become -> have become (?)

Line 218: ANOVA test $p < 0.01$ -> ANOVA tests $p < 0.01$

Line 219: short-form media categories -> the short-form media categories

Lines 221-226: you have already said that, maybe there’s no need to repeat it (and it will save some space for something else)? The last sentence is clear and impactful, though, I’d suggest you keep it somewhere.

Line 228: The results -> The previous results (?)

Lines 243-244: The derivation… -> unclear, maybe rephrase

Lines 333-334: of more difficult to process linguistic forms -> grammatical issue

Good luck and thanks for an interesting read.

Christophe Coupé (Department of Linguistics, The University of Hong Kong)

[Full text of my review in *.docx* format for convenience, in case it is less cumbersome than a simple text pasted in a text box.]

I have read the paper "The Rising Entropy of English in the Attention Economy" with great interest and I find it to be a well-written and thought-provoking contribution to the field of sociolinguistics. The study provides compelling evidence that word entropy in American English has been steadily increasing since around 1900, contrary to existing theories, and that this trend is driven by factors such as technological change and information foraging.

In the following paragraphs, I will provide a detailed analysis and comments on the three bis sections of the draft, along with a brief comment on the supplementary information provided by the authors and some last thoughts. My suggestions for improvement are relatively minor and should be considered as an opportunity to enhance an already sound study.

Materials and Methods (p. 2)

I would like to commend the authors on their thorough approach to data collection, cleaning, processing, and analysis. The rigorous treatment of data sets is evident in the methodologies outlined, which encompass a broad range of corpus data and social media inputs. Each step of the process is given due consideration and this commitment to precision and detail fosters confidence in the overall credibility of the research.

The choice of text corpora is diverse and well-reasoned, covering a wide temporal and geographical range and including different genres and categories. The measures taken to clean the text sample data were appropriate and methodical. Particularly, the application of python's natural language toolkit (nltk) package to convert text to tokens, and selection of the last 2000 tokens for processing demonstrates a thoughtful approach to text analysis. Also noteworthy is the authors' diligent application of data cleaning measures for the COHA, COCA, and BNC datasets. The consistent application of procedures across all data sources underpins the credibility of subsequent analyses and is a testament to the authors' commitment to scientific rigour.

The selection and treatment of social media data from Twitter and Reddit were initially deemed robust. The innovative collation strategy employed to simulate a social media feed appeared promising, providing a potential means to capture the richness of these data sources. The authors also demonstrated an awareness of the unique considerations associated with social media text, such as the cleaning process that accounted for URLs, hashtags, and usernames.

However, it has come to my attention that the collected tweets are from 2009, while the Reddit posts were collected in March 2023. This raises concerns for me as we could potentially be dealing with outdated (Twitter) or inaccurate data (Reddit). For instance, considering Figure 3, Reddit's top position in terms of Word Entropy, compared to Twitter, could arguably be

attributed to the timing of data collection. Notably, the most recent data analyzed in this draft are the Reddit posts. However, the data collection end period for Twitter, COHA, COCA, and BNC corpora would be at least from approximately 13-14 years ago. It is essential to consider that during this time, Twitter was only three years old (established in March 2006), and we are juxtaposing it with the data from a social platform that, having been established in June 2005, was already almost 18 years old in 2023. Both the technical options and the ethos of the platforms, users and content, may have significantly evolved during this period. This contrast in age and context between the Twitter data and the Reddit data should be taken into account, as it introduces a certain qualitative arbitrariness in the study's core. We will revisit this issue later in our final thoughts. For now, I would like to offer one more suggestion, namely, to include the date of the Reddit data collection in the first of the two Social Media Data subsections (p. 3), paralleling what has already been done with the Twitter data. I only found the Reddit's collection information in the Data Availability subsection (i.e., on p. 12).

Moving forward, the authors' choice of using unigram word entropy as a measure of information evolution is a solid choice. Additionally, their application of the maximum likelihood estimator, as well as their consideration of both the type token ratio and Zipf exponent as measures of lexical diversity, alludes to a comprehensive and meticulous approach towards understanding the nuances of the text.

In terms of timeseries analysis, the decision to apply both the KPSS and MK tests to explore trends in lexical measures over time is a sound approach, providing a well-rounded understanding of the data's underlying structures. I appreciate the careful choice of the KPSS test, which is particularly suited to detect stationarity in a time series, and the MK test, which is a reliable non-parametric trend test.

The employment of timeseries smoothing, using a moving average method, provides a clearer visual representation of the data and enhances the interpretability of the results. The authors' decision to only include years with at least 10 data points within the window and their meticulous calculation of the confidence interval, demonstrates a thoughtful consideration of statistical robustness.

The examination of differences between media categories using ANOVA tests also demonstrates a strong methodological approach. However, I would suggest that the authors provide a deeper explanation or justification for the choice of the Scott rule in the density estimate.

Finally, the use of magazine circulation data from the Audit Bureau of Circulation lends a degree of real-world grounding to the study. Nonetheless, the authors might want to consider discussing the limitations of this specific dataset and how these might impact their findings.

Overall, in this section the authors demonstrate, setting aside the aforementioned limitations or concerns for a moment, an impressive commitment to methodological rigor, which contributes to the robustness of their ulterior findings.

RESULTS (p. 5)

In my evaluation, the Results section of the paper is appropriately divided into relevant subsections, effectively addressing the key topics of interest. Each subsection serves a unique purpose and contributes to the overall understanding of the subject matter. Here, I discuss these subsections in greater detail, considering their rationale, correctness, and implications.

In the "Higher Entropy in Short-form Media" subsection, the authors argue that the historical trend implies modern differences in entropy between media categories. While they mention the prominence of short-form media in the digital landscape, the ideas could benefit from further elaboration. A more nuanced discussion around the role of attention spans in media consumption, and the impact of algorithms or video feeds, could provide a better-rounded perspective. By addressing some of these aspects, at least as recognized limitations, in the discussion section, the study can offer a more comprehensive analysis of the dynamics of social media consumption. This inclusion would acknowledge the potential impact of attention spans on learning new optimized terms or expressions, as well as the influence of algorithms in shaping content. Additionally, recognizing the growing significance of audiovisual materials on platforms like TikTok, with considerably fewer texts and a larger user base than Reddit, would provide valuable insights. By incorporating these limitations in a manner at the discretion of the authors, the discussion section would contribute to a deeper understanding of the study's findings in the context of contemporary trends in social media usage.

The "Information Foraging in the Attention Economy" subsection exhibits a laudable effort to draw a connection between the competition for attention and word entropy. The authors utilize mathematics to model the attention economy based on information foraging, effectively adapting concepts such as information signal entropy, forager utility, and least effort principle to serve their purpose. While they present a perfectly valid case in linking these concepts, it is crucial to acknowledge that the model might oversimplify human behavior and social complexities. Future studies could delve deeper by incorporating additional factors influencing information consumption patterns, such as psychological biases, cultural norms, or socio-economic background.

In the "Competition Between Media Platforms Drives Differences Between Short- and Long-form Media" subsection, the authors evoke foraging theory in the context of information consumption and media platforms to explain observed differences in information utility rates. I commend the authors for their efforts in discussing competition, information foraging, and attention economy concepts. While these notions are not entirely new, their combination and translation into clear and informative mathematical models in the paper are innovative, bridging the gap between theoretical concepts and quantitative analysis. However, the simplification of human and social reality should be kept in mind when interpreting and generalizing the results.

In summary, in this section the authors have done a laudable job of presenting a complex topic through a cohesive and coherent framework. Their work paves the way for future studies to address the limitations and expand upon the ideas introduced, ultimately enabling a more comprehensive and nuanced understanding of the connections between entropy, media types, information foraging, and the attention economy.

Discussion (p. 10)

The "Discussion" section of the paper provides fascinating insights and draws important contrasts with existing theories. However, the authors could improve upon certain aspects, particularly in granularity and by briefly referring to relevant interdisciplinary research.

While the attention economy model is introduced with strong rationale, connections to empirical results could be further elaborated. Cross-references to research fields such as Communication Research, which have investigated social media from quantitative and qualitative perspectives, would be beneficial to strengthen their argument and avoid potential oversimplifications.

The discussion on the Linguistic Niche Hypothesis and the evidence of morphological simplification in English is intriguing. Although the authors claim that the pressure towards information density overcomes reduced word entropy from linguistic simplification, a deeper exploration of cause-effect relationships or literature comparisons would provide a more solid foundation for their hypothesis.

As the authors delve into the psychological consequences of language evolution, they touch upon various principles governing language development from biological, cultural, learning, and other perspectives. Nonetheless, the connection to the research question, particularly to word entropy in the context of information abundance, could be more clearly articulated.

Figure 6 in the discussion adds a valuable visual illustration of the relation between minimum average information size and information prevalence. Expanding on the implications of this relationship and its relevance to the current state of digital media and communication would make the discussion more informative and engaging.

The comparison of human information foragers and empirical findings on the attraction to high entropy and complexity is particularly appealing. Notwithstanding, a broader analysis of the competition within the attention economy and media content adaptation for capturing limited attention may prove fruitful.

Lastly, the authors highlight that human choices are influenced by factors beyond entropy, such as social cues and risks. Nevertheless, the relevance of these factors to the present analysis is only briefly touched upon, without sufficiently illustrating their potential impact on language

evolution and cultural history. Delving deeper into the interplay between these factors and the theories put forth in the paper would provide a more comprehensive understanding of the subject matter.

In conclusion, the "Discussion" section showcases promising insights and thought-provoking contrasts with existing theories. However, enhancing granularity, connecting empirical results, and exploring interdisciplinary research could further strengthen the section and render a more profound contribution to the field.

Supplementary Information (Additional File)

The provision of supplementary materials alongside the manuscript is appreciated. The supplementary information has proven to be valuable, particularly in relation to sub-sections 3, 4, and 5. The inclusion of detailed information in these sections has enhanced the clarity and depth of the study.

However, it is worth noting that the presence of two subsections, specifically 7 and 8, containing only headers but no content, was surprising, or at least I haven't been able to find an explanation for this fact. If this oversight is on my part, I apologize in advance to the authors. It is unclear if any indications or justifications were provided by the authors to account for the inclusion of these empty subsections. If indeed both subsection titles are orphaned, i.e. if is ultimately determined that the inclusion of subsections 7 and 8 is not necessary, it would be advisable to remove them.

Final thoughts

In summary, I think the authors' meticulous approach to data preparation, underscored by strategic use of computational tools, meets high standards. Moreover, their transparency in delineating study procedures and protocols bolsters credibility and fosters potential replication by others in the field. Furthermore, they have done an excellent job presenting complex ideas clearly while still leaving plenty open-ended questions ripe for future research. Their work paves the way for future studies to address the limitations and expand upon the ideas introduced, ultimately enabling a more comprehensive and nuanced understanding of the connections between entropy, media types, information foraging, and the attention economy.

Regarding information foraging, it is important to consider a specific aspect that deserves attention. This concerns the use of animal models as a means of predicting human behavior in various domains. Although useful to gain insight into human decision making in specific situations it is crucial to approach these models with care and acknowledge their potential restrictions.Humans exhibit an intricate nature with advanced cognitive skills that exceed those seen in many animals. Our actions and behaviors arise out of multiple factors much more

complicated than the drive to satisfy our hunger (Ju et al., 2022). Even though animal foraging routes provide an effective starting point, expanding on this model by considering a more extensive array of components and possibilities becomes necessary when endeavoring to anticipate human conduct across various areas. Therefore, it is also important when writing, not to forget that a model is precisely that: a simplification of reality (Pourfattah et al., 2021). Undoubtedly, I concur with the view that the information foraging and attention competition models illuminate a significant aspect of human behaviour. However, in the discussion section, it might be beneficial to more explicitly address some inherent limitations or reservations linked with this approach.

Likewise, in wrapping up this review, it's necessary to recognise that the attention economy model, while insightful, may be somewhat reductive in its suggestion that human attention is primarily governed by the maximisation of novel information intake. Such a model may overlook the richness and variability of human motivation and behaviour. For instance, emotional engagement often dictates where our attention is directed (Saunders, 2020); a moving film or riveting book tends to seize our focus more readily than an informative brochure, illustrating the power of emotional narratives in various sectors, from entertainment to brand communication and news consumption (Yang and Hobbs, 2020; Wearn and Shepherd, 2020; Meuer et al., 2023).

Similarly, the influence of social connections on attention allocation is significant, as evidenced by the popularity of socially-oriented media over generic advertisements or updates (Baek and Parkinson, 2022). Information relevant to our social circle frequently captures our attention more than impersonal content. Moreover, elements such as emotional engagement, social connections, personal values, and attitudes each play a pivotal role in shaping decision-making processes and attention allocation, exhibiting complex dynamics rather than a strictly linear relationship, warranting further consideration (Lin et al., 2017; Ponizovskiy, 2020). Another intriguing research direction would involve exploring the impact of gender, education, income, cultural background, and political beliefs on information-foraging behaviors and responses to different media modalities.

In another final note on this review, it is worth mentioning that subsequent research would potentially be enriched by the incorporation of up-to-date data sourced from current, prominent social media platforms. Engaging with such data through the lens of cross-disciplinary approaches and mixed methods could further elucidate the nuanced dynamics of language usage on social media (Sun et al., 2021). Additionally, an interesting avenue for future research could be to discern whether these observed trends are exclusive to English, or if they transcend linguistic boundaries and are observable across a spectrum of other languages.

In conclusion, notwithstanding the discussions presented throughout this review and these final
r
e
f
l
e
c
t
i
o
n

potential limitations and broader societal implications would expand the reach of this crucial research area. The synthesis of big data and qualitative approaches mutually reinforce each other, combining conventional methods for contextual understanding with automated analysis of extensive text data, fostering an iterative process that both answers existing questions and generates new ones (Bail, 2014). This collaborative approach holds the potential to advance science, benefit society, and, importantly, positively impact individuals' lives.

Cited Literature

Baek, E. C., & Parkinson, C. (2022). Shared understanding and social connection: Integrating approaches from social psychology, social network analysis, and neuroscience. *Social and Personality Psychology Compass*, *16*(11), e12710. https://doi.org/10.1111/spc3.12710

Bail, C. A. (2014). The cultural environment: measuring culture with big data. *Theory and Society*, *43*(3–4), 465–482. https://doi.org/10.1007/s11186-014-9216-5

Ju, X., Martín Martín, O., & Chocarro, R. (2022). Determinants of mobile social media use, customer heterogeneity, and international microsegmentation. *International Journal of Consumer Studies*, *46*(4), 1127–1148. https://doi.org/10.1111/ijcs.12748

Lin, K.-Y., & Lu, H.-P. (2011). Why people use social networking sites: An empirical study integrating network externalities and motivation theory. *Computers in Human Behavior*, *27*(3), 1152–1161. https://doi.org/10.1016/j.chb.2010.12.009

Meuer, M., Oeberst, A., & Imhoff, R. (2022). How do conspiratorial explanations differ from non-conspiratorial explanations? A content analysis of real-world online articles. *European Journal of Social Psychology*, *53*(2), 288–306. https://doi.org/10.1002/ejsp.2903

Ponizovskiy, V., Ardag, M., Grigoryan, L., Boyd, R., Dobewall, H., & Holtz, P. (2020). Development and validation of the Personal Values Dictionary: A theory–driven tool for investigating references to basic human values in text. *European Journal of Personality*, *34*(5), 885–902. https://doi.org/10.1002/per.2294

Pourfattah, F., Wang, L.-P., Deng, W., Ma, Y.-F., Hu, L., & Yang, B. (2021). Challenges in simulating and modeling the airborne virus transmission: A state-of-the-art review. *Physics of Fluids*, *33*(10), 101302. https://doi.org/10.1063/5.0061469

Saunders, A. (2020). The eventisation of anniversary activity: Investing in 1989. *German Life and Letters*, *73*(3), 464–489. https://doi.org/10.1111/glal.12277

Sun, Y., Wang, G., & Feng, H. (2021). Linguistic Studies on Social Media: A Bibliometric Analysis. *SAGE Open*, *11*(3), 21582440211047572. https://doi.org/10.1177/21582440211047572)

Wearn, A., & Shepherd, L. (2020). The impact of emotion-based mass media campaigns on stigma toward cervical screening non participation. *Journal of Applied Social Psychology*, *50*(5), 289–298. https://doi.org/10.1111/jasp.12659

Yang, Y., & Hobbs, J. E. (2020). The power of stories: Narratives and information framing effects in science communication. *American Journal of Agricultural Economics*, *102*(4), 1271–1296. https://doi.org/10.1002/ajae.12078

Ivan Lacasa-Mas

Response to Reviewer Comments

We would like to thank both reviewers for their time, consideration and effort in reading the manuscript and providing constructive feedback.

Our responses to all comments, and details of changes to the manuscript, are in bold below.

Reviewer #1 (Remarks to the Author):

I have read the paper "The Rising Entropy of English in the Attention Economy" with great interest and I find it to be a well-written and thought-provoking contribution to the field of sociolinguistics. The study provides compelling evidence that word entropy in American English has been steadily increasing since around 1900, contrary to existing theories, and that this trend is driven by factors such as technological change and information foraging.

In the following paragraphs, I will provide a detailed analysis and comments on the three bis sections of the draft, along with a brief comment on the supplementary information provided by the authors and some last thoughts. My suggestions for improvement are relatively minor and should be considered as an opportunity to enhance an already sound study.

Thank you for the overall positive assessment on the paper.

Materials and Methods (p. 2)

I would like to commend the authors on their thorough approach to data collection, cleaning, processing, and analysis. The rigorous treatment of data sets is evident in the methodologies outlined, which encompass a broad range of corpus data and social media inputs. Each step of the process is given due consideration and this commitment to precision and detail fosters confidence in the overall credibility of the research.

The choice of text corpora is diverse and well-reasoned, covering a wide temporal and geographical range and including different genres and categories. The measures taken to clean the text sample data were appropriate and methodical. Particularly, the application of python's natural language toolkit (nltk) package to convert text to tokens, and selection of the last 2000 tokens for processing demonstrates a thoughtful approach to text analysis. Also noteworthy is the authors' diligent application of data cleaning measures for the COHA, COCA, and BNC datasets. The consistent application of procedures across all data sources underpins the credibility of subsequent analyses and is a testament to the authors' commitment to scientific rigour.

Thank you for the favourable evaluation of the statistical methods.

The selection and treatment of social media data from Twitter and Reddit were initially deemed robust. The innovative collation strategy employed to simulate a social media feed appeared promising, providing a potential means to capture the richness of these data sources. The authors also demonstrated an awareness of the unique considerations associated with social media text, such as the cleaning process that accounted for URLs, hashtags, and usernames.

However, it has come to my attention that the collected tweets are from 2009, while the Reddit posts were collected in March 2023. This raises concerns for me as we could potentially be dealing with outdated (Twitter) or inaccurate data (Reddit). For instance, considering Figure 3, Reddit's top position in terms of Word Entropy, compared to Twitter, could arguably be attributed to the timing of data collection. Notably, the most recent data analyzed in this draft are the Reddit posts. However, the data collection end period for Twitter, COHA, COCA, and BNC corpora would be at least from approximately 13-14 years ago. It is essential to consider that during this time, Twitter was only three years old (established in March 2006), and we are juxtaposing it with the data from a social platform that, having been established in June 2005, was already almost 18 years old in 2023. Both the technical options and the ethos of the platforms, users and content, may have significantly evolved during this period. This contrast in age and context between the Twitter data and the Reddit data should be taken into account, as it introduces a certain qualitative arbitrariness in the study's core. We will revisit this issue later in our final thoughts. For now, I would like to offer one more suggestion, namely, to include the date of the Reddit data collection in the first of the two Social Media Data subsections (p. 3), paralleling what has already been done with the Twitter data. I only found the Reddit's collection information in the Data Availability subsection (i.e., on p. 12).

Thank you for pointing this out. We agree and recognise the need to replicate the results with more recent Twitter data. We have now taken steps to more clearly highlight the date that the social media datasets were collected and include new analyses.

- ***We replicated the Twitter analysis with a dataset from 2020, and included that analysis along with the Twitter 2009 analysis in the main paper in Figure 3.***
- ***We also labeled the date that each of the social media datasets were collected (Twitter 2009, Twitter 2020, Reddit 2024) in Figure 3.***
- ***We included the date that the Reddit data was collected in the Social Media Data subsection in the Materials and Methods section.***
- ***We added a section to describe the Twitter 2020 dataset in Materials and Methods (Page 2):***
  "The Twitter 2020 dataset contained 16 million unique tweets collected from the Twitter API between February 2020 and January 2021. This data was originally downloaded and used in a study on political polarisation (Zhao et al, 2023), with tweets selected from followers of well-known US news sources (full details of data collection is in the original paper)."

Moving forward, the authors' choice of using unigram word entropy as a measure of information evolution is a solid choice. Additionally, their application of the maximum likelihood estimator, as well as their consideration of both the type token ratio and Zipf exponent as measures of lexical diversity, alludes to a comprehensive and meticulous approach towards understanding the nuances of the text.

In terms of timeseries analysis, the decision to apply both the KPSS and MK tests to explore trends in lexical measures over time is a sound approach, providing a well-rounded understanding of the data's underlying structures. I appreciate the careful choice of the KPSS test, which is particularly suited to detect stationarity in a time series, and the MK test, which is a reliable non-parametric trend test.

The employment of timeseries smoothing, using a moving average method, provides a clearer visual representation of the data and enhances the interpretability of the results. The authors' decision to only include years with at least 10 data points within the window and their meticulous calculation of the confidence interval, demonstrates a thoughtful consideration of statistical robustness.

Thank you for these positive comments.

The examination of differences between media categories using ANOVA tests also demonstrates a strong methodological approach. However, I would suggest that the authors provide a deeper explanation or justification for the choice of the Scott rule in the density estimate.

Thank you for highlighting this.

To construct the kernel density estimate we wanted to impose as little of our own prejudices on the data as possible, and so we used the default bandwidth selection rule in the plotting library (seaborn in python). We were aware that this was an adaptive bandwidth selection rule that is widely used and provides a reasonable and accessible visualisation of the distribution of the data.

After some research, we did not find very good justification for Scott's rule. There is also some confusion about whether Scott's rule is actually implemented in the python seaborn library in the same way as it was defined in Scott's paper (e.g. https://stats.stackexchange.com/questions/90656/kernel-bandwidth-scotts-vs-silvermans-rules). It looks like the Scott rule might actually be using a variation of the Silverman rule when specified in the seaborn visualisation library.

After investigating further, we decided to use the Silverman rule. This seems to be more widely used, more justifiable, and meets our requirements of being simple, well-known and robust.

We changed the code and visualisation (we should note that the impact on the visualisation was minimal) and we updated the Materials and Methods section.

We updated the Materials and Methods (Page 4):
"For visualisation, the distributions of word entropy for each media category are shown as a kernel density estimate with the bandwidth determined by the Silverman rule (Silverman, 1986). The Silverman rule is a relatively simple, well-known and robust heuristic that performs well over mild assumptions (data is unimodal, approximately symmetric and not heavy-tailed) (Heidenreich et al, 2013)."

Finally, the use of magazine circulation data from the Audit Bureau of Circulation lends a degree of real-world grounding to the study. Nonetheless, the authors might want to consider discussing the limitations of this specific dataset and how these might impact their findings.

Thank you for pointing this out. We added a paragraph to discuss the limitations of this dataset in the Discussion. Specifically, we pointed out that this data source includes only a subset of magazines and is not an exact measure of the entire magazine circulation.

We added to the Limitations (Page 12):
"In the Supplementary Information: Historical Analysis of US Magazine Publishing, we more closely investigated the US magazine industry, finding that changes in word entropy tend to follow changes in the magazine industry towards increasing competition for attention. As a part of this analysis we compared increasing word entropy to magazine circulation as measured by the Audit Bureau of Circulation, which was created in 1914 specifically to verify magazine circulation data for the purposes of advertisements (Tucker et al, 2013). There are some limitations to this data source as it does not track all magazine sales, only the most popular magazines (Sumner, 2010), i.e. the data is not a precise measure of all magazine circulation. However, the overall trend of increasing magazine circulation is well supported by other historical analyses (Tucker et al, 2013)."

Overall, in this section the authors demonstrate, setting aside the aforementioned limitations or concerns for a moment, an impressive commitment to methodological rigor, which contributes to the robustness of their ulterior findings.

RESULTS (p. 5)

In my evaluation, the Results section of the paper is appropriately divided into relevant subsections, effectively addressing the key topics of interest. Each subsection serves a unique purpose and contributes to the overall understanding of the subject matter. Here, I discuss these subsections in greater detail, considering their rationale, correctness, and implications.

In the "Higher Entropy in Short-form Media" subsection, the authors argue that the historical trend implies modern differences in entropy between media categories. While they mention the

prominence of short-form media in the digital landscape, the ideas could benefit from further elaboration. A more nuanced discussion around the role of attention spans in media consumption, and the impact of algorithms or video feeds, could provide a better-rounded perspective. By addressing some of these aspects, at least as recognized limitations, in the discussion section, the study can offer a more comprehensive analysis of the dynamics of social media consumption. This inclusion would acknowledge the potential impact of attention spans on learning new optimized terms or expressions, as well as the influence of algorithms in shaping content. Additionally, recognizing the growing significance of audiovisual materials on platforms like TikTok, with considerably fewer texts and a larger user base than Reddit, would provide valuable insights. By incorporating these limitations in a manner at the discretion of the authors, the discussion section would contribute to a deeper understanding of the study's findings in the context of contemporary trends in social media usage.

Thank you for highlighting this. We agree that these are important aspects to think about when discussing changes in media consumption, and we welcome the opportunity to connect these to our study. We added several paragraphs in the Discussion that discuss multimedia competition for attention, the rise of social media including the influence of algorithms, and changes in attention spans.

In regards to algorithms, we added to the Discussion (page 10):
“Social media further amplifies the relevance of this information by using algorithms that serve highly personalised feeds (Guinaudeau et al, 2022; Hazelwood et al, 2018; Huszar et al, 2022; Swart, 2021), potentially interacting with feelings of social connection and shared reality (Baek & Parkinson , 2022). This in turn has an effect on what media is produced, with creators and influencers tailoring their content to get picked up by the algorithms (Guinaudeau et al, 2022).”

In regards to audiovisual information and platforms like TikTok, we added this paragraph to the Limitations (page 13):
“Our study is limited to analysing text. Media is not limited in the same way and competition for human attention is played out across a multimedia landscape. There are well established differences in the way that people process information across modalities (Penny, 1989; Paivio, 1969; Weldon & Roediger, 1987), which suggest partially independent attention and processing mechanisms for audio and visual information (Mousavi et al, 1995; Paivio, 1990; Baddeley, 1992). Notably, multimedia information (e.g. text and images) can in some circumstances be processed more effectively than when presented in a single modality \(Mousavi et al, 1995; Childers & Houston, 1984; Penney, 1989). The idea of multimedia competition for attention is particularly relevant to modern media with rapidly growing audiovisual platforms such as TikTok (Guinaudeau et al, 2022). While focusing solely on text is limited, it does provide a useful framework for historical comparison, as well as the availability of well established data, tools and methodologies for analysis. Beyond text we also see potential trends of

increasing entropy in video with historical declines in shot length (time between the camera switching) (Mark, 2023) in film (Cutting et al, 2011), television (Butler, 2014) and advertisements (Maclachlan, 1993).”

In regards to attention spans, we added this paragraph to the Limitations (page 13):
The attention economy also seems to be changing how people engage with information. A review of studies between 2004 and 2021 found shortening attention spans, in terms of time spent before switching media (Mark, 2023). This may be due to behavioural adaptations to efficiently process information in a media-rich world, or it may represent fundamental changes to cognitive systems. There are suggestions that screen media use in childhood can influence development towards lower attention spans and faster switching between media (Hayles, 2007)}, as well as rates of attention deficit hyperactivity disorder (Haylesm 2007; Nikkelen, 2014). However the nature of any associations, and their causality, is debated (Beyens et al, 2018). We hope that our model can contribute to this important debate.”

The "Information Foraging in the Attention Economy" subsection exhibits a laudable effort to draw a connection between the competition for attention and word entropy. The authors utilize mathematics to model the attention economy based on information foraging, effectively adapting concepts such as information signal entropy, forager utility, and least effort principle to serve their purpose. While they present a perfectly valid case in linking these concepts, it is crucial to acknowledge that the model might oversimplify human behavior and social complexities. Future studies could delve deeper by incorporating additional factors influencing information consumption patterns, such as psychological biases, cultural norms, or socio-economic background.

Thank you for pointing this out. We would very much welcome future studies to explore other factors and social complexities. We added a paragraph in the Limitations section to discuss the assumptions in the model and to highlight that this is an oversimplification of human behaviour. We suggested future work to investigate the influence of psychological biases, norms, learning, beliefs, education, gender and socio-economic background.

Added to the Limitations (page 13):
“. . . we recognise that human behaviour is in general more complex than animal behaviour, with more complicated goals and strategies. In addition, individual heterogeneity in e.g. values and motivations, can interact in complex ways with how people produce (Ponizovskiy et al, 2020) and consume (Ju et al, 2022) information. Humans also learn and are influenced by culture, which is particularly relevant in the realm of information search. In light of this, while information foraging models can be useful, we recognise the limitation of using such simple models to describe human behaviour. Future studies could explore the influence

of human biases, cultural norms and learning, as well as heterogeneity in human behaviour associated with e.g. political beliefs, education, gender, and socio-economic background. A clear step would be expanding these research questions to languages beyond English.”

In the "Competition Between Media Platforms Drives Differences Between Short- and Long-form Media" subsection, the authors evoke foraging theory in the context of information consumption and media platforms to explain observed differences in information utility rates. I commend the authors for their efforts in discussing competition, information foraging, and attention economy concepts. While these notions are not entirely new, their combination and translation into clear and informative mathematical models in the paper are innovative, bridging the gap between theoretical concepts and quantitative analysis. However, the simplification of human and social reality should be kept in mind when interpreting and generalizing the results.

Thank you. We added a note in the Limitations section about the complexities of society and the attention economy. We urged caution when interpreting and generalising our results.

We added this paragraph to the Limitations (page 12):
“The attention economy is a well established concept that recognises that information producers must compete for attention (Evans, 2020; Simon, 1969; Goldhaber, 1997). This apparently simple mechanism obscures the vast machinery of modern media to capture attention as quantified by clicks and likes (Terranova, 2012). In order to decide what content to serve, user behaviour data is fed into optimisation algorithms that range from A/B testing (Siroker & Kooman, 2015) to machine learning models (Hazelwood et al, 2018). To add to the complexity, salient topics rise and fall as the news cycle progresses. And there is evidence that this cycle itself is speeding up with shortening collective attention spans linked to attention economy pressures (Lorenz-Spreen et al, 2019). In addition, an abundance of information can lead to \emph{information overload} where individuals are unable to efficiently use the information available to them (Bawden & Robinson, 2008). Overall the attention economy is a complex and multi-faceted system. The full intricacies of this system are beyond the scope of this study, and we urge caution when interpreting and generalising the results that we present here. We hope that our study inspires further research to investigate the interactions between media and human behaviour.”

Added to the Limitations (page 11):
“The model presented here is a simplification of the real-world dynamics, which include complex interactions between human behaviour, economics, technology, and culture.”

In summary, in this section the authors have done a laudable job of presenting a complex topic through a cohesive and coherent framework. Their work paves the way for future studies to

address the limitations and expand upon the ideas introduced, ultimately enabling a more comprehensive and nuanced understanding of the connections between entropy, media types, information foraging, and the attention economy.

Discussion (p. 10)

The "Discussion" section of the paper provides fascinating insights and draws important contrasts with existing theories. However, the authors could improve upon certain aspects, particularly in granularity and by briefly referring to relevant interdisciplinary research.

While the attention economy model is introduced with strong rationale, connections to empirical results could be further elaborated. Cross-references to research fields such as Communication Research, which have investigated social media from quantitative and qualitative perspectives, would be beneficial to strengthen their argument and avoid potential oversimplifications.

> ***Thank you. We have included a much more detailed discussion of the model's assumptions and the attention economy in the Limitations section of the Discussion. This included cross-references to work in various fields, including social media analyses.***
>
> ***For example, we added to the Limitations (page 11):***
> ***"More generally, we note that big data corpus analysis is not a replacement for traditional cultural sociology techniques, but a complement (Janicke et al, 2015; Bail, 2014) and we encourage future work along more traditional qualitative lines (Sun et al, 2021)."***
>
> **And we added to the Discussion (page 10):**
> ***"Social media further amplifies the relevance of this information by using algorithms that serve highly personalised feeds (Guinaudeau et al, 2022; Hazelwood et al, 2018; Huszar et al, 2022; Swart, 2021), potentially interacting with feelings of social connection and shared reality (Baek & Parkinson , 2022). This in turn has an effect on what media is produced, with creators and influencers tailoring their content to get picked up by the algorithms (Guinaudeau et al, 2022)."***

The discussion on the Linguistic Niche Hypothesis and the evidence of morphological simplification in English is intriguing. Although the authors claim that the pressure towards information density overcomes reduced word entropy from linguistic simplification, a deeper exploration of cause-effect relationships or literature comparisons would provide a more solid foundation for their hypothesis.

Thank you. We have thought deeply about this comment, and the comparison to the Linguistic Niche Hypothesis. In particular, we recognised the difficulty in trying to describe a complex process of linguistic change through the lens of simultaneous pressures for morphological simplification and higher entropy, and we recognise that this is not a simple analysis. For this reason we have decided to scale back the ambition in drawing direct arguments against the linguistic niche hypothesis. We feel that the article is stronger by being more focussed on what we did find, i.e. an increase in entropy, and leaving open the more speculative question of how this interacts with other forms of linguistic pressures.

Along these lines, we added a paragraph in the Discussion to explore the potential implications to the linguistic niche hypothesis, but we have removed the SI material on the Linguistic Niche Hypothesis.

Added to the Discussion (page 10):
Our findings offer an interesting additional perspective on pressures involved in linguistic evolution. For example, the Linguistic Niche Hypothesis (Lupyan & Dale, 2010) predicts a loss of complex morphological forms in English due to the influence of second language learners. Indeed, there is ample evidence that English is undergoing morphological simplification (Michel et al, 2011; Lieberman et al, 2007; Zhu & Lei, 2018). If we can summarise the effect of the Linguistic Niche Hypothesis as a pressure towards learnability, then our study claims an additional pressure towards expressivity. One might expect these two dynamics to be in opposition, as simpler symbolic rules would a priori be less expressive per symbol and therefore have lower entropy (Mackay, 2003). However, a more nuanced analysis can take into account the fact that human language includes redundancy (Shannon, 2951), in which case a pressure towards learnability through simplification by reduction of this redundancy would simultaneously increase entropy. While we can speculate about the interaction of these pressures (and other factors), we encourage further work in this area."

As the authors delve into the psychological consequences of language evolution, they touch upon various principles governing language development from biological, cultural, learning, and other perspectives. Nonetheless, the connection to the research question, particularly to word entropy in the context of information abundance, could be more clearly articulated.

Thank you. Our intention here is to point to other research that connects changes in language to social behaviour. We changed the opening and final sentence of that paragraph to connect this discussion of other work more closely to our model.

Discussion now reads (page 10):
"Our model connects changes in language to social behaviour. Previous work along these lines has shown language evolution to follow a number of principles governed by human psychology. These principles have, for example, included

features of biological and cultural evolution (Smith & Kirby, 2008; Christiansen & Chater, 2008), learning (Christiansen & Chater, 2008; Hills & Adelman, 2015; Lupyan & Dale, 2010), word formation and distribution (Ferrer i Cancho & Sole, 2003; Petersen et al, 2012; Li et al, 2024), and the decay of morphological complexity (Lupyan & Dale, 2010; Lieberman et al, 2007). We add to this list and claim that word entropy rises in response to information abundance."

Figure 6 in the discussion adds a valuable visual illustration of the relation between minimum average information size and information prevalence. Expanding on the implications of this relationship and its relevance to the current state of digital media and communication would make the discussion more informative and engaging.

Thank you for pointing that out. We added a paragraph in the Discussion to discuss the implication that social media becomes viable in a world of short-form messages. We connected this with trends in modern social media with personalised news feeds and autoloading content.

Added to the Discussion (page 10):
"An unexpected consequence of our model is that very short-form media becomes viable when people are able to switch to new pieces of information quickly, with a relationship between minimum average information size and information prevalence (Figure 6). This is framed in our model as increasing the platform information density by reducing information search costs and simultaneously increasing entropy through rapid switching of contexts between short information items. Social media further amplifies the relevance of this information by using algorithms that serve highly personalised feeds (Guinaudeau et al, 2022; Hazelwood et al, 2018; Huszar et al, 2022; Swart, 2021), potentially interacting with feelings of social connection and shared reality (Baek & Parkinson , 2022). This in turn has an effect on what media is produced, with creators and influencers tailoring their content to get picked up by the algorithms (Guinaudeau et al, 2022)."

The comparison of human information foragers and empirical findings on the attraction to high entropy and complexity is particularly appealing. Notwithstanding, a broader analysis of the competition within the attention economy and media content adaptation for capturing limited attention may prove fruitful.

We added a paragraph in the Limitations section to discuss existing studies that describe attention economy pressures and how that connects to economic models of the media and competition for attention and clicks.

We added this to the Limitations (page 12). (We also presented this passage in reference to a comment above).
"The attention economy is a well established concept that recognises that information producers must compete for attention (Evans, 2020; Simon, 1969; Goldhaber, 1997). This apparently simple mechanism obscures the vast machinery of modern media to capture attention as quantified by clicks and likes (Terranova, 2012). In order to decide what content to serve, user behaviour data is fed into optimisation algorithms that range from A/B testing (Siroker & Kooman, 2015) to machine learning models (Hazelwood et al, 2018). To add to the complexity, salient topics rise and fall as the news cycle progresses. And there is evidence that this cycle itself is speeding up with shortening collective attention spans linked to attention economy pressures (Lorenz-Spreen et al, 2019). In addition, an abundance of information can lead to \emph{information overload} where individuals are unable to efficiently use the information available to them (Bawden & Robinson, 2008). Overall the attention economy is a complex and multi-faceted system. The full intricacies of this system are beyond the scope of this study, and we urge caution when interpreting and generalising the results that we present here. We hope that our study inspires further research to investigate the interactions between media and human behaviour."

Lastly, the authors highlight that human choices are influenced by factors beyond entropy, such as social cues and risks. Nevertheless, the relevance of these factors to the present analysis is only briefly touched upon, without sufficiently illustrating their potential impact on language evolution and cultural history. Delving deeper into the interplay between these factors and the theories put forth in the paper would provide a more comprehensive understanding of the subject matter.

Thank you. We added a paragraph in the Limitations that discusses other factors that influence human attention. We suggested future work that could look at how these factors can combine.

Added to the Limitations (page 13):
"Beyond information density (Itti & Baldi, 2009; Radach, 2003), there is evidence that human attention is also attracted to information that is belief-consistent (Hills, 2019; Taber & Lodge, 2006; Hart et al, 2009), predictive (Hills, 2019), social (Hills, 2019; Davis & McLeod, 2003), negative (Hills, 2019; Davis & McLeod, 2003, Lang et al, 1990) and emotive (Vuilleumier, 2005; Anderson, 2005; Saunders, 2020). Our model does not account for these other dimensions, and instead we make the assumption that aggregate behaviour can be usefully understood by considering the dimension of information density in isolation. A similar assumption is made in food foraging models, which usually focus on net calorie intake (Stephens & Krebs, 1986) while omitting other important factors such as macronutrient content (Hill, 1988) and predation risk (Verdolin, 2006). However, just as food foraging models have their limitations in capturing the multi-faceted nature of foraging, so does our model in capturing the intricacies of information search. Future work

could explore how various factors, including word entropy, attract human attention. This could extend to considering how different factors might interact. For example, the extremely attention grabbing utterance "FIRE!" is at once concise, predictive, negative and emotive.”

In conclusion, the "Discussion" section showcases promising insights and thought-provoking contrasts with existing theories. However, enhancing granularity, connecting empirical results, and exploring interdisciplinary research could further strengthen the section and render a more profound contribution to the field.

Supplementary Information (Additional File)

The provision of supplementary materials alongside the manuscript is appreciated. The supplementary information has proven to be valuable, particularly in relation to sub-sections 3, 4, and 5. The inclusion of detailed information in these sections has enhanced the clarity and depth of the study.

However, it is worth noting that the presence of two subsections, specifically 7 and 8, containing only headers but no content, was surprising, or at least I haven't been able to find an explanation for this fact. If this oversight is on my part, I apologize in advance to the authors. It is unclear if any indications or justifications were provided by the authors to account for the inclusion of these empty subsections. If indeed both subsection titles are orphaned, i.e. if is ultimately determined that the inclusion of subsections 7 and 8 is not necessary, it would be advisable to remove them.

Thank you for pointing this out. This was partly a formatting problem. We appreciate that it was unclear. We moved these to subsections under the section “Extended Data”. We also added text content under the subsection headings that refer to the figures in question. We also made some formatting changes to latex to ensure that the figures are shown in the relevant positions and it is clearer to the reader.

Final thoughts

In summary, I think the authors’ meticulous approach to data preparation, underscored by strategic use of computational tools, meets high standards. Moreover, their transparency in delineating study procedures and protocols bolsters credibility and fosters potential replication by others in the field. Furthermore, they have done an excellent job presenting complex ideas clearly while still leaving plenty open-ended questions ripe for future research. Their work paves the way for future studies to address the limitations and expand upon the ideas introduced, ultimately enabling a more comprehensive and nuanced understanding of the connections between entropy, media types, information foraging, and the attention economy.

Regarding information foraging, it is important to consider a specific aspect that deserves attention. This concerns the use of animal models as a means of predicting human behavior in

various domains. Although useful to gain insight into human decision making in specific situations it is crucial to approach these models with care and acknowledge their potential restrictions.Humans exhibit an intricate nature with advanced cognitive skills that exceed those seen in many animals. Our actions and behaviors arise out of multiple factors much more complicated than the drive to satisfy our hunger (Ju et al., 2022). Even though animal foraging routes provide an effective starting point, expanding on this model by considering a more extensive array of components and possibilities becomes necessary when endeavoring to anticipate human conduct across various areas. Therefore, it is also important when writing, not to forget that a model is precisely that: a simplification of reality (Pourfattah et al., 2021). Undoubtedly, I concur with the view that the information foraging and attention competition models illuminate a significant aspect of human behaviour. However, in the discussion section, it might be beneficial to more explicitly address some inherent limitations or reservations linked with this approach.

> **We have added a paragraph in the Limitations section to address the use of animal models to describe human behaviour, and explore how human behaviour is more complex in important ways including more advanced cognition and learning behaviour.**
>
> **Thank you for the references to papers. In particular, we found the Ju et al paper to be extremely relevant to our paper, and we cited this in that paragraph.**
>
> **Added to the Limitations (page 13):**
> ***". . . we recognise that human behaviour is in general more complex than animal behaviour, with more complicated goals and strategies. In addition, individual heterogeneity in e.g. values and motivations, can interact in complex ways with how people produce (Ponizovskiy et al, 2020) and consume (Ju et al, 2022) information. Humans also learn and are influenced by culture, which is particularly relevant in the realm of information search. In light of this, while information foraging models can be useful, we recognise the limitation of using such simple models to describe human behaviour. Future studies could explore the influence of human biases, cultural norms and learning, as well as heterogeneity in human behaviour associated with e.g. political beliefs, education, gender, and socio-economic background. A clear step would be expanding these research questions to languages beyond English."***

Likewise, in wrapping up this review, it's necessary to recognise that the attention economy model, while insightful, may be somewhat reductive in its suggestion that human attention is primarily governed by the maximisation of novel information intake. Such a model may overlook the richness and variability of human motivation and behaviour. For instance, emotional engagement often dictates where our attention is directed (Saunders, 2020); a moving film or riveting book tends to seize our focus more readily than an informative brochure, illustrating the power of emotional narratives in various sectors, from entertainment to brand communication

and news consumption (Yang and Hobbs, 2020; Wearn and Shepherd, 2020; Meuer et al., 2023).

Thank you. We noted that human attention can be attracted to other factors in the Limitations section.

We enjoyed and cited the papers by Saunders, Meuer et al, and Yang and Hobbs.

We mentioned how human behaviour is also influenced by emotions and narratives (page 13):
"Beyond attention, we can ask how beliefs are influenced through factors such as emotive (Wearn & Shepherd, 2020) or narrative (Yang & Hobbs, 2020) descriptions."

Similarly, the influence of social connections on attention allocation is significant, as evidenced by the popularity of socially-oriented media over generic advertisements or updates (Baek and Parkinson, 2022). Information relevant to our social circle frequently captures our attention more than impersonal content.

Thank you. We enjoyed the paper by Baek and Parkinson, and their perspective that perceptions of shared reality with others can facilitate a homophily in mental processing in people with close social connections. We added a reference to this in the Discussion.

More generally, we also noted that social information is attractive to human attention in the Limitations section.

Moreover, elements such as emotional engagement, social connections, personal values, and attitudes each play a pivotal role in shaping decision-making processes and attention allocation, exhibiting complex dynamics rather than a strictly linear relationship, warranting further consideration (Lin et al., 2017; Ponizovskiy, 2020).

We agree this is complex. We discussed these elements and the complexity at play in decision-making and attention in the Limitations. We enjoyed the papers by Lin et al and Ponizovskiy et al, which we also cited.

Added to the Limitations (page 13):
". . . individual heterogeneity in e.g. values and motivations, can interact in complex ways with how people produce (Ponizovskiy et al, 2020) and consume (Ju et al, 2022) information."

Another intriguing research direction would involve exploring the impact of gender, education, income, cultural background, and political beliefs on information-foraging behaviors and responses to different media modalities.

In the Limitations section, we suggested future work could look into these kinds of factors.

Added to the Limitations (page 12):
"Future studies could explore the influence of human biases, cultural norms and learning, as well as heterogeneity in human behaviour associated with e.g. political beliefs, education, gender, and socio-economic background. A clear step would be expanding these research questions to languages beyond English."

In another final note on this review, it is worth mentioning that subsequent research would potentially be enriched by the incorporation of up-to-date data sourced from current, prominent social media platforms. Engaging with such data through the lens of cross-disciplinary approaches and mixed methods could further elucidate the nuanced dynamics of language usage on social media (Sun et al., 2021).

Thank you. We added a suggestion in the Limitations section for the use of up-to-date data from prominent social networks.

Added to the Limitations (page 13):
"Beyond experiments, the use of up-to-date real-world data from prominent social media sites represents a significant opportunity for insights into what drives engagement. Such datasets are undoubtedly rich and offer many avenues for analysis including mixed methods approaches. There is sometimes a challenge in terms of access to such datasets, as such data can offer a competitive advantage to private social networks to improve engagement through their own private research efforts."

We found the paper by Sun et al to be a valuable resource to recognise the position of the current study more broadly within linguistics research. We added a reference to this paper. We also noted the value of qualitative approaches.

We added to the Limitations (page 11):
"More generally, we note that big data corpus analysis is not a replacement for traditional cultural sociology techniques, but a complement (Janicke et al, 2015; Bail, 2014) and we encourage future work along more traditional qualitative lines (Sun et al, 2021)."

Additionally, an interesting avenue for future research could be to discern whether these observed trends are exclusive to English, or if they transcend linguistic boundaries and are observable across a spectrum of other languages.

We added a note in the Limitations section to call for expanding this research to other languages.
Added to the Limitations (page 13):

"Future studies could explore the influence of human biases, cultural norms and learning, as well as heterogeneity in human behaviour associated with e.g. political beliefs, education, gender, and socio-economic background. A clear step would be expanding these research questions to languages beyond English."

In conclusion, notwithstanding the discussions presented throughout this review and these final reflections, at its core, the study " The Rising Entropy of English in the Attention Economy " makes a substantial contribution to our understanding of language evolution within the context of the attention economy. While it provides valuable insights into growing entropy and its implications, further clarification regarding the gap between the Twitter and Reddit data, along with addressing the points highlighted in this review, would enhance its impact. Additionally, explicit consideration of potential limitations and broader societal implications would expand the reach of this crucial research area. The synthesis of big data and qualitative approaches mutually reinforce each other, combining conventional methods for contextual understanding with automated analysis of extensive text data, fostering an iterative process that both answers existing questions and generates new ones (Bail, 2014). This collaborative approach holds the potential to advance science, benefit society, and, importantly, positively impact individuals' lives.

Thank you for the kind words. We very much appreciate your comments in this review and have endeavoured to incorporate the feedback into the article.

We noted the importance of combining big data analyses with more traditional social science in the Discussion section. Thank you for highlighting the paper by Bail, which provides valuable insight into the different research priorities of traditional sociologists and computer scientists. We explored this idea further and included a paragraph in the Discussion, which also cites Bail.

Added to the Limitations (page 11):
"More generally, we note that big data corpus analysis is not a replacement for traditional cultural sociology techniques, but a complement (Janicke et al, 2015; Bail, 2014) and we encourage future work along more traditional qualitative lines (Sun et al, 2021)."

Cited Literature

Baek, E. C., & Parkinson, C. (2022). Shared understanding and social connection: Integrating approaches from social psychology, social network analysis, and neuroscience. Social and Personality Psychology Compass, 16(11), e12710. https://doi.org/10.1111/spc3.12710

Bail, C. A. (2014). The cultural environment: measuring culture with big data. Theory and Society, 43(3–4), 465–482. https://doi.org/10.1007/s11186-014-9216-5

Ju, X., Martín Martín, O., & Chocarro, R. (2022). Determinants of mobile social media use, customer heterogeneity, and international microsegmentation. International Journal of Consumer Studies, 46(4), 1127–1148. https://doi.org/10.1111/ijcs.12748

Lin, K.-Y., & Lu, H.-P. (2011). Why people use social networking sites: An empirical study integrating network externalities and motivation theory. Computers in Human Behavior, 27(3), 1152–1161. https://doi.org/10.1016/j.chb.2010.12.009

Meuer, M., Oeberst, A., & Imhoff, R. (2022). How do conspiratorial explanations differ from non‐conspiratorial explanations? A content analysis of real‐world online articles. European Journal of Social Psychology, 53(2), 288–306. https://doi.org/10.1002/ejsp.2903

Ponizovskiy, V., Ardag, M., Grigoryan, L., Boyd, R., Dobewall, H., & Holtz, P. (2020). Development and validation of the Personal Values Dictionary: A theory–driven tool for investigating references to basic human values in text. European Journal of Personality, 34(5), 885–902. https://doi.org/10.1002/per.2294

Pourfattah, F., Wang, L.-P., Deng, W., Ma, Y.-F., Hu, L., & Yang, B. (2021). Challenges in simulating and modeling the airborne virus transmission: A state-of-the-art review. Physics of Fluids, 33(10), 101302. https://doi.org/10.1063/5.0061469

Saunders, A. (2020). The eventisation of anniversary activity: Investing in 1989. German Life and Letters, 73(3), 464–489. https://doi.org/10.1111/glal.12277

Sun, Y., Wang, G., & Feng, H. (2021). Linguistic Studies on Social Media: A Bibliometric Analysis. SAGE Open, 11(3), 21582440211047572. https://doi.org/10.1177/21582440211047572)

Wearn, A., & Shepherd, L. (2020). The impact of emotion‐based mass media campaigns on stigma toward cervical screening non

participation. Journal of Applied Social Psychology, 50(5), 289–298. https://doi.org/10.1111/jasp.12659

Yang, Y., & Hobbs, J. E. (2020). The power of stories: Narratives and information framing effects in science communication. American Journal of Agricultural Economics, 102(4), 1271–1296. https://doi.org/10.1002/ajae.12078

Thank you for referencing this literature. We found the papers very relevant to the study and we feel that the article was enriched through the addition of the research in these papers.

Ivan Lacasa-Mas

Reviewer #3 (Remarks to the Author):

Review for the paper 'The Rising Entropy of English in the Attention Economy'

This is an interesting and well-written paper with substantive content. Although there are a handful of passages which I found a bit unclear – see below – it is overall easy to follow. I find the data-driven approach and the results convincing overall. I found the part on information foraging more challenging to follow – there is an accumulation of layers which requires quite some time to assimilate.
I would recommend this paper to be published after some revisions.

Thank you for the supportive comments.

There are several points which I think should be addressed to strengthen the paper:
First, in the whole paper, it is assumed that entropy estimates lexical diversity, and can be considered along other estimations such as the type-token ratio and Zipf exponent. You alternate in particular between the two concepts in the captions of the figures and elsewhere in the manuscript. However, I don't think the relationship is that straightforward - one can see for instance Thoiron 1986, "Diversity Index and Entropy as Measures of Lexical Richness", Computers & The Humanities 20: 197–202. There should be a thorough discussion about this since entropy is at the heart of your approach. This being said, I understand of course the point of having several complementary measurements of the information trends at stake.You could perhaps go further when analyzing the similarities and differences between them. E.g., a and c seem much closer than they are to b.

Thank you for the insightful comment. We agree that our definition of "lexical diversity" was not clear. We note that in the literature lexical diversity is not always used in a consistent way. For example, sometimes the term is used to refer specifically to type token ratio, and other times refers to a more vague concept

that is partially measured by word entropy, Zipf exponent and type token ratio (Bentz et al, 2017). In the interests of being more precise, we have changed the results to avoid reference to “lexical diversity” and described the observed trends in terms of the measures themselves, with a focus on word entropy.

When discussing Figure 1, which shows time series across all 3 measures, we have used the more general phrase “broadening of word distributions”.

Thank you for highlighting the paper by Thoiron, which we feel gives important examples of situations where word entropy and type token ratio measures diverge (e.g. Thoiron generates a text sample by duplicating an existing sample. The word entropy of the duplicated sample is equivalent to the singular sample, while the type token ratio of the duplicated sample is half that of the singular sample.) These examples helped to clarify our own thinking. On reflection, we note that any univariate measure of a frequency distribution will not capture the full information in general, as the distribution has many degrees of freedom. However, univariate measures can capture the full information when a distribution is sufficiently constrained e.g. by being Zipfian, in which case all 3 measures are sufficient statistics and capture the full information of the distribution (as well as being perfectly correlated with each other). In real-world empirical word distributions, which are constrained by being approximately Zipfian, the three measures do not capture all of the information but they will capture a lot of the information, and accordingly the measures have been shown to be well correlated with each other across text samples (Bentz et al, 2015). We note that the examples given by Thoiron are produced by repeating text samples, which will tend to create artificial word distributions that have quite different distributions than real-world test samples and are not Zipfian. Overall we feel that the use of the 3 measures is a good way to give a rounded picture of variation in word distributions. We have added a paragraph in the Limitations section to explore those ideas, where we now cite Thoiron.

Added to the Limitations (page 12):
“Our findings are primarily concerned with differences in word entropy, which is a measure on word distributions. To give a fuller account of these changes, we also analysed the type token ratio and Zipf exponent. It should be noted that these are unidimensional measures of word frequency distributions and do not capture all of the information of those distributions. As such, these measures can diverge significantly with artificially generated text samples (Thoiron, 1986). At the other extreme, if word distributions are tightly constrained (e.g. by being perfectly Zipfian) then the measures are all statistically sufficient in that they capture the full information of the distribution (and correlate perfectly with each other). In empirical samples of natural languages these measures correlate well (although not perfectly), and have been grouped together as a measure of "lexical diversity" (Bentz et al, 2015). Overall we feel that including analyses of the word entropy,

type token ratio and Zipf exponent give a rounded picture of changes in word distributions."

Also, around 1960, there seems to be a drop before a strong increase for all your three measures. Any idea what it could be?

Thank you for pointing that out. After investigating we discovered that this drop was an artifact of the time series smoothing method. We had previously set a criteria of only including data in each media category where there were at least 10 observations within the time series smoothing window (as reported in Materials and Methods). There were some years in the 1960s with low numbers of samples in the News category, which were excluded from the average calculation. While this behaviour was intended, on reflection it gives an unintended artifact of a rise and then drop in the average over that period. In light of this, we relaxed the criteria to require a minimum of 5 data points within the time series smoothing window to be included in the visualisation. Under this criteria the news Category is included in all years in this period and there is no longer the artifact of a rise and drop in the average across categories. We also updated the Materials and Methods section to reflect this change.

Further with respect to entropy, you never discuss conditional entropy, although it would seem a better estimator of information than the non-conditional entropy you use, and some papers also investigating information in languages have relied on it. I would suggest you at least explain why non-conditional entropy is good enough, but getting results with let's say bigram entropy for the sake of comparison would make your case stronger. An issue with conditional entropy appears with very short-from media, because of the many transitions between the messages you assemble into 2000-word texts. But maybe this is still ok, and in any case, you can still work with the other corpora.

Thank you. We have included an analysis of the text samples with bigram entropy, and included these in the supplementary information. This includes both a time series across media categories and the snapshot distributions across media categories and text corpora. We found a similar historical trend of rising bigarm entropy in all categories, and a similar trend of higher bigram entropy in shorter form media categories. The trends are not as clear as with unigram entropy. We did not consider conditional entropy, for reasons outlined below.

We note and agree that there is difficulty in measuring conditional entropy with small sample sizes. We feel that this also goes beyond the particular difficulty with collation of texts in very short-form media. Specifically, the conditional entropy calculation involves bigram and unigram probabilities, which have different scales of bias with small sample sizes. These biases are particularly problematic with natural language samples due to their heavy tailed nature. For example, typically an empirical bigram distribution will have many bigrams that are sampled just

once. There are relatively more of these than the unigram distribution. In effect, the bigram entropy estimate is quite close to the maximum bigram entropy, where each word is observed just once. As such, the empirical unigram distribution is likely to reflect any underlying increases more so than the empirical bigram distribution, so that the two empirical entropies change at different rates as a function of changes in the entropy of an underlying probability distribution. The biases of the measures, and rate of change of the measures as a function of underlying entropy, are at different scales. As $H(Y|X) = H(X,Y) - H(X)$, the changes in empirical conditional entropy are made up of a mix of different factors, which include underlying entropies and biases in the unigram and bigram probability estimation. Overall for this reason we feel that the empirical conditional entropy is not a reliable measure for comparison of text samples.

We recognise that this is not necessarily in line with the existing literature that uses conditional entropy to compare text samples. We note that in some cases the existing literature uses Kneser-Ney smoothing, which is a more accurate form of estimating unigram and bigram probabilities from limited text samples. However, entropy estimation from Kneser-Ney language models is also a more complicated and therefore potentially obfuscating measure. Considering the concerns described in the paragraph above, we do not feel that this method would adequately address variation due to varying biases between unigram and bigram probability estimates. As such, we did not include an analysis with conditional entropy.

We added some paragraphs to the Limitations section to discuss the above.

We added to the Limitations (page 12):
"The measurement of entropy from a finite sequence is a difficult problem, with many approaches of varying degrees of complexity. For example, Lempel-Ziv compression ratios converge to entropy rates with infinite sequences produced from an ergodic source (Ziv & Lempel, 1978). However, language is not ergodic and we do not have infinite sequences. We decided to use the simplest and most well known form of entropy estimation with the maximum likelihood or plug-in estimator to find empirical unigram entropy. Our choice is supported by work that shows that this measure correlates well with more advanced estimators (see Betnze et al (2017) for a comparison of entropy estimators). In the Supplementary Information: Extended Data, we extend the analysis to bigram entropy using the maximum likelihood estimator, with broadly similar trends. We considered analysing the conditional entropy, but were concerned that this involves the combination of estimates of both unigram and bigram probabilities, which have varying potential degrees of bias as a function of sample size and the underlying distribution. Estimates of conditional entropy therefore include a mix of variation in a) the underlying distribution as well as b) unigram probability biases and c) bigram probability biases. We are not able to control for these biases and as such

we feel that this is not a suitable form of analysis. We considered using a Kneser-Ney language model (Kneser & Ney, 1995) to overcome this issue but we decided that any accuracy benefits were outweighed by the obfuscation created by this more complicated model. We support future work that investigates other measures of entropy.”

When you perform 24 trend tests for the COHA texts, their four categories and the three different measures adopted, you do not perform any adjustment for multiple testing. There should be some control of the increased rate of false positives here, for instance with Holm-Bonferroni correction if you want to control for the family-wise error rate. The same applies, although there are less tests, to the various ANOVA you rely on to investigate the differences between media categories. My intuition is that correcting the p-values will lead to some of the 24 tests not returning significant results anymore, but that doesn't seem to be a very big issue.

Thank you for pointing this out. We applied a Holm-Bonferroni correction to the trend tests. We found that all 24 tests were still significant with a significance level of 0.05, and 21 out of 24 were significant at 0.01. (previously 22 out of 24 were significant at 0.01).
During the course of applying the Holm-Bonferroni correction, we also calculated more precise p-values (where p<0.01) for both the MK and KPSS tests. These are reported in the SI.

We also applied a Holm-Bonferroni correction to the ANOVA tests, and found that these were all still significant at significance levels of both 0.05 and 0.01.

We added an explanation of the Holm-Bonferroni corrections in the Methods and Results sections. And the Extended Results section in the Supplementary Information.

In regards to the trend tests. Added to Materials and Methods (page 3):
“We carried out a total of 24 hypothesis tests for trends. To adjust for multiple tests, we applied a Holm-Bonferroni correction to adjust the significance level.”

In regards to the ANOVA tests. Added to MAterials and methods (page 4):
“We also applied a Holm-Bonferroni correction to control for the familywise type 1 error rate while carrying out multiple hypothesis tests.”

I am not sure your whole approach to ‘information foraging in the attention economy’ should be part of the ‘Results’ section of your paper. The point here is that you don't really use any of your initial data in this approach, but mostly provide an interpretative framework (which includes simulated data) for the (recent) trends you observe. As I am not familiar with information foraging (beyond the general idea of a modulation of selectivity in response to the environment), I found this part more challenging than the first one – at least at the level of the finer details.

We thank you for the suggestion and recognise that it would be helpful to separate the results and the model, which we have now separated into a dedicated "Model" section.

I also wonder whether it would be possible to go further and conceive of experiments with participants faced with different texts/sources of information. This could perhaps be interesting to point to, even if very briefly.

Thank you. We would very much like to see (or run ourselves) behavioural experiments that test which factors lead people to choose sources of information. We think that this is a research gap and is very important given the modern media landscape and the attention economy. We added this as a suggestion in the Limitations section.

Added to the Limitations (page 13):
"There is much further work needed to understand how people choose what information to consume, and how that affects their behaviour. Modern technology creates opportunities for new experiments. While eye-tracking experiments (Itti & Baldi, 2009; Boerman et al, 2011; Radach et al, 2003) are well established, we can also characterise and measure attention in terms of cognitive mechanisms (Carretie et al, 2001; Vuilleumier, 2005; Baek & Parkinson, 2022). Beyond attention, we can ask how beliefs are influenced through factors such as emotive (Wearn & Shepherd, 2020) or narrative *Yang & Hobbs, 2020) descriptions. An approach that is aligned with attention economy questions is to measure behavioural engagement while carrying out tasks, either in researcher-designed websites (Redlawsk, 2002) or in naturalistic settings (Mark, 2023)"

Regarding the discussion on the Linguistic Niche Hypothesis and the influence of second language learners, I think a more explicit historical/time perspective is missing: what is/are the relevant timescale(s) for morphological simplification and increase in the number of L2 speakers? The British Empire was at its height in the 19th and early 20th century. How did that translate in terms of number of L2 speakers? Was morphological simplification already at work then, or is it a more recent phenomenon?What you write in the first part of the Supplementary Information could perhaps find its place in the main text to expand and better articulate the first part of the discussion. The content does not sound very 'supplementary information' to me, but I guess there is a constraint of space here.

Would you have any simple quantitative strategy to assess morphological complexity in your various texts that departs from lexical diversity and TTR – especially with the COHA corpus? The following paper would I think be a good starting point: Çağrı Çöltekin & Taraka Rama (2022). What do complexity measures measure? Correlating and validating corpus-based measures of morphological complexity. It would much strengthen your argumentation to have

quantitative data for both entropy/lexical diversity and morphology and look for possible correlations (either positive or negative).

Thank you for your comments. We explored some of the suggestions that you raised. We agree that the connection to the Linguistic Niche Hypothesis is not really supplementary information and would merit an article in itself. Reviewer 1 also mentions this, and after some reflection and discussions, we believe that the connection to morphological complexity and the linguistic niche hypothesis is more complicated than we are able to explore in a concise way within this study. We feel that the best approach is to scale back our ambitions in terms of drawing direct connections to the linguistic niche hypothesis. This focuses the study on our finding of changes in word entropy and the attention economy model. We feel that this improves the current study as a standalone piece of work.

We think that there is a particular difficulty, and area for future study, in assessing whether associative relationships between linguistic measures apply across all domains and between all types of text. For example, the paper by Çöltekin & Rama (2022) compared text measures across different languages. This was also the approach used in some papers referenced in that study (e.g.. Juola, 2008; Bentz et al, 2016; Kettunen, 2014). We are very interested in whether these associations always apply when comparing text samples within languages, and in which conditions they no longer apply. For example, in our study we argue that there is an increasing pressure towards expressivity in English, which may interact with other pressures such as those described in the linguistic niche hypothesis. It is unclear whether cross-language associations will still hold in languages under these pressures. While interesting, we feel that this is beyond the scope of the current study.

In light of this, we have reduced our comments on the connections to the Linguistic NIche Hypothesis and morphological complexity to a single paragraph in the Discussion and removed the section in the Supplementary Information entirely.

Added to the Discussion (page 10):
Our findings offer an interesting additional perspective on pressures involved in linguistic evolution. For example, the Linguistic Niche Hypothesis (Lupyan & Dale, 2010) predicts a loss of complex morphological forms in English due to the influence of second language learners. Indeed, there is ample evidence that English is undergoing morphological simplification (Michel et al, 2011; Lieberman et al, 2007; Zhu & Lei, 2018). If we can summarise the effect of the Linguistic Niche Hypothesis as a pressure towards learnability, then our study claims an additional pressure towards expressivity. One might expect these two dynamics to be in opposition, as simpler symbolic rules would a priori be less expressive per symbol and therefore have lower entropy (Mackay, 2003). However, a more

nuanced analysis can take into account the fact that human language includes redundancy (Shannon, 1951), in which case a pressure towards learnability through simplification by reduction of this redundancy would simultaneously increase entropy. While we can speculate about the interaction of these pressures (and other factors), we encourage further work in this area.

Other comments:

Line 41: How close are entropy and information density? What does specifically mean exactly here?

Thank you for pointing this out. We have clarified the link between information density and entropy.
"Specifically, we quantify increasing information density as higher entropy, reflecting more diverse and less predictable information."

Lines 81-83: are we sure there are no elements at the end of the texts which can also cause problems? This is something to account for, for instance, in Project Gutenberg text files, but I guess not with your corpora.

Thank you for pointing this out. We did find that some of the fiction text samples had some elements at the end of text such as Copyright and author information. This is usually only a few words.
We refined our data cleaning process to take samples from the middle of documents, with a criteria of a text sample length of at least 2100 tokens for a 2000 token sample. In the vast majority of cases this will avoid any anomalous text at both the beginning or end.

Lines 116-118: I agree that the analysis is not flawed. I guess the question is mostly about how one can effectively measure the information density of a social media.

Thank you.

Lines 121-122: If we know the (shape of the) relationship between lexical measures and sample size, could we account for it? I guess it doesn't matter since all of your texts are of the same size, but maybe you could specify the shape of the relationship if it is known and stable. Also, is 2,000 words a small size given the mentioned sensitivity to size?

Thank you. Yes we did investigate the feasibility of accounting for the shape of the relationship between lexical measures and sample size. We came to the conclusion that doing so brings in extra assumptions that are not necessary when comparing text samples of the same size, when considering comparisons between samples. We added a discussion of the convergence relationships in the Limitations section. We also justified the choice of 2000 words as balancing having a large number of samples without those samples being too small.

Added to the Limitations (page 12):

"The text measures used are all sensitive to sample size. We investigated a process of extrapolating the measures to allow comparisions across varying sample sizes. For example, unigram word entropy measures converge at about N=50,000 tokens and have relatively stable convergence curves as a function of number of tokens (Bentz et al, 2017). And Zipf estimators have systematic positive biases as functions of the exponent and sample size (Pilgrim & Hills, 2020). However, this kind of extrapolation would bring in extra assumptions that may not apply universally across time or text categories. To control for sample size sensitivity we therefore chose to truncate text samples to N=2,000 words, which is appropriate as we are interested in comparisons rather than absolute estimations."

Lines 124-126: given the importance of the concept of entropy in your study, maybe you should write more about the plug-in estimator versus more advanced estimators.

We added a paragraph in Limitations to discuss other forms of entropy estimation, and justify our choice as being simple and correlated with other measures.

We added to the Limitations (page 12):
"The measurement of entropy from a finite sequence is a difficult problem, with many approaches of varying degrees of complexity. For example, Lempel-Ziv compression ratios converge to entropy rates with infinite sequences produced from an ergodic source (Ziv & Lempel, 1978). However, language is not ergodic and we do not have infinite sequences. We decided to use the simplest and most well known form of entropy estimation with the maximum likelihood or plug-in estimator to find empirical unigram entropy. Our choice is supported by work that shows that this measure correlates well with more advanced estimators (see Bentz et al (2017) for a comparison of entropy estimators). In the Supplementary Information: Extended Data, we extend the analysis to bigram entropy using the maximum likelihood estimator, with broadly similar trends. We considered analysing the conditional entropy, but were concerned that this involves the combination of estimates of both unigram and bigram probabilities, which have varying degrees of bias as a function of sample size and the underlying distribution. As such, variation in estimates of conditional entropy include variation in the underlying distribution as well as variation in the degrees of unigram and bigram probability biases, and as such we feel that this is not a suitable form of analysis. We considered using a Kneser-Ney language model (Kneser & Ney, 1995) to overcome this issue but we decided that any accuracy benefits were outweighed by the obfuscation created by this more complicated model. We support future work that investigates other measures of entropy."

Lines 123-126 and 1st formula: (Empirical unigram word) Entropy is based on the distribution of words in a given text. Yet, at least for the most recent time periods, one could consider the

frequencies of words as assessed in much larger text corpora, and rely on these frequencies to compute the entropy of the target texts. Does that make sense? Given that experienced readers have been exposed to a very large number of texts, maybe at a cognitive level the word frequencies that matter are those derived from these many texts, and not those specific to a 2000-word text)? Would we get similar results with this approach? That seems interesting to investigate.

> **Thank you for the suggestion. That would indeed be an interesting analysis. We hypothesise that we would get similar results when analysing larger corpora. However, when it comes to comparing text corpora there is a difficulty in managing confounding factors that influence the text samples that we have available. This is especially true when looking at historical corpora.**
>
> **We added a note in the Limitations section to recognise the limitations of using text samples as opposed to analyses over larger corpora.**
>
> **Added to the Limitations (page 11):**
> ***"When using big data to analyse culture, a key question to ask is whether we are detecting variation in the underlying population of interest (in our case media expression of language) or variation in the data source (in our case text corpora). While this is an inherent problem when studying changes in language, we took steps to ameliorate the issue. This included the choice of COHA, which is a balanced corpora, with representative text samples across media categories. In contrast, we investigated but did not choose to use Google Ngrams due to question marks surrounding variation in the corpus composition over time (Pechenick et al, 2015}. Given the use of COHA, there was a challenge in balancing the benefits of large text samples with the number of text samples available. We found that a target size of N=2000 words gives a good balance between sample length and quantity of samples. We also added robustness checks when comparing text samples across media categories by using multiple corpora. More generally, we note that big data corpus analysis is not a replacement for traditional cultural sociology techniques, but a complement (Janicke et al, 2015; Bail et al, 2014) and we encourage future work along more traditional qualitative lines (Sun et al, 2021)."***

Lines 148-150: I am not entirely convinced the influence of outliers gets smaller with this approach, as the 'first level' of averaging is still sensitive to them, and then you only take the average of 4 categories, not 20 or 100.

> **Yes we agree. We used the mean primarily because it was a more natural measure to use when combining categories than a median. We agree that outliers can still exert an influence when averaging over 4 categories. We have removed this aspect of the justification from the text.**

Lines 189-192: Maybe explain what's the point of looking at this, especially since it only appears in the Supplementary Information

We added a justification for looking at the magazine industry, in order to place the linguistic changes in the context of changes in media publishing over the 20th century. While this adds some context and connection to the real-world, we feel it belongs in the SI because it is a more qualitative analysis than the main focus of the paper, and could be a distraction in the main article.

Added to Materials and Methods (page 4):
"In order to explore connections between word entropy and qualitative changes in media publishing, we explored a case study of the US magazine industry in the Supplementary Information: Historical Analysis of US Magazine Publishing."

Lines 231-233: Shannon's idea here seems to refer to conditional entropy, no? ('given the preceding symbols'). You may want to be clearer here.

Thank you. We have clarified our meaning here, to be more in line with Shannon's 1948 paper "A Mathematical Theory of Communication".
Added to page 6:
"The entropy of a source of information is a function of the probability of seeing each symbol. This function is equivalent to the expected reduction in uncertainty when receiving symbols, defined as a rate of information per symbol. "

Line 233: entropy can be thought of as a rate of information: I think this deserves more explanations, as you assimilate a 'static' measure to a non-static / time-related one.

Thank you very much for pointing this out. This is an assumption that was not clear in our model description. We clarified the description to explicitly acknowledge the link between the static rate of information per symbol with the dynamic rate of utility from that information.

We updated this paragraph to read (page 6):
"The previous results are suggestive of a link between competition for attention and word entropy. To explain these results we generate a model of the attention economy based on information foraging. Foraging models relate the consumption of information items with some utility gain to the forager. To bridge utility rates to lexical measures, we borrow the idea of information signal entropy from Shannon (1948): The entropy of a source of information is a function of the probability of seeing each symbol. This function is equivalent to the expected reduction in uncertainty when receiving symbols, defined as a rate of information per symbol. While this is a static measure, it also reflects the potential utility gain per unit of time as foragers encounter and process information, with the assumption that an increase in entropy, h, is associated with an increase in utility rate, r. This aligns with Zipf's principle of least effort (Zipf, 1949; Ferrer-i-Cancho, 2005)."

Lines 236-240: Maybe you could further explicitly report on possible additional factors guiding the selection of information in humans besides information density. You mention some factors at the very end of your discussion, but maybe this should come earlier and be expanded. Could you actually account for this in your model – which would have further novelty to your approach?

Thank you for highlighting this. We included other factors that have been revealed in the literature in a paragraph in the Limitations section.

We did not incorporate this in the present model, but have suggested this as an avenue for further research.

Added to the Limitations (page 13):
"Beyond information density (Itti & Baldi, 2009; Radach, 2003), there is evidence that human attention is also attracted to information that is belief-consistent (Hills, 2019; Taber & Lodge, 2006; Hart et al, 2009), predictive (Hills, 2019), social (Hills, 2019; Davis & McLeod, 2003), negative (Hills, 2019; Davis & McLeod, 2003, Lang et al, 1990) and emotive (Vuilleumier, 2005; Anderson, 2005; Saunders, 2020). Our model does not account for these other dimensions, and instead we make the assumption that aggregate behaviour can be usefully understood by considering the dimension of information density in isolation. A similar assumption is made in food foraging models, which usually focus on net calorie intake (Stephens & Krebs, 1986) while omitting other important factors such as macronutrient content (Hill, 1988) and predation risk (Verdolin, 2006). However, just as food foraging models have their limitations in capturing the multi-faceted nature of foraging, so does our model in capturing the intricacies of information search. Future work could explore how various factors, including word entropy, attract human attention. This could extend to considering how different factors might interact. For example, the extremely attention grabbing utterance "FIRE!" is at once concise, predictive, negative and emotive."

Line 338: maybe explain a bit more 'cooling by expansion'

We agree this is unclear. We wanted to reference Petersen's paper as it is an innovative approach to understanding language evolution. But it is only tangentially related to the current work and so we do not feel that a full explanation is warranted in the discussion. Petersen's study is based on changes in linguistic evolution in large language distributions, which was described in that paper as "cooling by expansion", which is a physics term. We recognise that this is not clear as we explained it. We have kept the reference but described it under the term " word formation and distribution ", which is more descriptive and provides a clearer link to linguistic concepts in our study.

Lines 343-344: if people's attention is attracted to high complexity information, why not be attracted to morphological complexity?...

That is a good point, and we thank you for making it. After some reflection, we feel that the link to morphological complexity is ambitious for this article, and so we have decided to focus on the changes in entropy, and have removed some of the more speculative claims about how the findings relate to morphological simplification.

Each time you mention elements in the Supplementary Information, maybe it would help to provide more specific references to the sections and figures?

Thank you for the suggestion. We have made that change.

In the Supplementary Information, lines 60-62: indeed Pearson's r correlation does not seem appropriate here due to temporal autocorrelation. Not entirely sure, but maybe you could consider a generalized least squares model with a correlation structure like AR1?

Thank you for the suggestion. We agree it is helpful to explore other measures here. We fit an AR1 model, finding a positive coefficient, which we reported in the SI.

Following your comment we also thought about other ways to account for time as a confounder. We explored a simple method of detrending the data by taking the difference in monthly circulation and word entropy in 5 year periods (which is the resolution of the circulation data). We did not find a significant association, which we also reported in the SI.

Updated in the SI: Historical Analysis of US Magazine Publishing:
"Word entropy continues to rise throughout the 20th century alongside magazine circulation, with a Pearson's correlation coefficient r=0.91, p < 0.001), although both rise over time so that confounding factors are not ruled out (Figure 1). We explored further with a Generalized Linear Model with an AR(1) component to account for autocorrelation in the data, finding that magazine circulation was a significant predictor of word entropy in this model (coefficient = 0.0004, p<0.001). However, we did not find a significant correlation when the time series was detrended by analysing associations in the differences in magazine circulation and word entropy over 5 year periods, (Pearson's correlation coefficient r=-0.094; p=0.72)."

Section 2 of the Supplementary Information could perhaps find its place in the paper too (unless you have a strong word limit), since it provides interesting information about how one can observe clear signals of economic transformations in the evolution of entropy.

Thank you for the suggestion. We agree that the analysis of the magazine industry is an interesting perspective, and provides some useful historical context in

showing how the attention economy manifested in increasing magazine circulation and specific innovations in that industry. However, we feel that it is important to keep the focus on a) historical linguistic trends and b) the model. Including the magazine industry analysis in the main paper, while interesting, may detract from the clarity of the message of the paper.

Minor comments:

All p in ‘p-value(s)’ should appear in italic.

DONE.

Line 109: Removed any urls -> Removed any url

DONE. Changed to “remove any website and email addresses”.

Line 110: usernames i.e. any words containing usernames, i.e., any word containing

DONE

Line 127: by the total words -> by the total number of words

DONE

Line 133: widely used and well known -> well known and widely used

DONE

Line 134: comparision -> comparison

DONE.

Line 141: These were plotted -> a bit unclear what ‘these’ refer to

DONE. Changed to “the medians were plotted”.

Line 147: we first took means -> we first took the means (?)

DONE

Line 172: as well as the categories -> as well as for the categories

DONE

Lines 189-191: This sentence is not very clear (some data attributed to other data?)

DONE. Simplified.

Line 192: The data was plotted without further treatment -> which figure are you referring to here?

DONE. We referred to the figure

Caption of Figure 1: Shaded region shows 95% confidence interval of the average Shaded regions show 95% confidence intervals for the different averages (not sure, but since you have three panels and thus three confidence intervals.

DONE. Clarified a different confidence interval and average for each panel.

Line 205: And -> and

DONE

Line 206: of trends at -> of a trend at (same as just before?)

DONE

Line 211: I am not sure it is the trend itself which suggests that.

DONE. Clarified

Line 212: has become -> have become (?)

DONE

Line 218: ANOVA test $p < 0.01$ -> ANOVA tests $p < 0.01$

DONE

Line 219: short-form media categories -> the short-form media categories

DONE

Lines 221-226: you have already said that, maybe there’s no need to repeat it (and it will save some space for something else)? The last sentence is clear and impactful, though, I’d suggest you keep it somewhere.

DONE.

Line 228: The results -> The previous results (?)

DONE

Lines 243-244: The derivation… -> unclear, maybe rephrase

DONE. Rephrased to “the description”

Lines 333-334: of more difficult to process linguistic forms -> grammatical issue

DONE. We have removed this section of the Discussion in response to other comments.

Good luck and thanks for an interesting read.

Christophe Coupé (Department of Linguistics, The University of Hong Kong)

15th May 24

Dear Charlie,

Your manuscript titled "The Rising Entropy of English in the Attention Economy" has now been seen by our reviewers, whose comments appear below.

I am sincerely sorry for the long delay - not all referees returned for the re-review of your revision, and recruiting suitable experts to vet your revision took longer than we would have hoped.

Now that we have obtained the necessary feedback and, I am delighted that in light of the advice, we are happy, in principle, to publish a suitably revised version in Communications Psychology under the open access CC BY license (Creative Commons Attribution v4.0 International License).

We therefore invite you to revise your paper one last time to address the remaining concerns of our reviewers and a list of editorial requests. At the same time we ask that you edit your manuscript to comply with our format requirements and to maximise the accessibility and therefore the impact of your work.

EDITORIAL REQUESTS:

Please review our specific editorial comments and requests regarding your manuscript in the attached "Editorial Requests Table". Please outline your response to each request in the right hand column. Please upload the completed table with your manuscript files as a Related Manuscript file.

If you have any questions or concerns about any of our requests, please do not hesitate to contact me.

SUBMISSION INFORMATION:

In order to accept your paper, we require the files listed at the end of the Editorial Requests Table; the list of required files is also available at https://www.nature.com/documents/commsj-file-checklist.pdf .

OPEN ACCESS:

Communications Psychology is a fully open access journal. Articles are made freely accessible on publication under a CC BY license (Creative Commons Attribution 4.0 International License). This license allows maximum dissemination and re-use of open access materials and is preferred by many research funding bodies.

For further information about article processing charges, open access funding, and advice and support from Nature Research, please visit https://www.nature.com/commspsychol/article-processing-charges

At acceptance, you will be provided with instructions for completing this CC BY license on behalf of all authors. This grants us the necessary permissions to publish your paper. Additionally, you will be asked to declare that all required third party permissions have been obtained, and to provide billing information in order to pay the article-processing charge (APC).

* TRANSPARENT PEER REVIEW: Communications Psychology uses a transparent peer review system. On author request, confidential information and data can be removed from the published reviewer reports and rebuttal letters prior to publication. If you are concerned about the release of confidential data, please let us know specifically what information you would like to have removed. Please note that we cannot incorporate redactions for any other reasons.

* CODE AVAILABILITY: All Communications Psychology manuscripts must include a section titled "Code Availability" at the end of the methods section. We require that the custom analysis code supporting your conclusions is made available in a publicly accessible repository at this stage; please choose a repository that generates a digital object identifier (DOI) for the code; the link to the repository and the DOI must be included in the Code Availability statement. Publication as Supplementary Information will not suffice.

* DATA AVAILABILITY:

All Communications Psychology manuscripts must include a section titled "Data Availability" at the end of the Methods section. More information on this policy, is available in the Editorial Requests Table and at http://www.nature.com/authors/policies/data/data-availability-statements-data-citations.pdf.

Please use the following link to submit the above items:

[link redacted]

** This url links to your confidential home page and associated information about manuscripts you may have submitted or be reviewing for us. If you wish to forward this email to co-authors, please delete the link to your homepage first **

We hope to hear from you within two weeks; please let us know if you need more time.

Best regards,

Marike

Marike Schiffer, PhD

Chief Editor

Communications Psychology

REVIEWERS' COMMENTS:

Reviewer #3 (Remarks to the Author):

Dear authors,

Thank you for your clear, honest and detailed replies to my (and other reviewers') comments and suggestions. I am happy with them and do not have further requests. Great that accounting for multiple tests did not 'erase' any result. I also understand that you felt it was better to 'scale down' when it comes to the Linguistic Niche hypothesis and morphological complexity. Leaving aside conditional entropy makes good sense.

You might want to check the manuscript again for typos - I found at least two, lines 403 and 388.

Line 178, family-wise might be better than familywise.

All the best,

Christophe Coupé

Reviewer #4 (Remarks to the Author):

I learned something new here about changes in textual density in English over time, and how the Zipf coefficient can be used meaningfully.

I should point out to the editor that my mathematical understanding is just enough to follow the author's argument, but I am not equipped to comment critically on the math.

The author should please include an explanation why the social media texts were grouped in 2000 word chunks, and the potential effect that this decision has on the various textual density measures that are applied.

In addition, allow me to point out that the words corpus (singular) and corpora (plural) should be used more carefully, I encountered a few errors. Likewise, I suggest that the author should retrieve all instances of effect and affect (verbs) in the text and make sure they are correctly employed. Currently, there are some errors.

Overall, I vote for publication of the manuscript after minor copyediting.

We thank the reviewers and editors for their time and consideration.

We have responded to the reviewer comments in bold.

REVIEWERS' COMMENTS:

Reviewer #3 (Remarks to the Author):

Dear authors,

Thank you for your clear, honest and detailed replies to my (and other reviewers') comments and suggestions. I am happy with them and do not have further requests. Great that accounting for multiple tests did not 'erase' any result. I also understand that you felt it was better to 'scale down' when it comes to the Linguistic Niche hypothesis and morphological complexity. Leaving aside conditional entropy makes good sense.
Thank you for the positive assessment.

You might want to check the manuscript again for typos - I found at least two, lines 403 and 388. Line 178, family-wise might be better than familywise.
Thank you, we have made those corrections and checked the manuscript for other corrections.

All the best,

Christophe Coupé

Reviewer #4 (Remarks to the Author):

I learned something new here about changes in textual density in English over time, and how the Zipf coefficient can be used meaningfully.
Thank you for the positive assessment.

I should point out to the editor that my mathematical understanding is just enough to follow the author's argument, but I am not equipped to comment critically on the math.

The author should please include an explanation why the social media texts were grouped in 2000 word chunks, and the potential effect that this decision has on the various textual density measures that are applied.
Thank you, we included this explanation in the Methods section “The lexical measures are all sensitive to sample size. We therefore chose to truncate text samples to N=2,000 words, and discarded text samples with less words. This word count strikes a good

balance of being a large enough sample size to detect variation between texts, while being small enough that we have a large number of surviving texts in the corpora.“

In addition, allow me to point out that the words corpus (singular) and corpora (plural) should be used more carefully, I encountered a few errors. Likewise, I suggest that the author should retrieve all instances of effect and affect (verbs) in the text and make sure they are correctly employed. Currently, there are some errors.
Thank you. We carefully corrected those instances that were not used appropriately.

Overall, I vote for publication of the manuscript after minor copyediting.
